# SALL4 promotes angiogenesis in gastric cancer by regulating VEGF expression and targeting SALL4/VEGF pathway inhibits cancer progression

**DOI:** 10.1186/s12935-023-02985-9

**Published:** 2023-07-31

**Authors:** Fatma A. Abouelnazar, Xiaoxin Zhang, Jiahui Zhang, Maoye Wang, Dan Yu, Xueyan Zang, Jiayin Zhang, Yixin Li, Jing Xu, Qiurong Yang, Yue Zhou, Haozhou Tang, Yanzheng Wang, Jianmei Gu, Xu Zhang

**Affiliations:** 1 Jiangsu Key Laboratory of Medical Science and Laboratory Medicine, School of Medicine, Jiangsu University, Zhenjiang, 212013 Jiangsu China; 2 Department of Clinical Laboratory Medicine, Affiliated Cancer Hospital of Nantong University, Nantong, 226300 China

**Keywords:** Gastric cancer, Angiogenesis, SALL4, VEGF, CRISPR/Cas9, Thalidomide, Exosomes

## Abstract

**Background:**

Spalt-like protein 4 (SALL4) is a stemness-related transcription factor whose abnormal re-expression contributes to cancer initiation and progression. However, the role of SALL4 in cancer angiogenesis remains unknown.

**Methods:**

Analyses of clinical specimens via TCGA datasets were performed to determine the expression level and clinical significance of SALL4 in STAD (Stomach Adenocarcinoma). SALL4 knockdown, knockout, and overexpression were achieved by siRNA, CRISPR/Cas9, and plasmid transfection. The effects of conditioned medium (CM) from SALL4 knockdown or overexpression of gastric cancer cells on endothelial cell proliferation, migration, and tube formation were investigated by CCK-8 assay, transwell migration assay, and tube formation assay. The regulation of VEGF gene expression by SALL4 was studied by qRT-PCR, western blot, chromatin immunoprecipitation (ChIP) assay, and electrophoretic mobility shift assay (EMSA). Engineered exosomes from 293T cells loaded with si-SALL4-B and thalidomide were produced to test their therapeutic effect on gastric cancer progression.

**Results:**

SALL4 expression was increased in STAD and positively correlated with tumor progression and poor prognosis. SALL4-B knockdown or knockout decreased while over-expression increased the promotion of human umbilical vein endothelial cells (HUVEC) cell proliferation, migration, and tube formation by gastric cancer cell-derived CM. Further investigation revealed a widespread association of SALL4 with angiogenic gene transcription through the TCGA datasets. Additionally, SALL4-B knockdown reduced, while over-expression enhanced the expression levels of VEGF-A, B, and C genes. The results of ChIP and EMSA assays indicated that SALL4 could directly bind to the promoters of VEGF-A, B, and C genes and activate their transcription, which may be associated with increased histone H3-K79 and H3-K4 modifications in their promoter regions. Furthermore, si-SALL4-B and thalidomide-loaded exosomes could be efficiently uptaken by gastric cancer cells and significantly reduced SALL4-B and Vascular Endothelial Growth Factor (VEGF) expression levels in gastric cancer cells, thus inhibiting the pro-angiogenic role of their derived CM.

**Conclusion:**

These findings suggest that SALL4 plays an important role in angiogenesis by transcriptionally regulating VEGF expression. Co-delivery of the functional siRNA and anticancer drug via exosomes represents a useful approach to inhibiting cancer angiogenesis by targeting SALL4/VEGF pathway.

**Supplementary Information:**

The online version contains supplementary material available at 10.1186/s12935-023-02985-9.

## Introduction

Gastric cancer is one of the most common cancers and the second leading cause of cancer-related death worldwide [1, 2]. Due to atypical early symptoms, the majority of gastric cancer patients are diagnosed with advanced-cancer stage, decreasing the chance of resection and resulting in a poor 5-year survival rate [3]. Although the therapeutic effect of gastric cancer has been greatly improved [4], the 5-year survival rate is still not satisfactory [5]. As a result, there is an urgent need for additional research on the pathogenesis of gastric cancer to develop new biomarkers and therapeutic targets, which can help for better diagnosis and treatment.

Tumor blood vessels deliver oxygen and nutrients to tumor tissue, allowing it to grow rapidly and spread to distant locations [6]. Recently, inhibiting cancer angiogenesis has been developed as a new anti-tumor strategy [7]. VEGFs were first identified as vascular permeability factors, which are essential for vessel formation in both physiological and pathological conditions [8]. VEGFs are released by tumor cells and promote vascular leakage [8]. Mammalian VEGF-A, -B, and -C are required for blood vessel formation, whereas VEGF-C and -D regulate lymphatic vessel formation [9, 10].

Spalt-like transcription factor 4 (SALL4) encodes a zinc finger transcription factor that is essential for maintaining embryonic stem cell pluripotency and self-renewal [11]. Overexpression of SALL4 has been reported in a variety of cancers including gastric cancer [12–14]. Numerous studies demonstrate that SALL4 plays a key role in carcinogenesis, cancer metastasis, and cancer therapy resistance [15, 16]. SALL4 is required for embryogenesis but is rarely found in adult tissues [17]. It is well known that vasculogenesis and angiogenesis are required for embryogenesis and play a critical role in the regulation of multiple physiological processes during embryonic development. Furthermore, increased cancer vascularization has been linked to a poor prognosis, and a high proliferative, and metastatic potential [18].

Targeting SALL4 has a promising therapeutic effect on cancer and has the potential to become an effective therapeutic strategy. Previous research has linked high SALL4 expression to a more sensitive response to entinostat treatment in human lung cancer cells [19]. Also, SALL4 knockdown is an important mechanism for cisplatin-induced apoptosis and may restore cisplatin sensitivity in acquired resistant lung cancer cells [20].

In this study, we investigated the biological roles and mechanisms of SALL4 in the pathogenesis of gastric cancer. We found that the upregulation of SALL4 in STAD was positively correlated with tumor progression. Also, we found that SALL4-B downregulation inhibited, while overexpression enhanced, the pro-angiogenic effect of gastric cancer cells. In addition, we also discovered that SALL4 promoted angiogenesis via the regulation of the VEGF gene. Furthermore, we used exosomes as a nanocarrier vesicle to deliver SALL4-B-targeting siRNA and the chemotherapeutic drug thalidomide for the suppression of gastric cancer angiogenesis by inhibiting the SALL4/VEGF pathway. Thus, our findings suggest that SALL4 is critically involved in gastric cancer progression by regulating VEGF and angiogenesis, thus providing a potential target for cancer therapy.

## Materials and methods

### Cell culture

Human gastric cancer cell lines (MKN-45, MGC-803, and HGC-27) and HUVECs were purchased from the Chinese Academy of Sciences’ Institutes for Biological Sciences (Shanghai, China) and maintained in Gibco Roswell Park Memorial Institute (RPMI-1640) or Dulbecco’s modified Eagle (DMEM) medium supplemented with 10% fetal bovine serum (FBS) and 1% penicillin-streptomycin (Gibco, Invitrogen Life Technologies, Carlsbad, CA, USA). All the cell lines were grown in an incubator (37 °C) with 5% CO2.

### Chemicals

Thalidomide (CSN12073) and Puromycin (CSN23421) were purchased from CSNpharm (Illinois, USA).

### Gene transfection

In 6-well plates, the cells were seeded at a density of 2 × 10^5^ cells/well and cultured overnight in a 37 °C incubator. The over-expressing plasmid and silencing siRNAs (Gene Chem, Shanghai, China) were transfected into the cells using Lipofectamine 2000 transfection reagent (Invitrogen; Thermo Fisher Scientific Inc. USA) in a serum-free medium. The cells were switched to a complete medium 6 h after transfection and cultured for another 36 h. The target sequences of siRNAs are shown in (Additional file 1 Table S1).

### CRISPR/Cas9 SALL4 knockout

CRISPR/Cas9 technology was used to create MGC-803 SALL4 knockout cells. SALL4 CRISPR/Cas9 knockout (KO) plasmid (h) (sc-401,033, Santa Cruz Biotechnology, Inc. USA) is a pool of three plasmids, each encoding the Cas9 nuclease and a SALL4-specific 20 nt guide RNA (gRNA) sequence for maximum knockout efficiency. The GeCKO (v2) library is used to generate gRNA sequences that direct the Cas9 protein to induce a site-specific double-strand break (DSB) in the genomic DNA. After incubation, successful transfection of CRISPR/Cas9 KO plasmid may be visually confirmed by detection of the green fluorescent protein (GFP) via Western blot or immunofluorescence (GFP-tag Antibody, T0005, Affinity Biosciences, USA). Co-transfection with SALL4 HDR Plasmid (h) (sc-401,033-HDR, Santa Cruz Biotechnology, Inc. USA) is recommended for Puromycin (10 µg/mL) selection of cells containing a successful Cas9-induced DSB.

### Western blotting

A RIPA buffer containing 1% protease inhibitors was used to lyse the cells. SDS-PAGE was used to separate the protein sample, followed by the transfer to PVDF membranes. The membrane was blocked with 5% bovine serum albumin and then incubated overnight with specific antibodies against SALL4 (ab29112, Abcam), VEGF-A (66828-1-Ig, Proteintech), VEGF-B (YT4871, Immunoway), VEGF-C (22601-1-AP, Proteintech), and GAPDH (MB001; Bioworld Technology, St. Louis Park, MN, USA). After 2 h of incubation with the secondary antibodies (Bioworld Technology) at room temperature, the bands were visualized with a chemiluminescent detection system.

### RNA extraction and quantitative real-time PCR (qRT-PCR)

Total RNA was isolated using Trizol (Invitrogen Life Technologies) reagent from gastric cancer cells according to the manufacturer’s instructions. For cDNA synthesis, the isolated RNA was reverse-transcribed using the HiScript reverse transcription kit (R312-01/02, Vazyme Biotech, Nanjing, China). The cDNA was then subjected to qRT-PCR analyses using SYBR green on a Bio-Rad CFX96 system. The 2^−ΔΔCt^ method was used to determine mRNA fold changes. β-actin served as a normalization control. The primer sequences are listed in (Additional file 1 Table S2).

### Conditioned medium (CM) collection

The transfected and treated cells were cultured in RPMI-1640 containing 10% FBS and plated on a 6-well plate (2 × 10^5^ cells/dish). The cells were washed after 24 h and changed from normal growth medium to serum-free medium. After 48 h of incubation, cell culture supernatants were collected and centrifuged at 2000 rpm to remove cell debris.

### Cell counting Kit-8 (CCK-8) assay

Cells were seeded in 96-well plates (2000 cells/well), and cultured for the indicated time. Cell growth was measured by incubating cells with CCK-8 solution according to the manufacturer’s instructions (A311-01, Vazyme Biotech). The absorbance at 450 nm was then measured using a microplate reader. The experiment was done in triplicate.

### Tube formation assay

The 96-well plates were coated with Matrigel (60 µl/well). HUVECs (4 × 10^4^ cells/well) were resuspended with the indicated CMs and plated onto the Matrigel (356,234, Corning, USA) after solidification for 1 h at 37 °C. Tubes were observed and pictures were taken after 6-hour incubation at 37 °C for tube formation. The formed tubes were analyzed using ImageJ software.

### Cell migration assay

Transwell assays were used to assess the effect of the indicated CMs on HUVEC migration. Briefly, HUVECs were resuspended in a serum-free medium and placed in the upper chamber (4 × 10^4^ cells/well). The lower chamber was then incubated with the indicated CMs for 24 h. Penetrated cells were stained with crystal violet, and images were taken under a microscope to count the cells.

### Enzyme-linked immunosorbent assay (ELISA)

The level of VEGF-A, B, and C in si-SALL4-B, P-SALL4-B, CRISPR/Cas9-KO-SALL4, MGC-803-Thalidomide or engineered exosomes cells’ conditioned medium were determined using an ELISA kit according to the manufacturer’s protocol (Jiangsu Jingmei Biological Technology co., LTD.). The absorbance at 450 nm was measured using a microplate reader (FLX800, USA).

### Immunofluorescence staining

Cells were plated and grown on coverslips overnight. After being fixed in 4% paraformaldehyde, cells were treated with 0.5% Triton X-100 for cell permeabilization. The coverslips were then immersed in blocking solution for 1 h, followed by incubation with anti-GFP (T0005, Affinity Biosciences) overnight. Coverslips were washed twice with PBS. Cells were observed and pictures were acquired by fluorescence microscope (Delta Vision OMX SR; GE Healthcare Bio-Sciences, Piscataway, NJ, USA).

### Chromatin immunoprecipitation assay (ChIP)

According to the manufacturer’s instructions (ab500, Abcam), the chromatin immunoprecipitation assay was performed on MGC-803 cells. Cells were collected in SDS lysis buffer after 10 min of cross-linking with 1% formaldehyde at 37 °C, and the DNA was sonicated to 200 bp fragments. The precleared chromatin was incubated overnight with SALL4 (15H26L3, Thermofisher Scientific) or nonspecific IgG antibodies. Protein A agarose beads were added, and the mixture was incubated at 4 °C for 1 h. Following the reverse of the cross-links, the DNA was isolated and used for PCR. (Additional file 1 Table S3) shows the specific primers for detecting the responsive element in the promoter of VEGF-A, B, and C genes.

### Electrophoretic mobility shift assay (EMSA)

Nuclear extracts were prepared from MGC-803 cells 48 h after transfection with the SALL4-overexpressing plasmid. Nuclear proteins were extracted using a protein extraction kit according to the manufacturer’s instructions (OP-0002, EpiQuick™ Nuclear Extraction Kit 1). A BCA assay (P0011, Beyotime Biotechnology, China) was used to determine nuclear protein concentrations. EMSA kit (E33075, Invitrogen™) was used to investigate the interaction between SALL4 in the nuclear protein extract and the DNA probe. SYBR Green EMSA stain (green) was applied to the gel, followed by SYPRO Ruby EMSA stain (red). The image was documented using a laser-based scanner after each staining (iBright™ 1500 Imagers-Invitrogen™, USA), and the digital images were pseudocolored and overlaid. Yellow bands indicate areas that have been stained with both stains. The probe sequences are listed in (Additional file 1 Table S3).

### Exosomes isolation

Serial centrifugation was used to separate exosomes from the cell culture medium. Cells were cultured in exosome-free FBS for 48 h before the medium was collected. Cell debris is removed by centrifugation at 300 g for 20 min and 2000 g for another 20 min, followed by 30 min of secondary centrifugation at 10,000 g to remove larger vesicles. The medium was then ultracentrifuged at 100,000 g for 3 h, washed with PBS, and centrifuged for another 3 h at 100,000 g. The exosome pellet was resuspended in a suitable volume of PBS. The concentration of exosome suspension was determined using a BCA protein assay kit (P0011, Beyotime Biotechnology, China). Exosomal markers CD63 (ab271286, Abcam), CD9, HSP-70, TSG-101(Cell Signaling Technology, USA), and the ER marker Calnexin (Abcam, UK) were determined.

### Nanoparticle tracking analysis (NTA)

The number and size distribution of isolated exosomes were measured using a ZetaView PMX120 instrument (Particle Metrix, Bavaria, Germany) via a 1-ml syringe. The results were calculated three times on average using the corresponding software, ZetaView 8.02.28.

### Transmission electron microscopy (TEM)

The isolated exosomes were fixed for 5 min in 2% paraformaldehyde before being dropped onto a Formvar copper carbonate grid with glow discharge for 1 min. The cells were then negatively stained for 1 min with 2% uranyl acetate. After drying the sample, the photograph was taken with an electron microscope (Philips, Netherlands) at an acceleration voltage of 80 kV.

### Bioinformatics analysis

The TCGA dataset was used to analyze the relationship between SALL4 expression and survival in STAD patients via https://portal.gdc.cancer.gov and https://www.cbioportal.org/ databases, as well as the Kaplan-Meier Plotter http://kmplot.com/analysis/ database.

### Statistical analysis

Group differences were determined using GraphPad Prism 7.00 software and IBM SPSS 23.0 software. Experimental values are represented as means ± SEM. A two-tailed Student’s t-test (two groups) and a one- and two-way ANOVA test (three or more groups) were conducted as appropriate for differences comparison. The Kaplan-Meier method was employed to analyze patient survival, and Spearman correlation analysis was applied for gene expression correlation analysis. The difference was considered significant as indicated (*P < 0.05, **P < 0.01, and ***P < 0.001).

## Results

### Upregulated SALL4 in STAD promotes tumor progression and indicates a poor prognosis

**Fig. 1:**
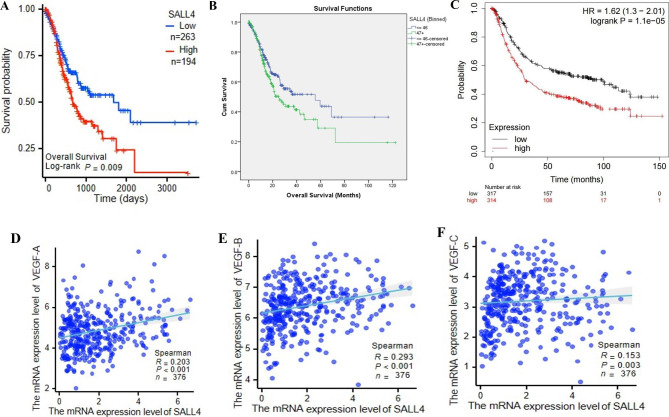
Upregulated SALL4 in STAD promotes tumor progression and indicates a poor prognosis, according to the TCGA database. **A, B** Kaplan-Meier analysis of overall survival in STAD patients based on SALL4 expression; **C** Overall survival analysis for the overall survival of STAD patients via Kaplan-Meier plotter. Data were acquired from the TCGA dataset via https://portal.gdc.cancer.gov, https://www.cbioportal.org/, and http://kmplot.com/analysis/ database and analyzed by IBM SPSS Statistics 23 software. Spearman correlation analysis of SALL4 mRNA expression with the transcripts of angiogenesis-associated genes. **D-F** Scatter plots depicting the significant correlation between SALL4 expression and the mRNA levels of VEGF-A (**D**), VEGF-B (**E**), and VEGF-C (**F**) mRNA expression levels in 367 STAD patients from the TCGA database. Data were acquired from the TCGA dataset via the https://portal.gdc.cancer.gov database and analyzed by IBM SPSS Statistics 23 software. The results are presented as the means ± standard error of mean (SEM). *P < 0.05, **P < 0.01, and ***P < 0.001

To elucidate SALL4 expression signatures in human malignancies, we conducted a pan-cancer analysis of public TCGA datasets at https://portal.gdc.cancer.gov and https://www.cbioportal.org/ database. The overall survival time was significantly shorter in STAD patients with high SALL4 expression than in those with low SALL4 expression (Fig. 1A-C).

### The correlation of SALL4 with the VEGF transcript in the TCGA database

By analyzing a public dataset of 376 STAD patients from TCGA, we found that SALL4 mRNA level was significantly correlated with the transcripts of genes related to angiogenesis, including VEGF-A (R = 0.203, P < 0.001), VEGF-B (R = 0.293, P < 0.001), and VEGF-C (R = 0.153, P = 0.003) (Fig. 1D-F).

### SALL4 regulates VEGF family gene expression

**Fig. 2:**
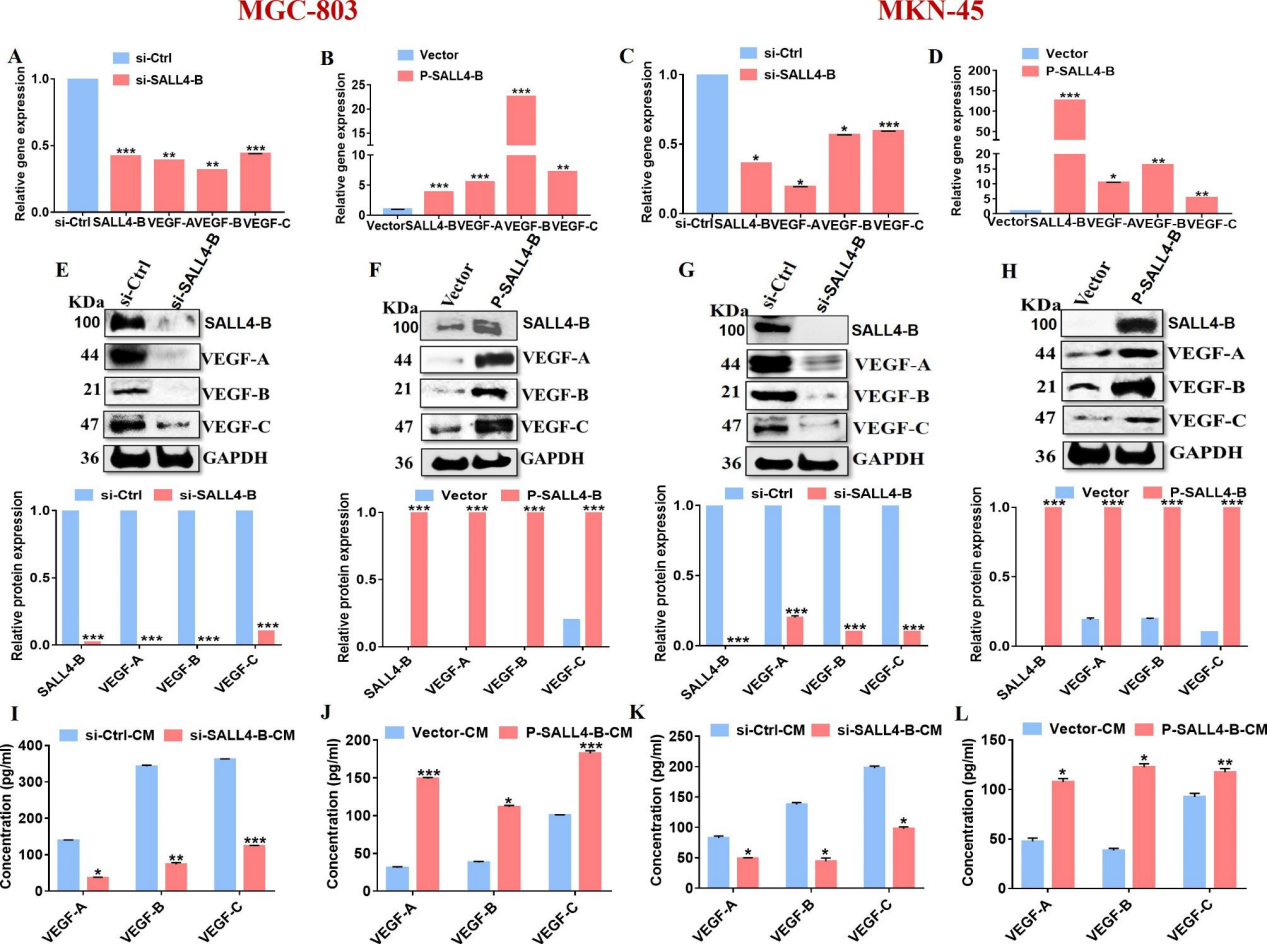
SALL4 regulates VEGF-A, B, and C expression levels. SALL4-B downregulation by siRNA decreases, while upregulation by over-expressing plasmid (P-SALL4-B) increases the expression levels of VEGF-A, B, and C in MKN-45, MGC-803, and HGC-27 cells. **A-D** qRT-PCR analysis of SALL4-B, VEGF-A, B, and C mRNA expression levels in MKN-45, MGC-803, and HGC-27 cells. **E-H** Western blot analyses for protein levels of SALL4-B, VEGF-A, VEGF-B, and VEGF-C in MKN-45, MGC-803, and HGC-27. GAPDH was used as a loading control. **I-L** The expression level of VEGF-A, B, and C in the CM from MKN-45, MGC-803, and HGC-27-si-NC/si-SALL4-B and Vector/ P-SALL4-B cells were detected by ELISA assay. The results are presented as the means ± standard error of mean (SEM). *P < 0.05, **P < 0.01, and ***P < 0.001

To verify the regulatory effects of SALL4 on the expression of angiogenic factors VEGF-A, B, and C, we first transfected MKN-45, and MGC-803 cells with SALL4-B or control siRNAs and MKN-45, MGC-803, and HGC-27 cells with SALL4 over-expressing plasmid (P-SALL4-B) or empty vector. QRT-PCR results showed that the mRNA levels of SALL4-B were markedly downregulated in SALL4-B siRNA-transfected cells while upregulated in P-SALL4-B-transfected cells compared to control siRNA or empty vector-transfected cells (Fig. 2A-D). Additionally, qRT-PCR results revealed that the knockdown of SALL4-B decreased while SALL4-B overexpression increased the mRNA levels of VEGF-A, B, and C (Fig. 2A-D). Western blot results further demonstrated that SALL4-B downregulation decreased, while over-expression increased VEGF-A, B, and C protein expression (Fig. 2E-H). Furthermore, we analyzed the levels of VEGF-A, B, and C in the conditioned medium (CM) from gastric cancer cells transfected with si-SALL4-B or P-SALL4-B and found that the concentrations of VEGF-A, B, and C decreased in SALL4-B siRNA-transfected cells while increased in P-SALL4-B-transfected cells (Fig. 2I-L). These results suggest that SALL4 positively regulates the expression of VEGF family genes.

### SALL4 regulates cancer angiogenesisin vitro

**Fig. 3:**
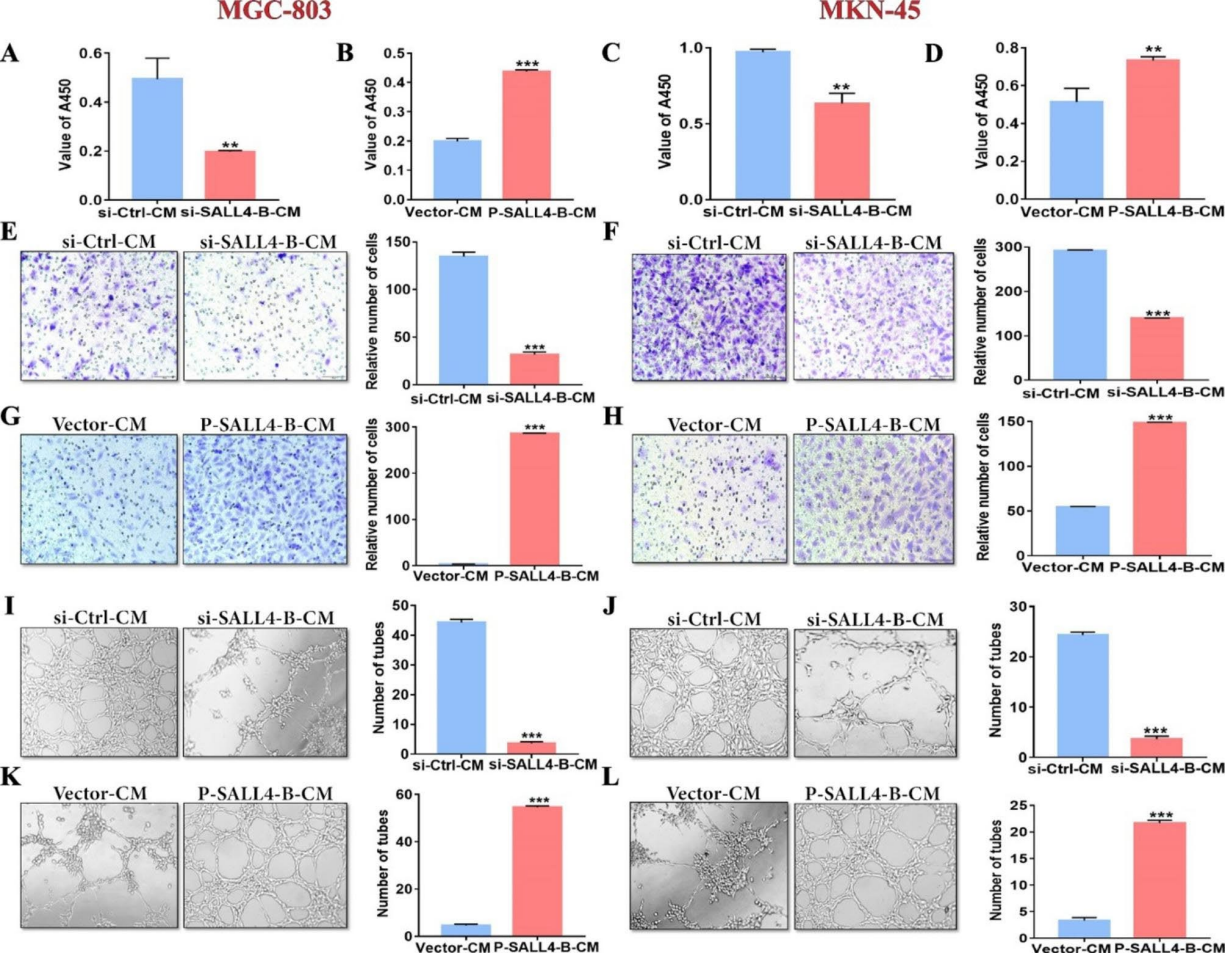
SALL4 regulates angiogenesisin vitro. **A-D** CCK-8 assays were used to assess cell proliferation after a 48-hour treatment. **E-H** Transwell migration assays were performed on HUVECs treated with CM from si-Ctrl/si-SALL4-B or Vector/P-SALL4-B gastric cancer cells. **I-L** Tube formation assays in si-Ctrl/si-SALL4-B or Vector/P-SALL4-B CM-treated HUVECs. The results are presented as the means ± standard error of mean (SEM). **P < 0.01, and ***P < 0.001

Given the potential role of SALL4 in regulating VEGF gene expression, we sought to investigate whether SALL4 modulates angiogenesis in gastric cancer. In response to treatment with CM collected from gastric cancer cells, we observed that HUVECs cultured in CM from si-SALL4-B cells showed a significant reduction in viable cell number, while those cultured in CM from P-SALL4-B cells showed a significantly increased number of viable cells compared to control group as measured by CCK-8 assay (Fig. 3A-D). We found that the migration of HUVECs was markedly alleviated after treatment with CMs from si-SALL4-B cells, while increased after treatment with CMs from P-SALL4-B cells compared to the control group (Fig. 3E-H). To substantiate the relevance of SALL4 to angiogenesis in gastric cancer, HUVECs were treated with CM from si-SALL4-B or P-SALL4-B cells and assayed for tube formation. We observed that as expected, HUVECs exhibited diminished tube formation ability following treatment with CM from si-SALL4-B cells, while increased tube formation ability following stimulation with CMs from P-SALL4-B cells compared to the control group (Fig. 3I-L). Taken together, these in vitro observations validate the potent role of SALL4 in gastric cancer angiogenesis.

### SALL4 binds to VEGF family gene promoters and activates their expression by inducing epigenetic modifications

**Fig. 4:**
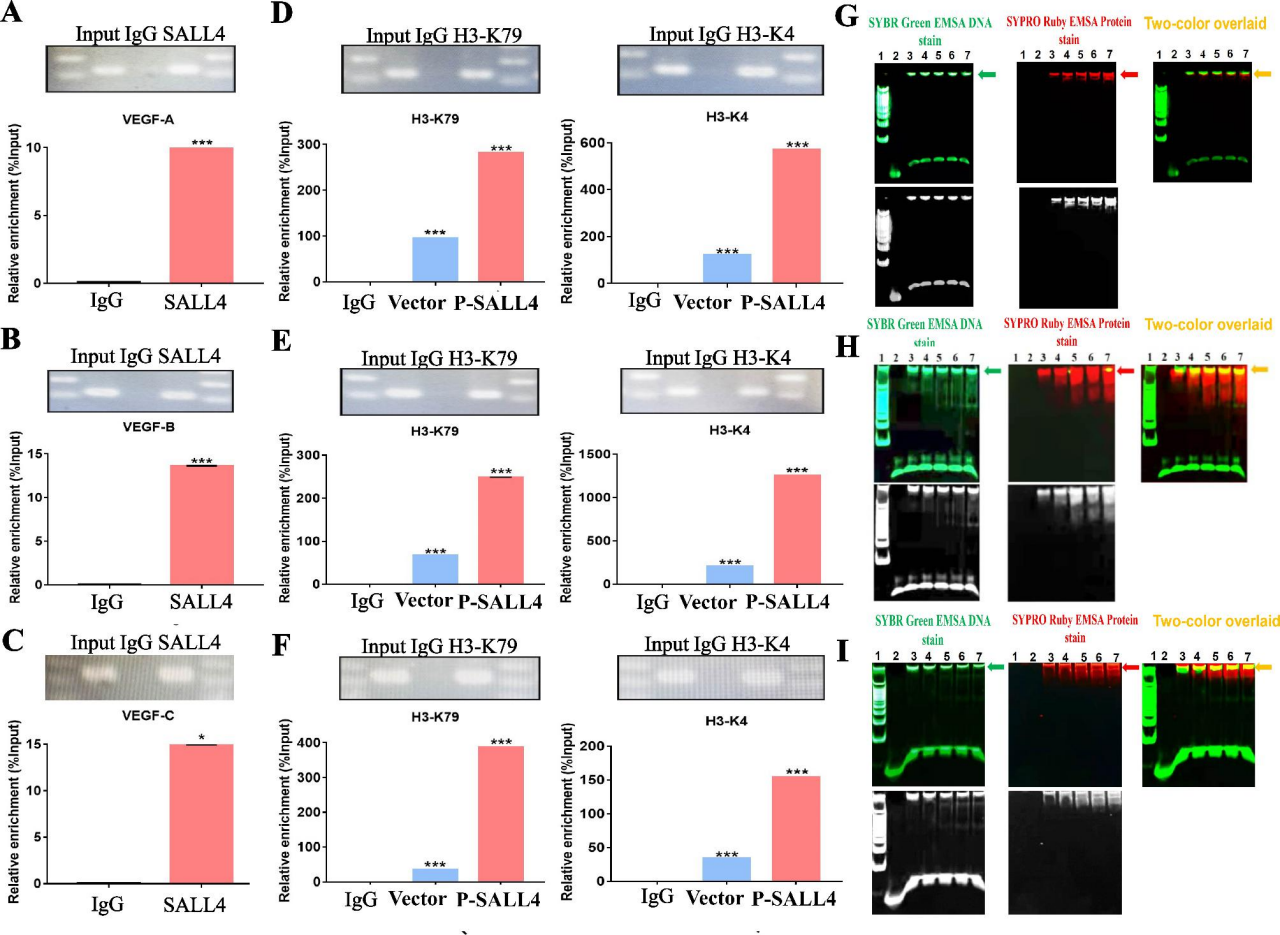
SALL4 binds to VEGF-A, B, and C gene promoter and activate their expression. **A-F** ChIP was used to examine the association of SALL4, Di-methylation (H3-K79), or Tri-methylation (H3-K4) with the VEGF-A, B, or C promoters in MGC-803 cells. Briefly, qRT-PCR was used to amplify SALL4, Di-methylation (H3-K79), or Tri-methylation (H3-K4) immunoprecipitated DNA using specific primers. As indicated, total input was used as a positive control. **G-I**. EMSA assay was used to determine the binding of VEGF-A, B, or C probes to nuclear extract protein (P-SALL4). To this end, Lane 1 contains DNA markers. Lane 2 contains only VEGF-A, B, or C probe (100 ng). Lanes 3–7: 100 ng aliquots of VEGF-A, B, or C probe with increasing amounts of nuclear extract protein (P-SALL4) (3,5,7,9,11 ug). SYBR Green EMSA stain was used to stain the gel in panel A (Green). Panel B shows the same gel stained with SYPRO Ruby EMSA stain (Red). The arrow points to the VEGF-A, B, or C probe-nuclear extract protein (P-SALL4) complex, which is stained in both panels (Yellow). (Additional file 2) shows the sequences of the putative SALL4-binding site. The results are presented as the means ± standard error of mean (SEM). *P < 0.05, *** *P* < 0.001

To identify that SALL4 could directly bind to the VEGF-A, B, and C promoter, we performed a chromatin immunoprecipitation (ChIP) assay. The putative SALL4-binding site exhibited a significant enrichment after immunoprecipitation with an anti-SALL4 antibody. No band was evident after immunoprecipitation with negative control IgG antibody (Fig. 4A-C). Electrophoretic mobility shift assays (EMSA) were performed to confirm whether SALL4 is directly bound to the VEGF-A, B, and C gene promoters. According to the predicted transcription factor-binding site, probes were designed and synthesized. EMSA results showed that SALL4 is directly bound to the promoter regions of VEGF-A, B, and C genes (Fig. 4G-I). Collectively, these results suggest that SALL4 has a vital role in gastric cancer angiogenesis through directly binding to VEGF gene promoters. We also used a ChIP assay on MGC-803 gastric cancer cells that had been transfected with P-SALL4 and then performed immunoprecipitation by using antibodies specific for histone H3-K79 di-methylation and H3-K4 tri-methylation. Consistent with the binding of SALL4 to VEGF family gene promoters in the MGC-803 gastric cancer cells transfected with P-SALL4, H3–K79 di-methylation and H3–K4 trimethylation were detected and increased in these cells as compared with control vector group (Fig. 4D-F).

### Knockout of SALL4 by CRISPR/Cas9 inhibits gastric angiogenesisin vitro

**Fig. 5:**
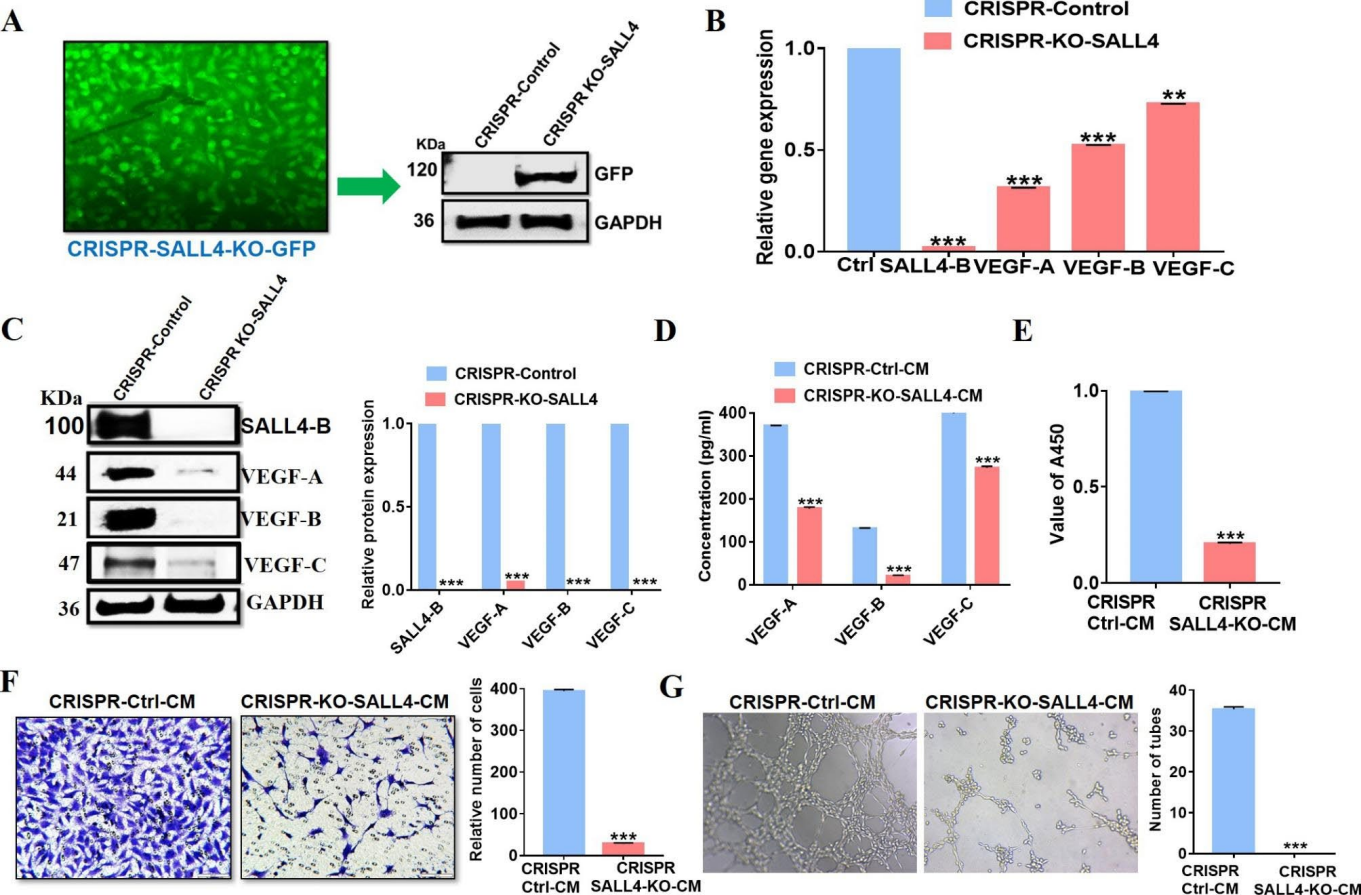
Knockout of SALL4 by CRISPR/Cas9 inhibits the pro-angiogenic effect of gastric cancer cellsin vitro. **A** Immunofluorescence and western blot analysis were used for the confirmation of the CRISPR/Cas9 knockout experiment. **B** The expression levels of SALL4-B, VEGF-A, B, and C mRNA were determined by qRT-PCR in gastric cancer cells with SALL4 knockout by CRISPR/Cas9. **C** The expression levels of SALL4-B, VEGF-A, B, and C proteins were determined by western blot in gastric cancer cells with SALL4 knockout by CRISPR/Cas9. GAPDH was used as a loading control. **D** The expression level of VEGF-A, B, and C in the CM from control and SALL4 knockout cells were detected by ELISA assay. HUVECs were treated with a serum-free medium or CMs from SALL4 knockout gastric cancer cells. **E** After treatment for 48 h, CCK-8 assays were performed to evaluate cell proliferation. **F** Transwell migration assay was performed to determine the migration of HUVECs with CM treatment. **G** Tube formation in HUVECs treated with indicated CMs. The results are presented as the means ± standard error of mean (SEM). ***P < 0.001

To further verify the effect of SALL4 expression on gastric cancer angiogenesis, we first established a gastric cancer cell line with SALL4 knockout by using CRISPR/Cas9 technology (MGC-803-SALL4 KO) (Fig. 5A). We found that MGC-803-SALL4 KO cells also presented low levels of VEGF-A, VEGF-B, and VEGF-C as confirmed by qRT-PCR (Fig. 5B) and western blot (Fig. 5C). Also, we analyzed the level of VEGF-A, B, and C in CM from MGC-803-SALL4 KO cells by ELISA and found that their concentrations were all decreased compared to the control group (Fig. 5D). Then, CMs were gathered from MGC-803-SALL4 KO cells for further proliferation, migration, and tube formation assays with HUVECs. We found that CM from MGC-803-SALL4 KO cells had a lower ability to promote the proliferation (Fig. 5E), migration (Fig. 5F), and tube formation (Fig. 5G) of HUVECs than that from control cells. Hence, SALL4 knockout greatly attenuates gastric cancer angiogenesis in vitro.

### Thalidomide decreases SALL4 expression and inhibits angiogenesis

**Fig. 6:**
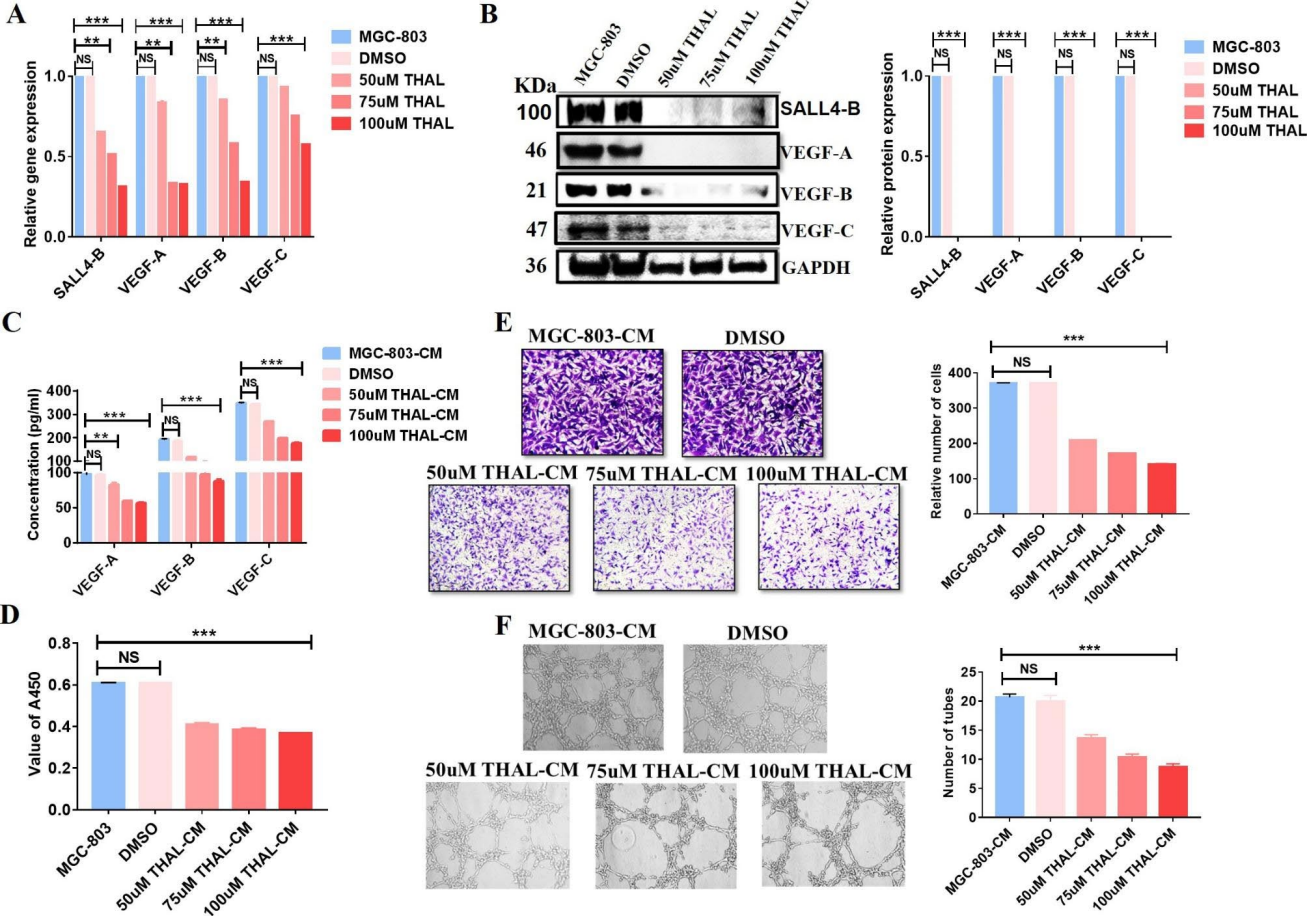
Thalidomide decreases SALL4 expression and inhibits angiogenesis. **A** SALL4-B, VEGF-A, B, and C expression levels were measured in MGC-803 cells treated with different concentrations of Thalidomide by using real-time qRT-PCR. **B** SALL4-B, VEGF-A, B, and C protein expression levels were determined by using western blots in MGC-803 cells treated with different concentrations of Thalidomide. **C** ELISA assay was used to measure VEGF-A, B, and C expression levels in a conditioned medium (CM) from MGC-803 cells. **D** CCK-8 assay was used to assess HUVEC cell proliferation after treatment with CM from MGC-803 cells. **E** Transwell assay was used to examine the effects of CM from MGC-803 cells on the migration of HUVEC cells. **F** Tube formation of HUVECs after treatment with CM from MGC-803 cells. The results are presented as the means ± standard error of mean (SEM) **P < 0.01, and ***P < 0.001

Thalidomide has been regarded as a potent inhibitor of SALL4 in numerous studies [21]. We also applied thalidomide in our study to inhibit the activation of SALL4 and tested whether this treatment will affect gastric cancer angiogenesis through modulating VEGF gene expression. First, qRT-PCR results showed that thalidomide could significantly decrease the mRNA levels of SALL4-B and VEGF family genes in MGC-803 cells in a concentration-dependent manner (Fig. 6A). Western blot results further confirmed this effect (Fig. 6B). Additionally, we analyzed the level of VEGF-A, B, and C in CM from MGC-803 cells treated with different concentrations of Thalidomide and found that the concentrations of VEGF-A, B, and C decreased after treatment (Fig. 6C). We next investigated the potential role of thalidomide in the promotion of endothelial cell proliferation, migration, and tube formation in vitro. We found that CM from thalidomide-treated MGC-803 cells resulted in a significant decrease in the proliferation (Fig. 6D) and migration (Fig. 6E) of HUVECs compared to the control group. Furthermore, our data revealed that CM from thalidomide-treated MGC-803 cells led to reduced ability of tube formation in terms of decreased branch points and tube length HUVECs compared to the control group (Fig. 6F). These data suggest that thalidomide attenuates gastric cancer angiogenesis by suppressing SALL4 and the downstream VEGF signaling.

### Exosomes deliver thalidomide drug and si-SALL4-B to decrease SALL4 to inhibit angiogenesis

**Fig. 7:**
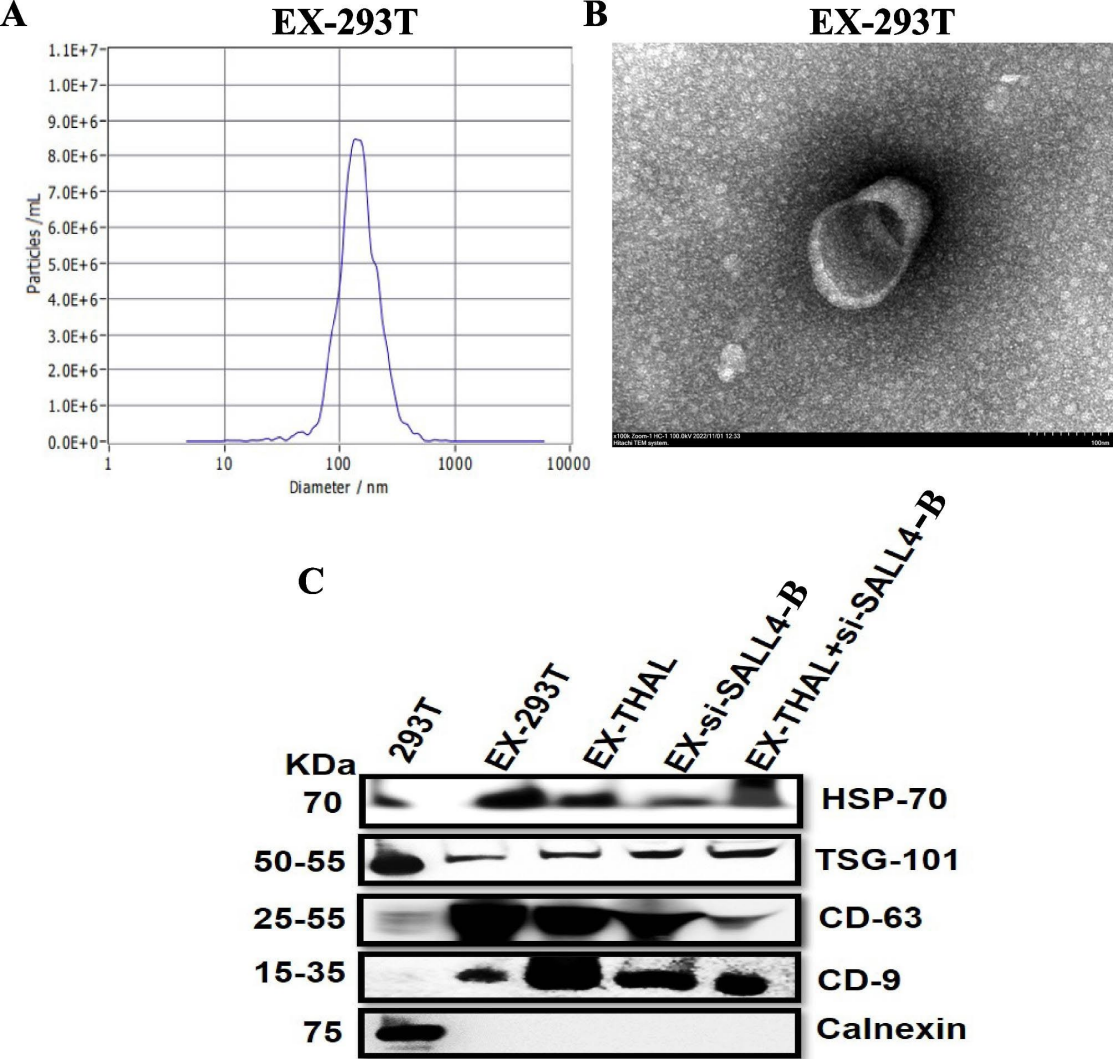
Characterization of exosomes isolated from 293-T cells. **A** NTA analysis was used to determine the size range of exosomes isolated from 293-T cells. **B** Exosomes isolated from 293-T cells are shown in a TEM image. **C** Exosomal markers including CD9, CD63, TSG-101, HSP-70, and ER marker Calnexin were analyzed by Western blot

**Fig. 8:**
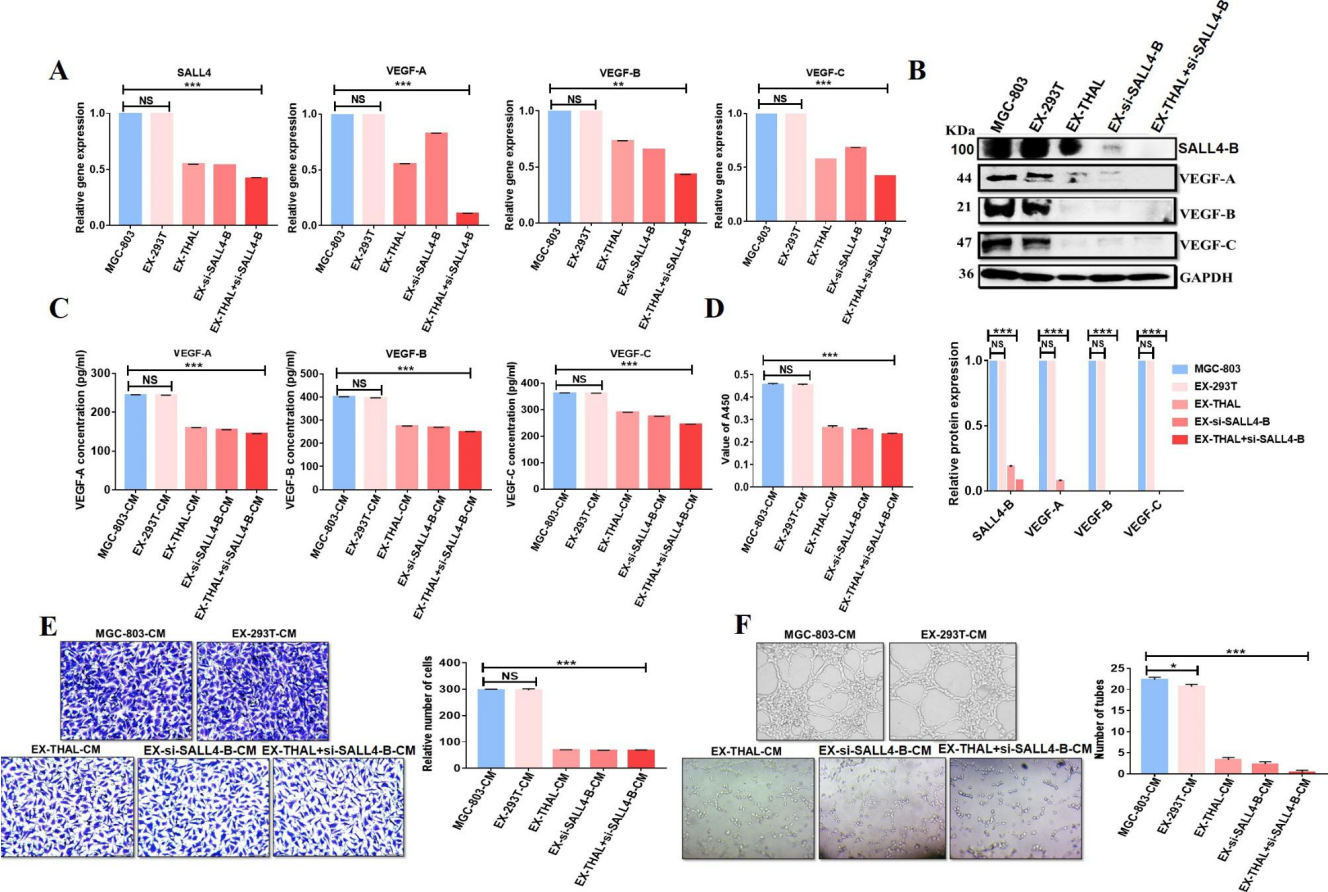
Exosomes deliver thalidomide and si-SALL4-B to target SALL4 for inhibiting angiogenesis. **A** SALL4-B, VEGF-A, B, and C expression levels were detected in MGC-803 cells treated with engineered exosomes (EX-293T, EX-THAL, EX-si-SALL4-B, and EX-THAL + si-SALL4-B) by using real-time RT-PCR. **B** SALL4-B, VEGF-A, B, and C expression was detected by using a western blot in MGC-803 cells treated with engineered exosomes. **C** ELISA assay was used to measure VEGF-A, B, and C expression levels in the conditioned medium from MGC-803 cells treated with engineered exosomes. HUVEC cells were incubated with the conditioned medium from engineered exosomes-treated MGC-803 cells. The proliferation, migration, and angiogenic abilities of HUVEC cells were analyzed by using the CCK-8 assay (**D**), transwell assay (**E**), and tube formation assay (**F**). The results are presented as the means ± standard error of mean (SEM). **P < 0.01, *** P < 0.001

Considering the critical role of SALL4 in cancer angiogenesis by regulating VEGF, we wanted to target SALL4 for gastric cancer therapy by using an exosome-mediated drug delivery approach. We transfected 293T cells with SALL4-targeting siRNA (EX-si-SALL4-B) and/or incubated them with thalidomide (EX-THAL). Exosomes were isolated by ultracentrifugation from the cell culture supernatants of si-SALL4-B-293T and/or thalidomide-293T cells. TEM and Nanoparticle tracking analysis (NTA) were used to evaluate the morphology and size of the exosomes. EX-293T, EX-THAL, EX-si-SALL4-B, and EX-THAL + si-SALL4-B had typical saucer-like bilayer membrane structures with ~ 145 nm diameters (Fig. 7A, B). Western blot analyses confirmed the expression of exosome markers such as CD63, CD9, HSP-70, TSG-101, and ER marker Calnexin was identified (Fig. 7C). MGC-803 cells were treated with different engineered exosomes (EX-THAL, EX-si-SALL4-B, and EX-THAL + si-SALL4-B) in vitro to evaluate their therapeutic potentials. The results showed that MGC-803 cells treated with engineered exosomes decreased SALL4-B, VEGF-A, VEGF-B, and VEGF-C mRNA and protein levels as measured by qRT-PCR and western blot, respectively (Fig. 8A, B). Also, we used ELISA to examine the levels of VEGF-A, B, and C in CM from MGC-803 cells treated with engineered exosomes and showed that the concentrations of VEGF-A, B, and C were lower than that in the control group (Fig. 8C). We observed that the ability of CM from MGC-803 cells to promote HUVEC cell proliferation (Fig. 8D), migration (Fig. 8E), and tube formation (Fig. 8F) were remarkably inhibited after treatment with engineered exosomes. According to these findings, the engineered exosomes loaded with si-SALL4-B and/or thalidomide were efficiently taken up by MGC-803 cells to inhibit their pro-angiogenic activity in vitro.

## Discussion

Here, we investigated the potential role of SALL4 in gastric cancer angiogenesis. We found that SALL4 is frequently overexpressed in STAD patients and positively correlated with tumor progression. Also, SALL4-B downregulation suppressed, whereas overexpression increased the proliferation, migration, and tube formation of HUVECs. Furthermore, SALL4-B expression was positively correlated with that of VEGF family genes in gastric cancer cells. More importantly, SALL4 bound to the promoter regions of VEGF family genes and initiated histone modifications to activate their expression, suggesting that SALL4 plays an important role in gastric cancer progression by regulating VEGF.

SALL4 (sal-like 4) is a transcription factor that is abundantly expressed in fetal tissues [22]. Restored SALL4 expression has been found in various tumors and linked to cancer progression. VEGF is the primary regulator of pro-angiogenic factors, inducing endothelial cell sprouting and proliferation [23]. The secretion of VEGF by tumor cells contributes to neovascularization, which in turn helps cancer development and progression [24, 25]. Recent studies indicated that SALL4 downregulation inhibits endothelial cell proliferation, cell cycle progression, migration, and tube formation in HUVEC [26]. Additionally, VHL mutation-mediated SALL4 overexpression promoted clear cell renal cell carcinoma (ccRCC) cell proliferation, colony formation, cell cycle progression, migration, invasion, tumorigenicity, and tumor vascularization through modulating Akt/GSK-3β axis and VEGF-A expression [27]. In accordance with previous studies, we revealed that SALL4 knockdown by si-SALL4-B or knockout by CRISPR/Csa9 decreased, while SALL4 overexpression by P-SALL4-B increased the levels of VEGF-A, B, and C, which indicates that SALL4/VEGF axis may be a common regulatory mechanism for cancer angiogenesis.

Several studies have revealed that SALL4 could bind to different gene promoters and activate their expressions. For example, SALL4 promoted EMT and antineoplastic drug resistance by regulating c-Myc [15]. SALL4 induced epithelial-mesenchymal transition and promoted tumor progression in breast cancer by directly binding to the vimentin promoter [28]. Also, SALL4 bound to the TGF-β1 promoter and promoted gastric cancer metastasis by upregulating TGF-β1 and activating the SMAD signaling pathway [14]. In addition, SALL4 bound to the CD44 promoter region and activated its transcription, and CD44 overexpression reversed the inhibition of gastric cancer cell proliferation, migration, and invasion caused by SALL4 knockdown [29]. The oncogene Bmi-1 is a direct target gene of SALL4, and the SALL4/Bmi-1 network plays a key role in leukemogenesis [30]. Furthermore, SALL4 bound specifically to the HOXA9 promoter and promoted human myeloid leukemogenesis [31]. In agreement with the previous findings, our results showed that SALL4 could bind to the (-601 to -711), (-690 to -822), (-852 to -1080) regions of the promoter of VEGF-A, B, and C genes, respectively, and activate their expression in gastric cancer cells. Histone methylation at H3-K4 and H3-K79 sites is associated with the SALL4 binding region of the Bmi-1 promoter and increased in the presence of SALL4 [30]. The epigenetic activation markers H3-K4 and H3-K79 are also found to be enriched in the same HOXA9-I region bound by SALL4 [31]. By physically interacting with DOT1-like histone H3-K79 methyltransferase (DOT1l) and LSD1/KDM1A, SALL4 enhances the levels of H3K79me2/3 and H3K4me3 at target gene promoters and thus activates transcription [32]. In another study, it was confirmed that treatment with a hypomethylating agent led to demethylation of the CpG region and up-regulation of SALL4 expression [33]. Here, our findings indicate that the SALL4 binding regions of the VEGF-A, B, and C promoters are hypermethylated at H3–K4 and H3–K79 histones in the presence of SALL4.

Thalidomide was developed in 1954 and was first used to treat respiratory infections in 1967. When reports from various countries revealed that the drug was teratogenic, it was withdrawn. It was known that the teratogenic effects of thalidomide are caused by the protein cereblon, which is found in both embryonic and adult tissues. Cereblon is necessary for normal morphogenesis [34]. Thalidomide has wide anti-cancer and antiangiogenic properties [35, 36]. Thalidomide can control biological features that are crucial in the context of tumor development and secondary spread. It is capable of inhibiting angiogenesis and cell proliferation, as well as the promotion of apoptosis. In previous studies, it was discovered that thalidomide causes CRBN-dependent degradation of SALL4 [21, 37]. Thalidomide’s antiangiogenic activity is related to its inhibitory action on VEGF secretion and microvessel formation by human endothelial cells [38]. In advanced esophageal cancer, thalidomide in combination with TP (pacilitaxel plus cisplatin) chemotherapy inhibits tumor angiogenesis by lowering serum VEGF levels [39]. Thalidomide also inhibits VEGF-A expression in colorectal cancer cells in a dose and time-dependent manner [40]. Transcatheter arterial chemoembolization (TACE) in combination with thalidomide-mediated adjuvant treatment has demonstrated a promising clinical outcome in hepatocellular carcinoma (HCC) patients by lowering VEGF levels [41]. We found that gastric cancer cells treated with different concentrations of thalidomide expressed lower levels of VEGF-A, B, and C at both mRNA and protein levels. Furthermore, the CM from thalidomide-treated gastric cancer cells displayed an impaired effect to promote the proliferation, migration, and tube formation of HUVECs, indicating that thalidomide may target SALL4 to inhibit VEGF signaling.

Recent advances in drug delivery biomaterials have enabled remarkable progress in disease treatment [42, 43]. This discovery enabled the biomaterial-based drug delivery strategies to be a novel method for cancer inhibition. Exosomes have been considered as promising drug delivery vehicles that can deliver chemotherapeutics, proteins, or genes against tumors and have unique advantages such as nanosized, biodegradability, and tumor-homing function [44–46]. Exosomes also have improved stability, enhanced endocytosis, and lower toxicity in vivo [44]. This evidence suggests that exosomes may be a novel nanosized drug delivery system for cancer treatment. Recent studies indicate that cisplatin encapsulated in M1 macrophage exosomes effectively inhibits lung cancer cell proliferation and induces apoptosis. The simultaneous delivery of miR-21i and 5-FU via exosomes significantly increases cytotoxicity in 5-FU-resistant colon cancer cells [47]. A functionalized macrophage exosome-based nano-drug delivery system loaded with Panobinostat and PPM1D siRNA effectively kills Pontine Gliomas (DIPG) tumor cells in vitro and achieves significant tumor growth inhibition and prolonged survival time in orthotopic DIPG-bearing mice [48]. In addition, mesenchymal stem cell (MSC)-derived exosomes can transfer miR-15a to HCC cells to inhibit proliferation, migration, and invasion by negatively regulating SALL4 [49]. Also, SALL4 could bind to the promoter of miR-146a-5p and directly influence its expression in exosomes. In DEN/CCL4-induced HCC mice, blocking the interaction between SALL4 and miR-146a-5p lowered inhibitory receptor expression on T cells, reversed T cell exhaustion, and delayed HCC progression [50]. Based on the above studies, it is plausible that an exosome-based nano-drug delivery system loaded with thalidomide and SALL4-B siRNA inhibited SALL4-B and VEGF family gene expression in gastric cancer cells. After treatment with engineered exosomes, the CM from gastric cancer cells had a remarkably decreased ability to promote HUVEC cell proliferation, migration, and tube formation, indicating that this strategy may represent a new regimen for cancer therapy by suppressing SALL4/VEGF pathway via exosome-mediated drug delivery.

In conclusion, our study suggests that SALL4 plays a critical role in gastric cancer angiogenesis by modulating VEGF expression, and targeting SALL4 may be an effective strategy for anti-angiogenic therapy of gastric cancer.

## Conclusion

**Fig. 9:**
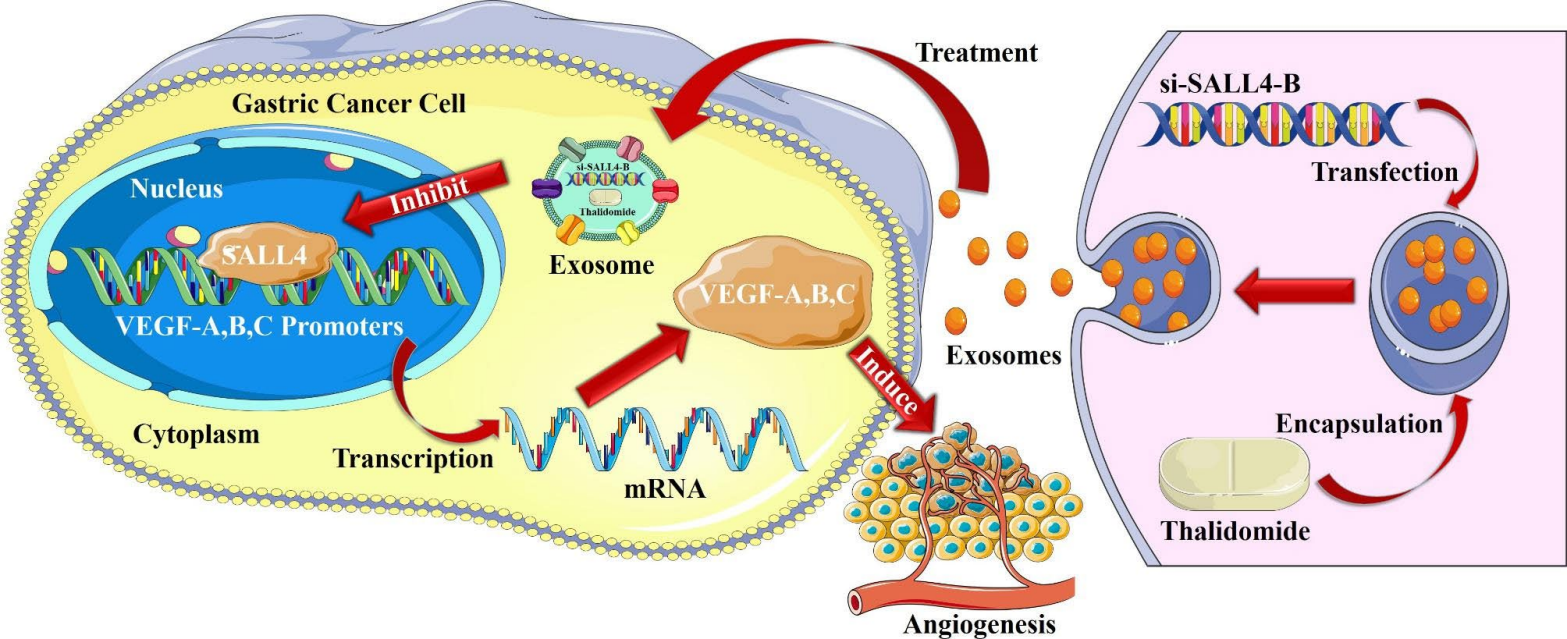
Proposed model of SALL4’s role in gastric cancer angiogenesis. SALL4 functions as a transcription factor that binds to VEGF-A, B, and C promoters and activates their expression. Exosomal delivery of si-SALL4-B and thalidomide results in targeted inhibition of SALL4-B and VEGF-A, B, and C expression, leading to suppression of gastric cancer angiogenesis

Collectively, these findings demonstrate a strong role for SALL4 in gastric cancer angiogenesis through modulating the VEGF family and establishing a synergistic treatment via the exosomes-nano carrier system as a promising strategy for gastric cancer treatment (Fig. 9).

## Electronic supplementary material

Below is the link to the electronic supplementary material.

**Additional file 1**: **Table S1**. Sequences of siRNA. **Table S2**. Sequences of PCR primers for target gene detection. **Table S3**. Sequences of ChIP-PCR primers and EMSA probes for target gene detection.

**Additional file 2**: Sequences of the putative SALL4-binding site.

## Data Availability

All data generated or analyzed during this study are included in this published article.

## Abbreviations

ChIP: Chromatin immunoprecipitation
CM: Conditioned medium
EMSA: Electrophoretic mobility shift assay
EX: Exosome
HUVEC: Human umbilical vein endothelial cell
SALL4: Spalt-like protein 4
STAD: Stomach adenocarcinoma
THAL: Thalidomide
VEGF: Vascular endothelial growth factor
