# Supplementary material for: SALL4 promotes angiogenesis in gastric cancer by regulating VEGF expression and targeting SALL4/VEGF pathway inhibits cancer progression
Source: Cancer Cell Int. 2023 Jul 31;23:149. doi: 10.1186/s12935-023-02985-9 (PMC10388482; doi:10.1186/s12935-023-02985-9)
Supplement: Supplementary file 1 — Additional file 1: Table S1. Sequences of siRNA. Table S2. Sequences of PCR primers for target gene detection. Table S3. Sequences of ChIP-PCR primers and EMSA probes for target gene detection. [file 12935_2023_2985_MOESM1_ESM.pdf]

Supplementary Materials

Additional file 1 Table S1 Sequences of siRNA

siRNA	Target sequence
**Si-Ctrl**	**5'-UUCUCCGAACGUGUCACGUTT-3'**<br>**5'-ACGUGACACGUUCGGAGAATT-3'**
**Si-SALL4-B**	**5'-GUCUCUGGAUGCCUUGAAATT-3'**<br>**5'-UUUCAAGGCAUCCAGAGACTT-3'**

Additional file 1 Table S2 Sequences of PCR primers for target gene detection

Gene	Sequence	Size (bp)	T_M (°C)
**β-actin**	**F: 5′-CACGAAACTACCTTCAACTCC-3′**<br>**R: 5′-CATACTCCTGCTTGCTGATC-3**	**265**	**60**
**SALL4-B**	**F: 5′-TCGATGGCCAACTTCCTTC-3′**<br>**R: 5′-GAGCGGACTCACACTGGAGA-3′**	**142**	**60**
**VEGF-A**	**F: 5'-AAAGGGTGGAGGTGACTG-3'**<br>**R: 5'-GACATAAATGACCGAGGC-3'**	**134**	**60**
**VEGF-B**	**F: 5'-AGTGCTGTGAAGCCAGACA-3'**<br>**R: 5'-GGAGTGGGATGGGTGATG-3'**	**119**	**60**
**VEGF-C**	**F: 5'-ATGTGTGTCCGTCTACAGATGT-3'**<br>**R: 5'-GGAAGTGTGATTGGCAAAACTGA-3'**	**160**	**60**

Additional file 1 Table S3 Sequences of ChIP-PCR primers and EMSA probes for target gene detection

Gene	Sequence	Size (bp)	T_M (°C)
**ChIP-PCR primers**			
**VEGF-A**	**F: 5’- GAAGCAACTCCAGTCCCAAATA-3’**<br>**R: 5’- GCTCTGGCTAAAGAGGGAATG-3’**	**111**	**60**
**VEGF-B**	**F: 5’-AAGGGTTAGACTGAGGTTCCTA-3’**<br>**R: 5’-CCTGCAATGGGTCACAATTAAC-3’**	**133**	**60**
**VEGF-C**	**F: 5’- AACAGCCACACAGAATGGAAGG-3’**<br>**R: 5’- TTACAGAGGACCACGCTAAGGG-3’**	**229**	**60**
**EMSA probes**			
**VEGF-A**	**5'- AGCTGTTTGGGAGGTCAGAAATAGGG -3'**<br>**5'- CCCTATTTCTGACCTCCCAAACAGCT -3'**	**26**	**68**
**VEGF-B**	**5'- ACTTCAGGGACAGCAGGAATTGGA -3'**<br>**5'- TCCAATTCCTGCTGTCCCTGAAGT -3'**	**24**	**68**
**VEGF-C**	**5'-ACCAGATAAGAAAGTCTCTTCTTCCGG T-3'**<br>**5'-A CCGGAAGAAGAGACTTTCTTATCTGGT-3**	**28**	**67**

Additional file 2 Sequences of the putative SALL4-binding site.

A. **NC_000006.12:43768209-43770308 Homo sapiens chromosome 6, GRCh38.p13 (VEGF-A)**

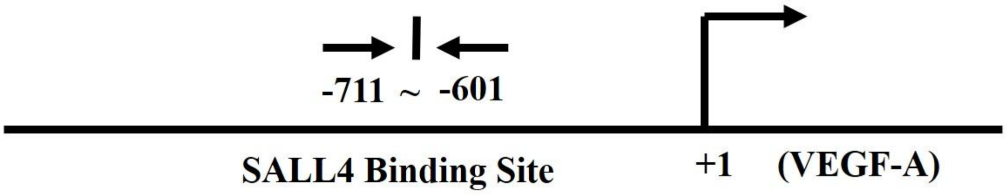

(-711)-GAAGCAACTCCAGTCCCAAATATGTAGCTGTTTGGGAGGTCAGAAATAGGGGGTCCAGGAG
CAAACTCCCCCCACCCCCTTTCCAAAGCCCATTCCCTCTTTAGCCAGAGC -(-601)

B. **NC_000011.10:64232584-64234683 Homo sapiens chromosome 11, GRCh38.p13 (VEGF-B)**

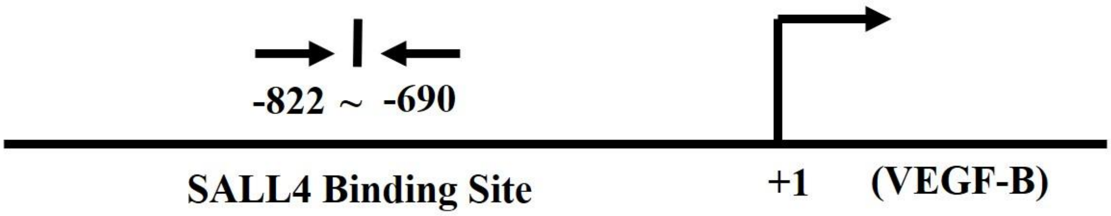

(-822)- AAGGGTTAGACTGAGGTTCCTAACCCTGTGTAAGGGTAAAAAGCTGTGAGGCCCCTAGA
GTGGGGTGGAAGGGGGCAGGACTTCAGGGACAGCAGGAATTGGATTCCCAGGGTTAATT
GTGACCCATTGCAGG -(-690)

C. **<u>NC_000004.12:c176794922-176792823 Homo sapiens chromosome 4, GRCh38.p13 （VEGF-C）</u>**

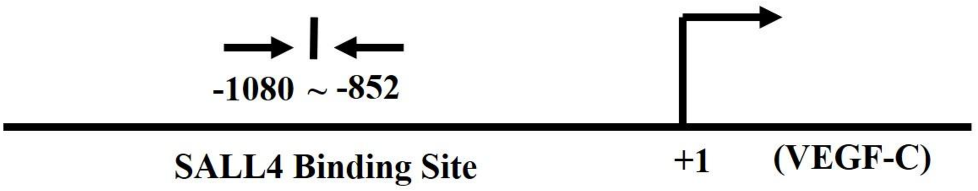

(-1080)- AACAGCCACACAGAATGGAAGGTTCCCTGTCCTTTGAGATATTTAAGCCTTCAAGTAAA
TTATGGGTGAGGAGTTTCAAATCTAGAGTTGAACCAGATAAGAAAGTCTCTTCTTCCGG
TAAGATATTATGGACCTATAACATCTGTGTACTTAAAAGTAGATTGGGAGTGAAAGGCA
GACTTTTGATGTTCTGTACACTGTTGAAACCCCTTAGCGTGGTCCTCTGTAA -(-852)
