# Supplementary material for: SALL4 promotes angiogenesis in gastric cancer by regulating VEGF expression and targeting SALL4/VEGF pathway inhibits cancer progression
Source: Cancer Cell Int. 2023 Jul 31;23:149. doi: 10.1186/s12935-023-02985-9 (PMC10388482; doi:10.1186/s12935-023-02985-9)

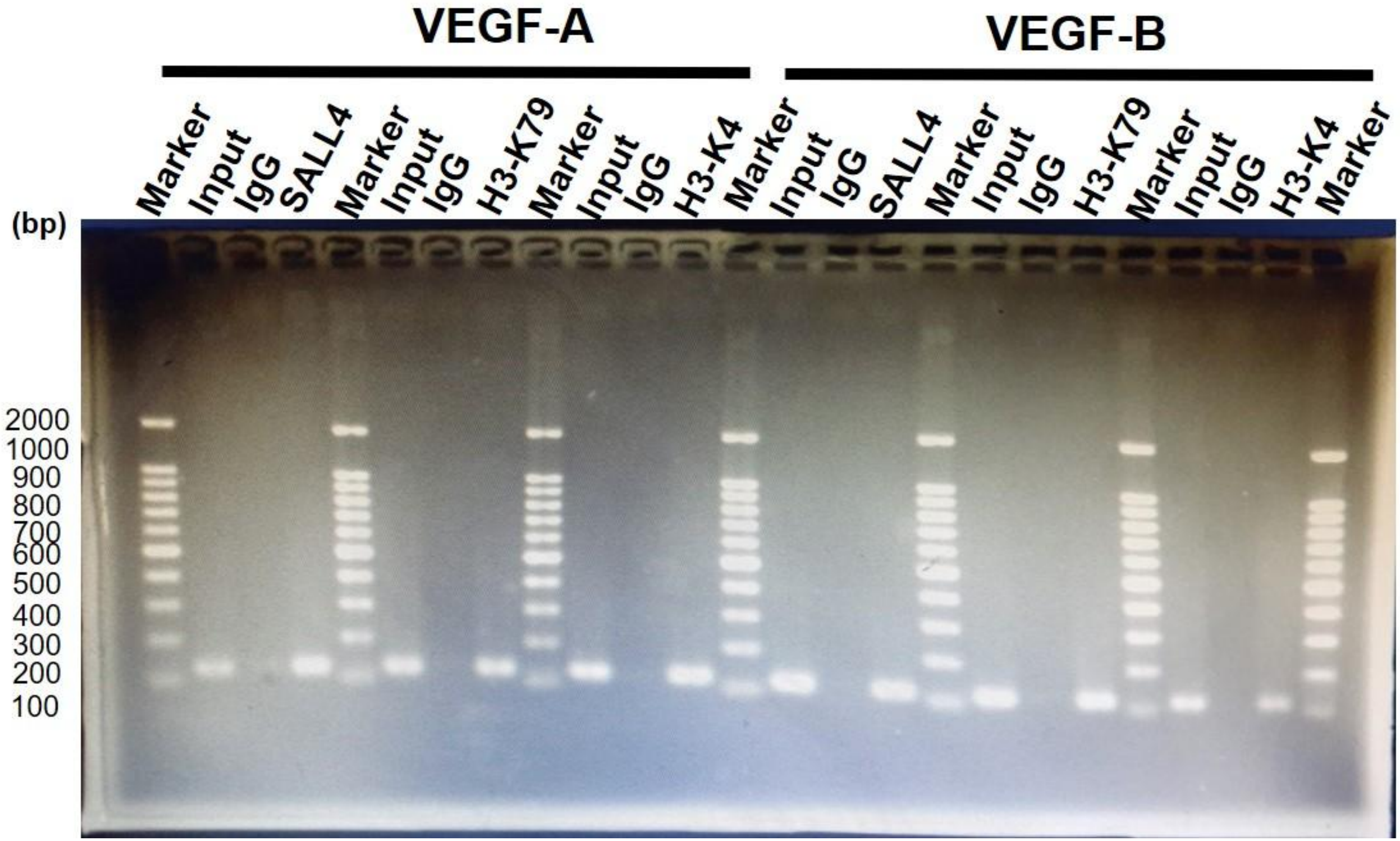

VEGF-A
VEGF-B
Marker
Input
IgG
SALL4
Marker
Input
IgG
H3-K79
Marker
Input
IgG
H3-K4
Marker
Input
IgG
SALL4
Marker
Input
IgG
H3-K79
Marker
Input
IgG
H3-K4
Marker
(bp)
2000
1000
900
800
700
600
500
400
300
200
100

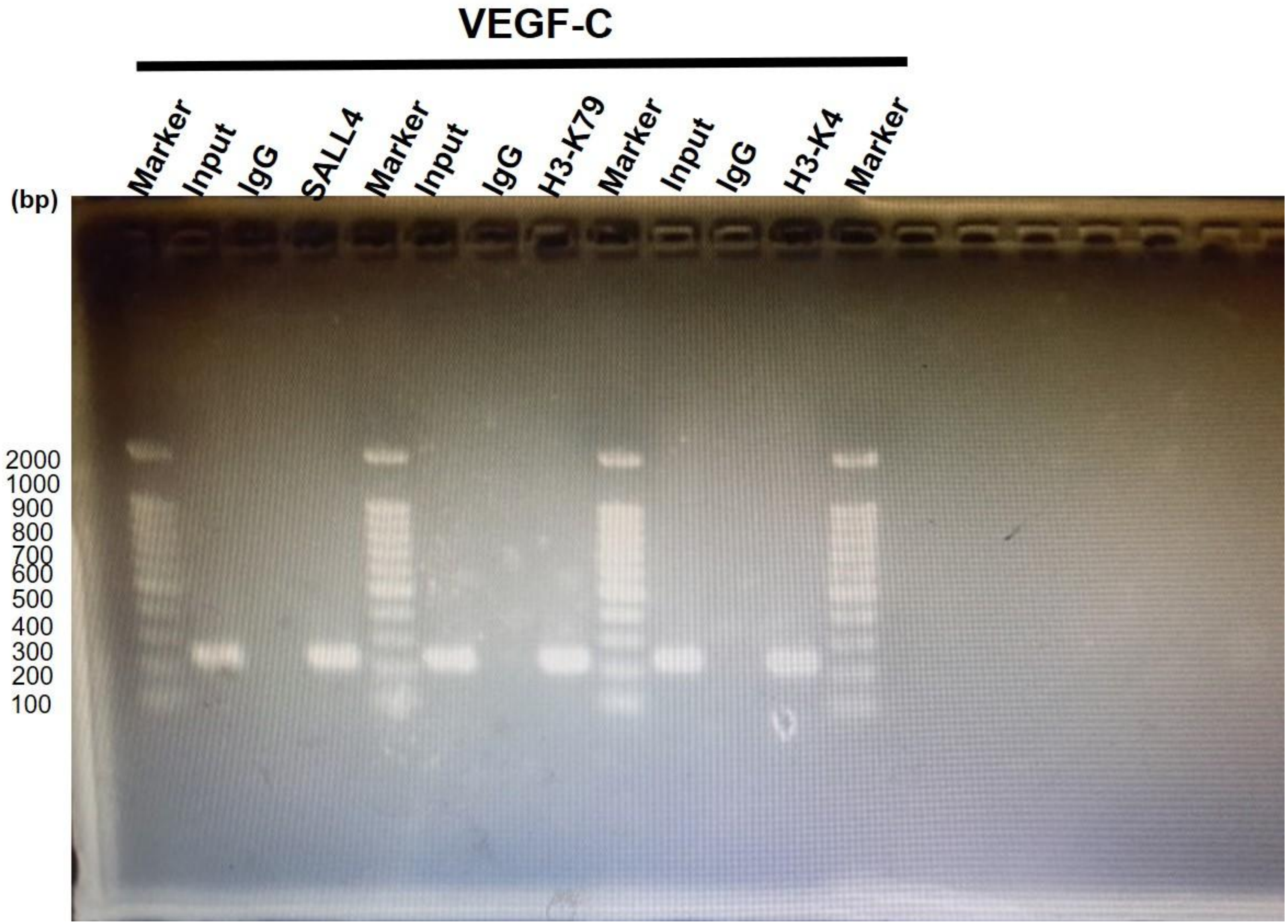

VEGF-C
Marker
Input
IgG
SALL4
Marker
Input
IgG
H3-K79
Marker
Input
IgG
H3-K4
Marker
(bp)
2000
1000
900
800
700
600
500
400
300
200
100

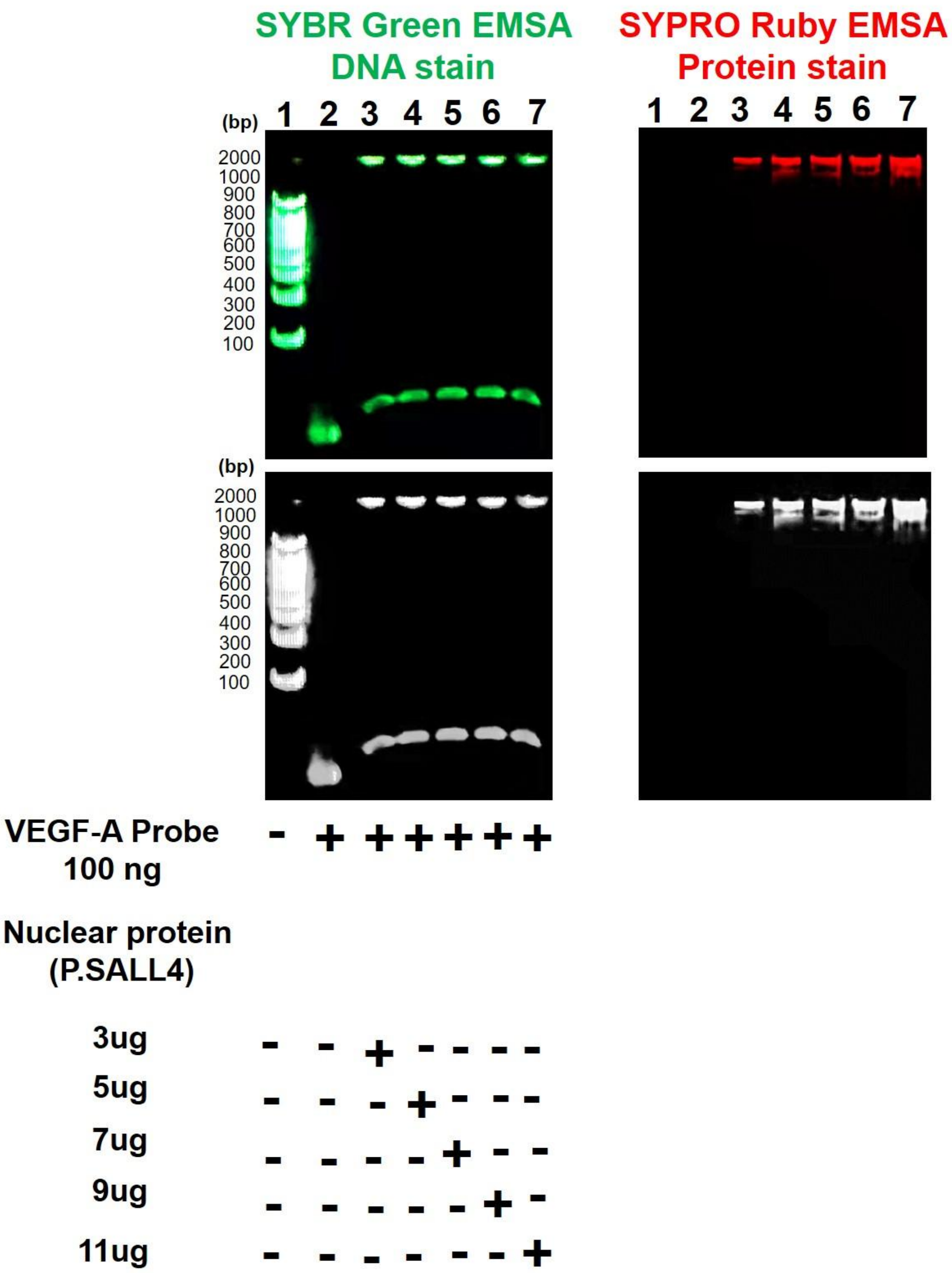
SYBR Green EMSA
DNA stain
SYPRO Ruby EMSA
Protein stain
(bp)
1 2 3 4 5 6 7
2000
1000
900
800
700
600
500
400
300
200
100
1 2 3 4 5 6 7
(bp)
2000
1000
900
800
700
600
500
400
300
200
100
VEGF-A Probe 100 ng - + + + + + +
Nuclear protein (P.SALL4)
3ug - - + - - - -
5ug - - - + - - -
7ug - - - - + - -
9ug - - - - - + -
11ug - - - - - - +

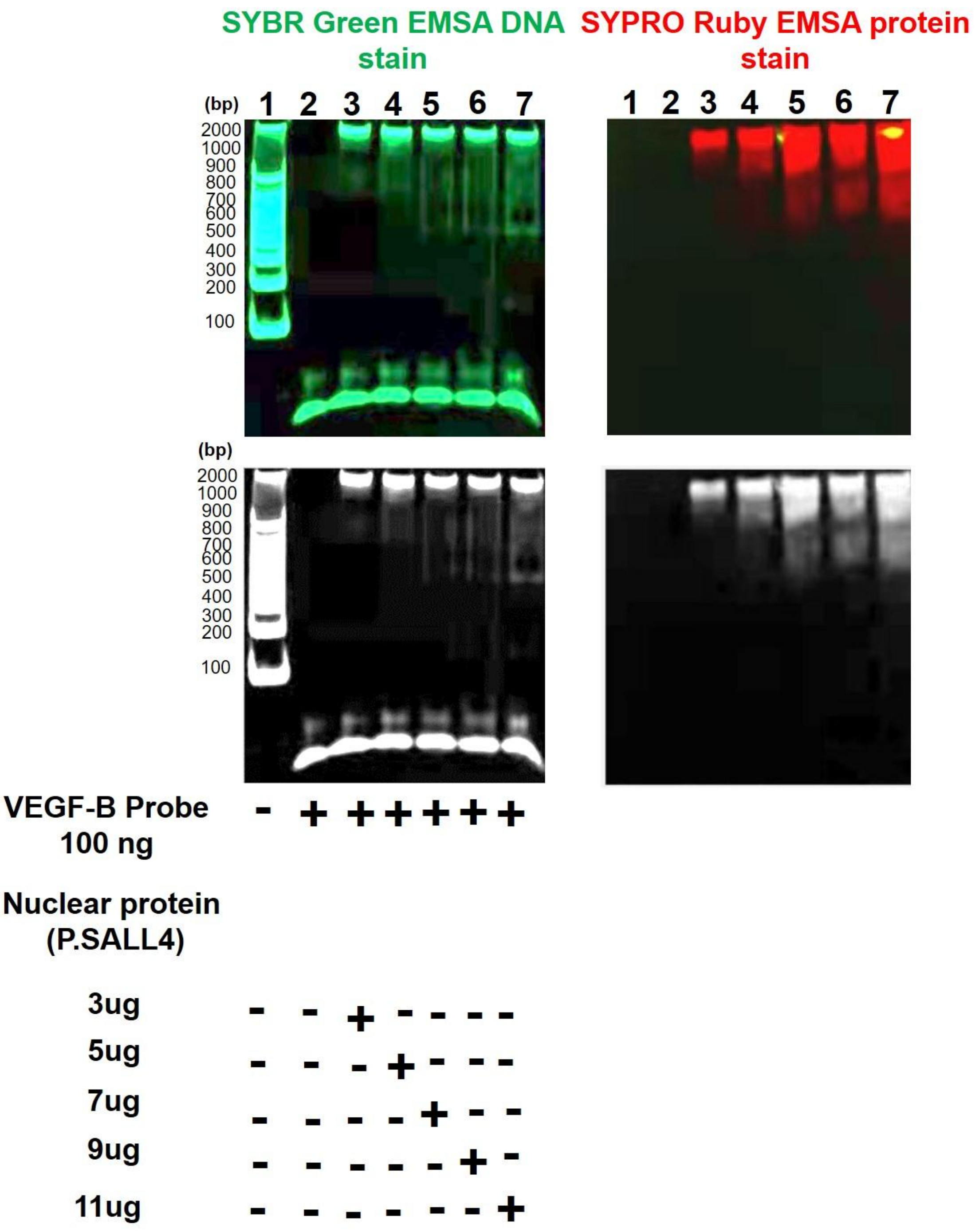
SYBR Green EMSA DNA stain
SYPRO Ruby EMSA protein stain
(bp)
1 2 3 4 5 6 7
2000
1000
900
800
700
600
500
400
300
200
100
1 2 3 4 5 6 7
(bp)
2000
1000
900
800
700
600
500
400
300
200
100
VEGF-B Probe 100 ng - + + + + + +
Nuclear protein (P.SALL4)
3ug - - + - - - -
5ug - - - + - - -
7ug - - - - + - -
9ug - - - - - + -
11ug - - - - - - +

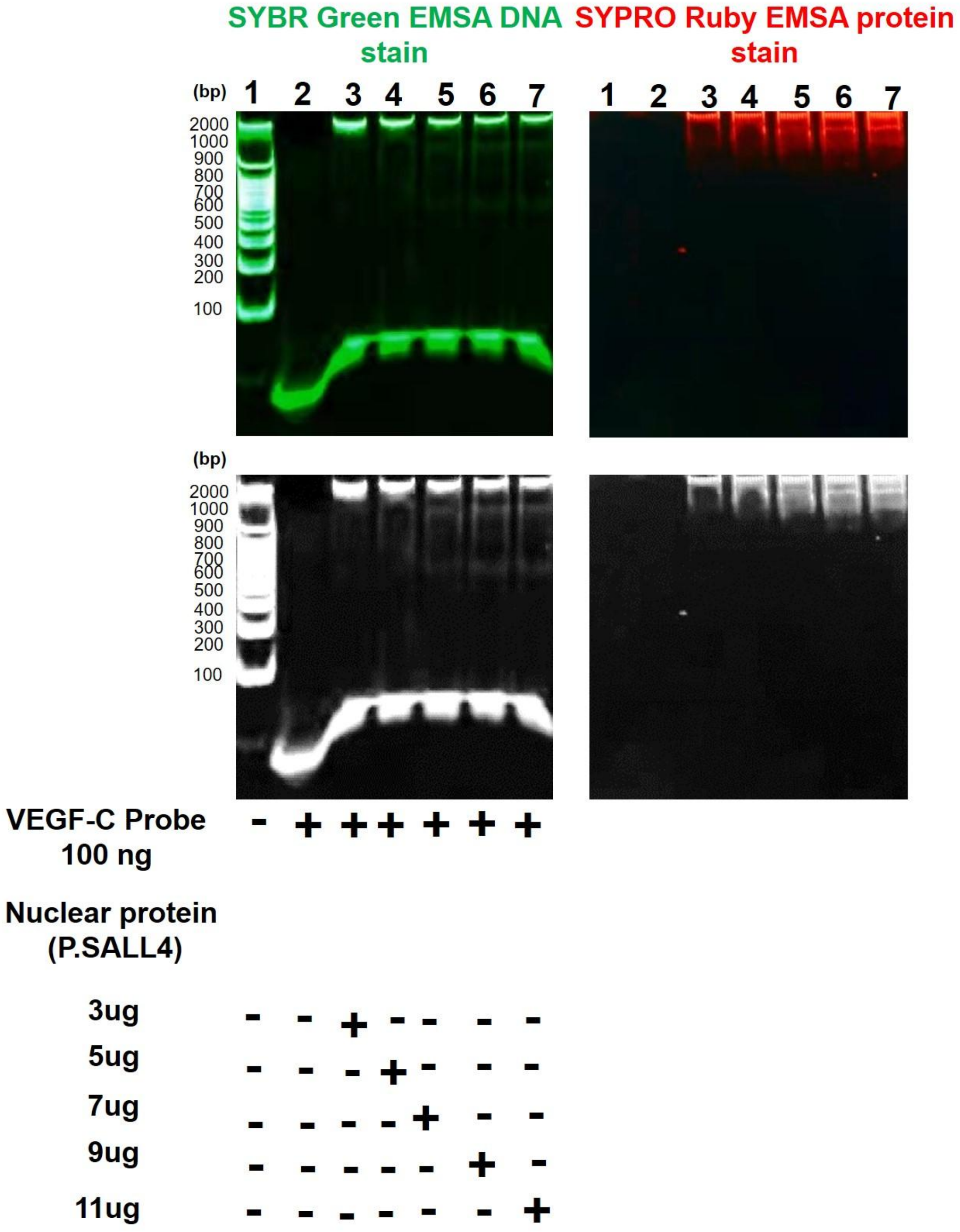
SYBR Green EMSA DNA stain
SYPRO Ruby EMSA protein stain
(bp)
1 2 3 4 5 6 7
2000
1000
900
800
700
600
500
400
300
200
100
VEGF-C Probe 100 ng
- + + + + + +
Nuclear protein (P.SALL4)
3ug - - + - - - -
5ug - - - + - - -
7ug - - - - + - -
9ug - - - - - + -
11ug - - - - - - +

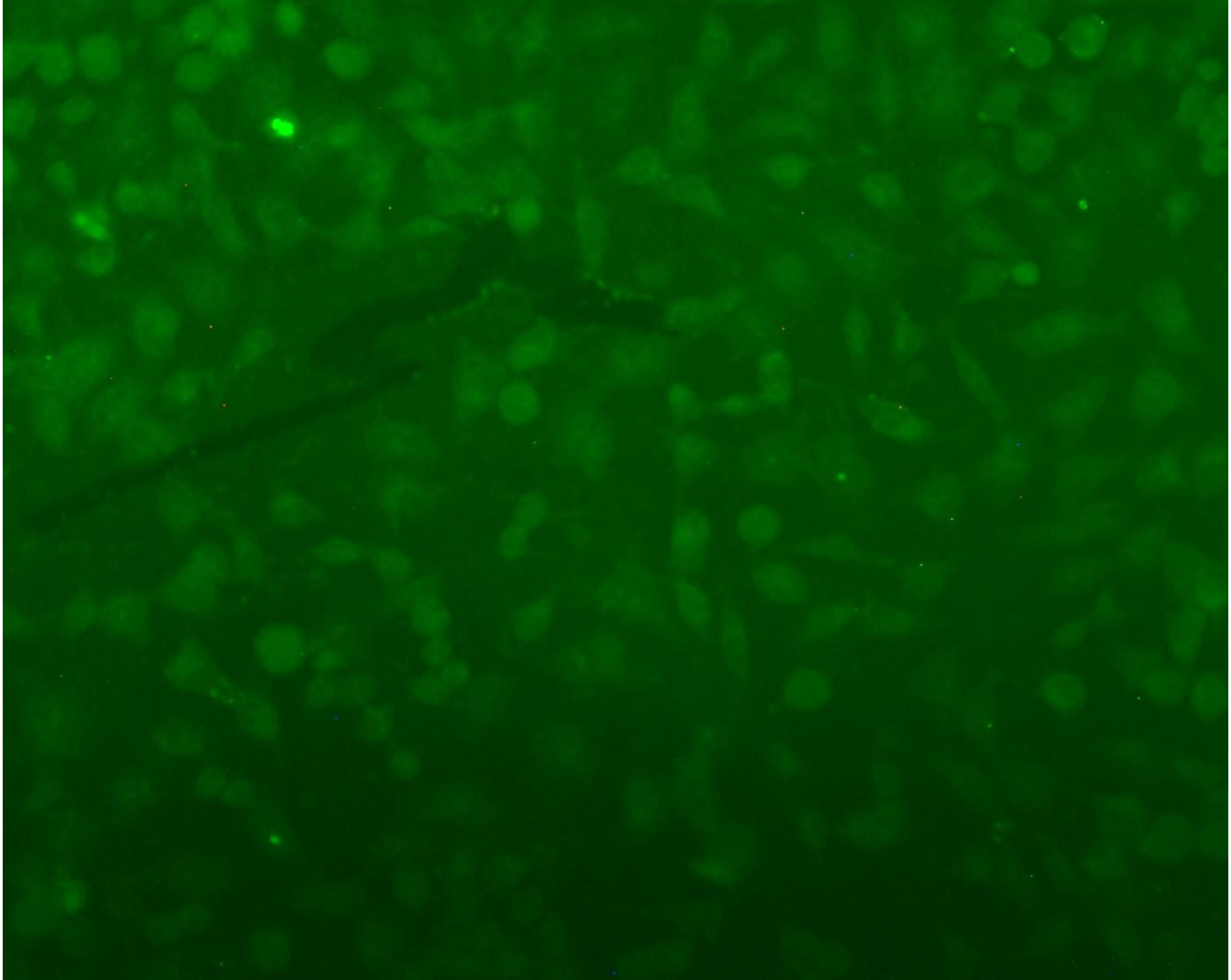

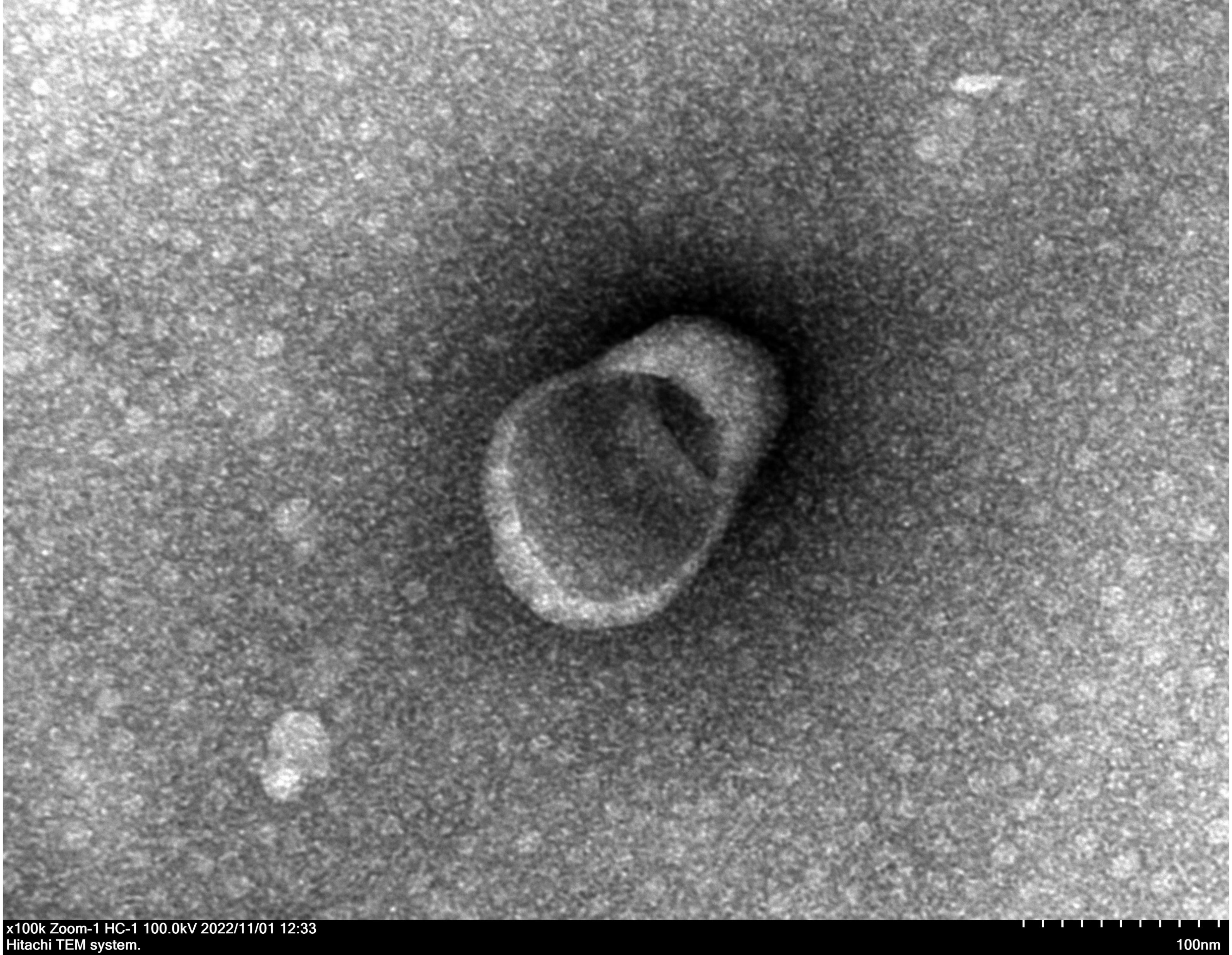
x100k Zoom-1 HC-1 100.0kV 2022/11/01 12:33
Hitachi TEM system.
100nm

Electrophoresis & Brownian Motion
Video Analysis
Laser Scattering Microscopy

PARTICLEMETRIX
www.particle-metrix.de

Operator (Report): ZetaView
Video Operator: ZetaView

Sample Parameters
Sample Name: gp2
Comment: Sample Remarks0:
Sample Remarks1:
Sample Remarks2:
Electrolyte:
Temperature: 27.93 °C sensed
pH 7.0 entered
Conductivity: 25.34 µS/cm sensed

Instrument Parameters
Laser Wavelength: 488 nm
Filter Wavelength: Scatter

Measurement Parameters
Cell S/N: ZNTA

Measurement Mode: Size Distribution 2 Cycles
11 Positions

Result (sizes in nm)

	Number	Concentration	Volume
Median (X50)	142.5	142.5	241.3
Span	68.7	68.7	165.9

Concentration: 2.1E+8 Particles / mL
Dilution Factor: 3000
Original Concentration: 6.4E+11 Particles / mL

Quality
Average Counted Particles per Frame: 612
Number of Traced Particles: 5167

Analysis Parameters
Max Area: 1000, Min Area: 5, Min Brightness: 20

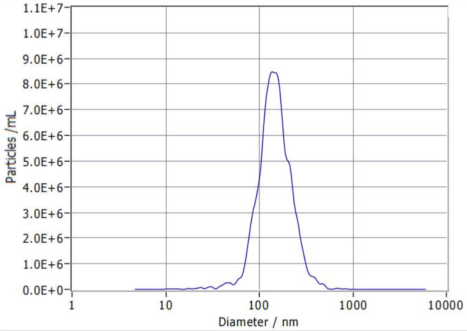

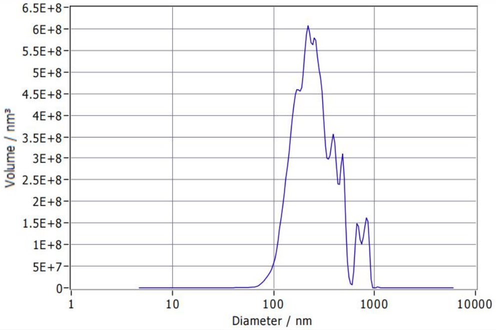

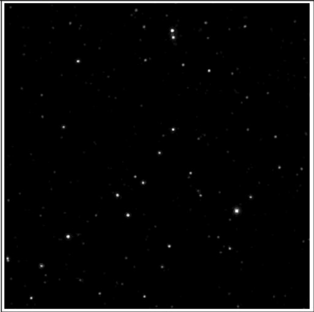

Peak Analysis (Concentration)

Diameter / nm	Particles/mL	FWHM / nm	Percentage
140.6	8.5E+6	123.7	100.0

X Values (all sizes are given in nm)

	Number	Concentration	Volume
X10	87.5	87.5	139.0
X50	142.5	142.5	241.3
X90	236.4	236.4	481.5
Span	1.0	1.0	1.4
Mean	158.0	158.0	293.2
StdDev	68.7	68.7	165.9

Comment

(Signature)

Analyzed Video: Z:\fatma\20220531\20220531_0003_gp2_size_488.avi

ZetaVIEW S/N 20-524, Software ZetaView (version 8.05.12 SP1), Camera 0.713 mum/px

Experiment: 2022-05-31 10:37, Report: 2022-05-31 10:39

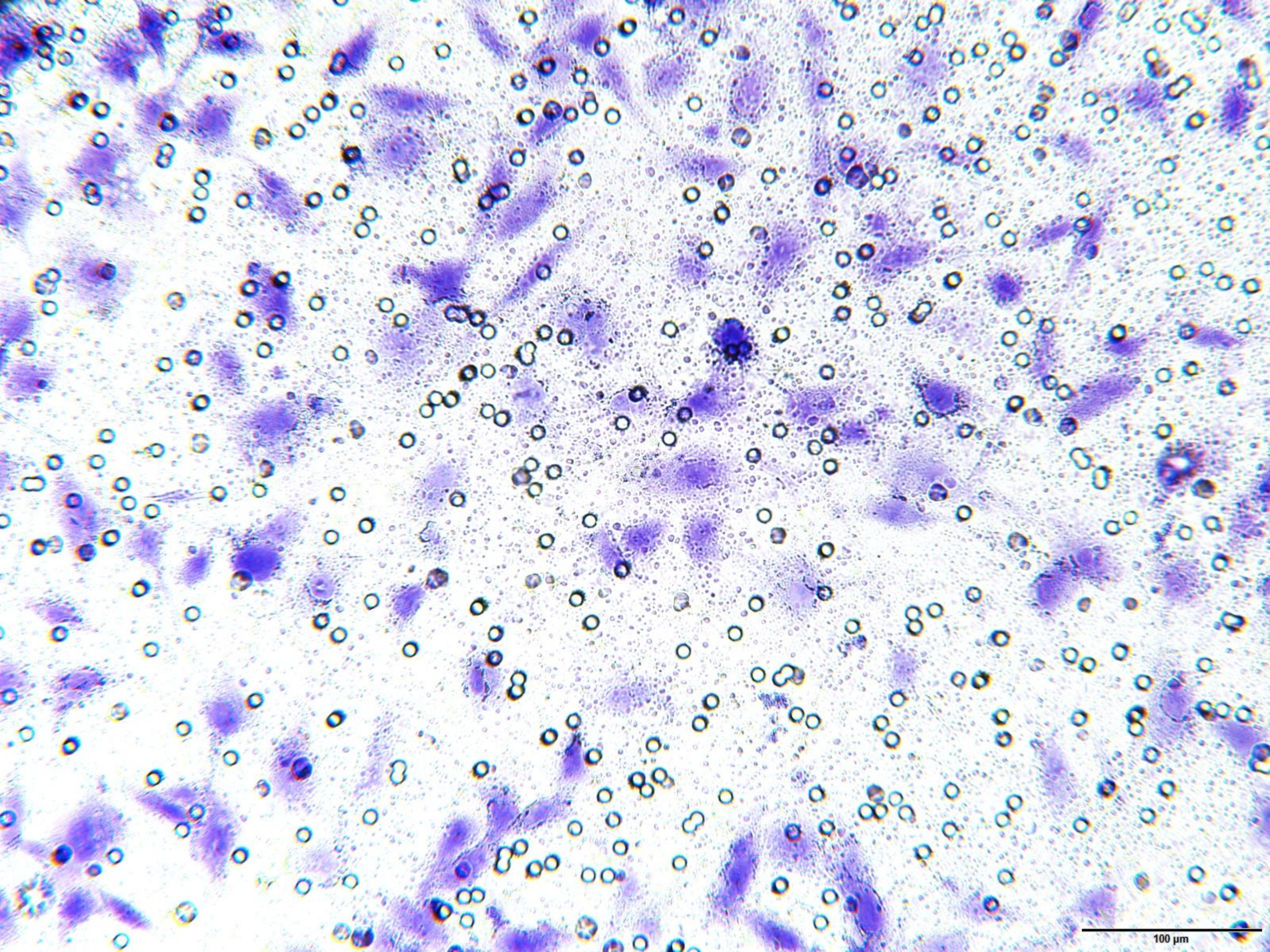
100 µm

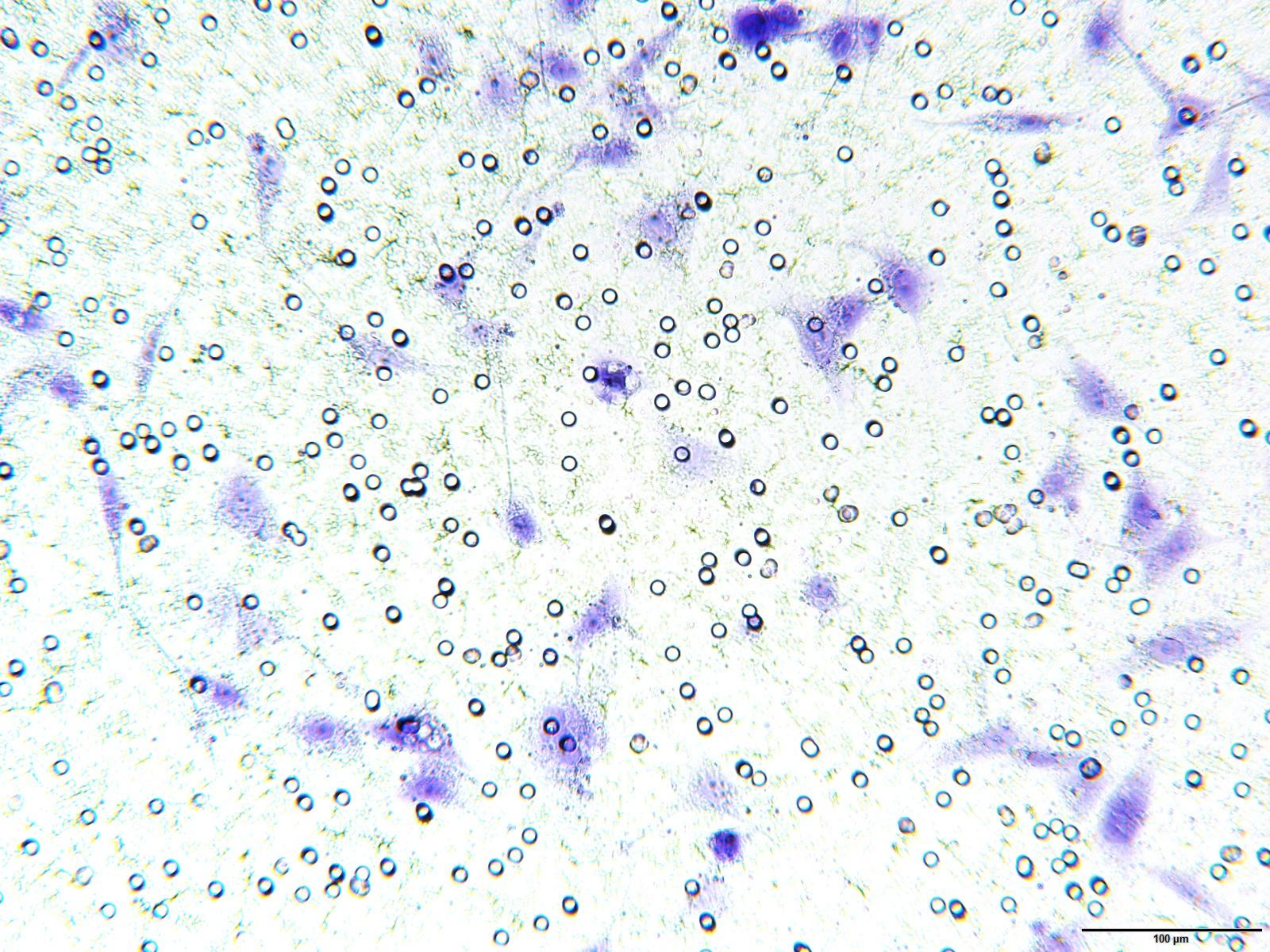
100 µm

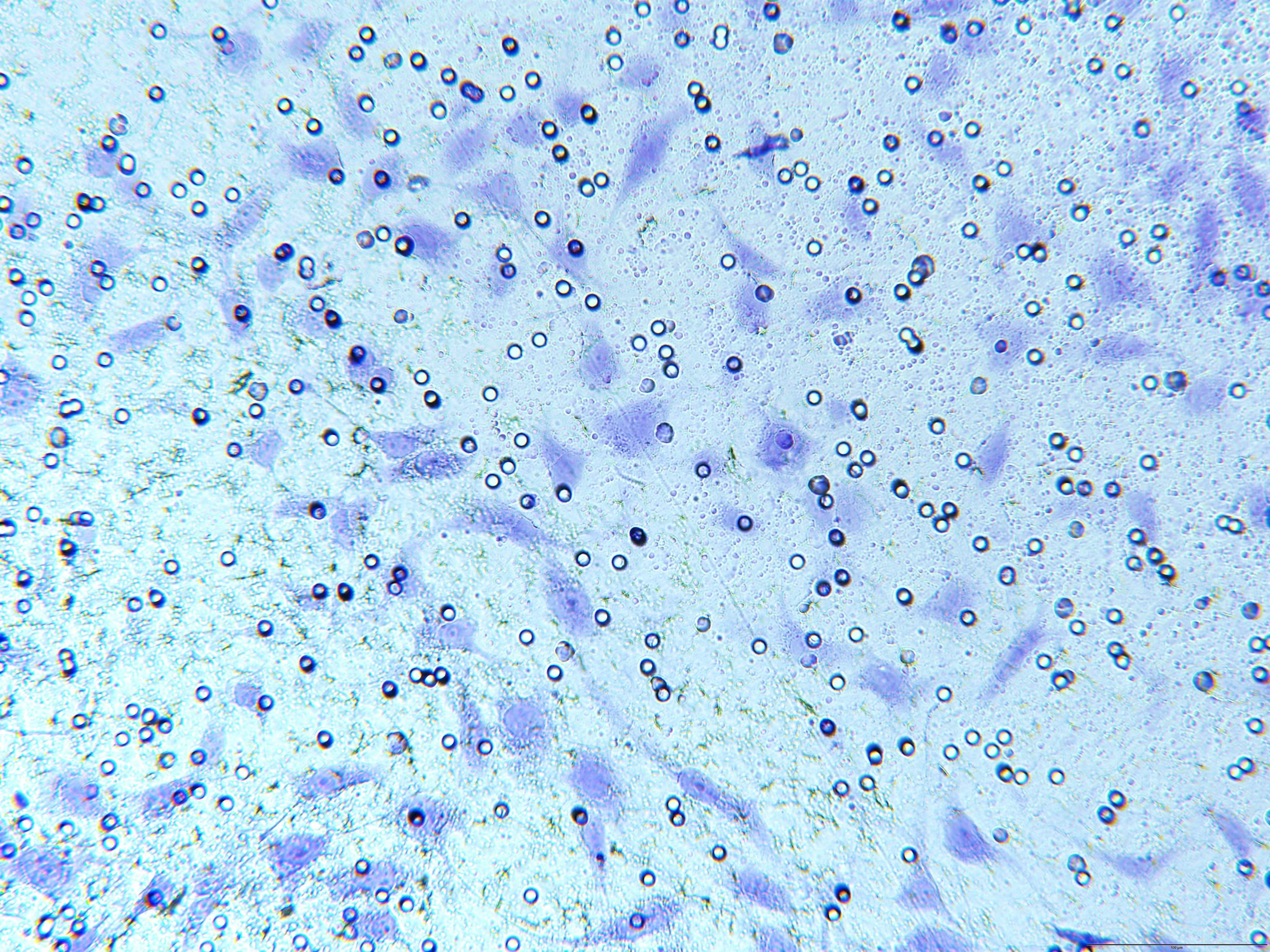
100 µm

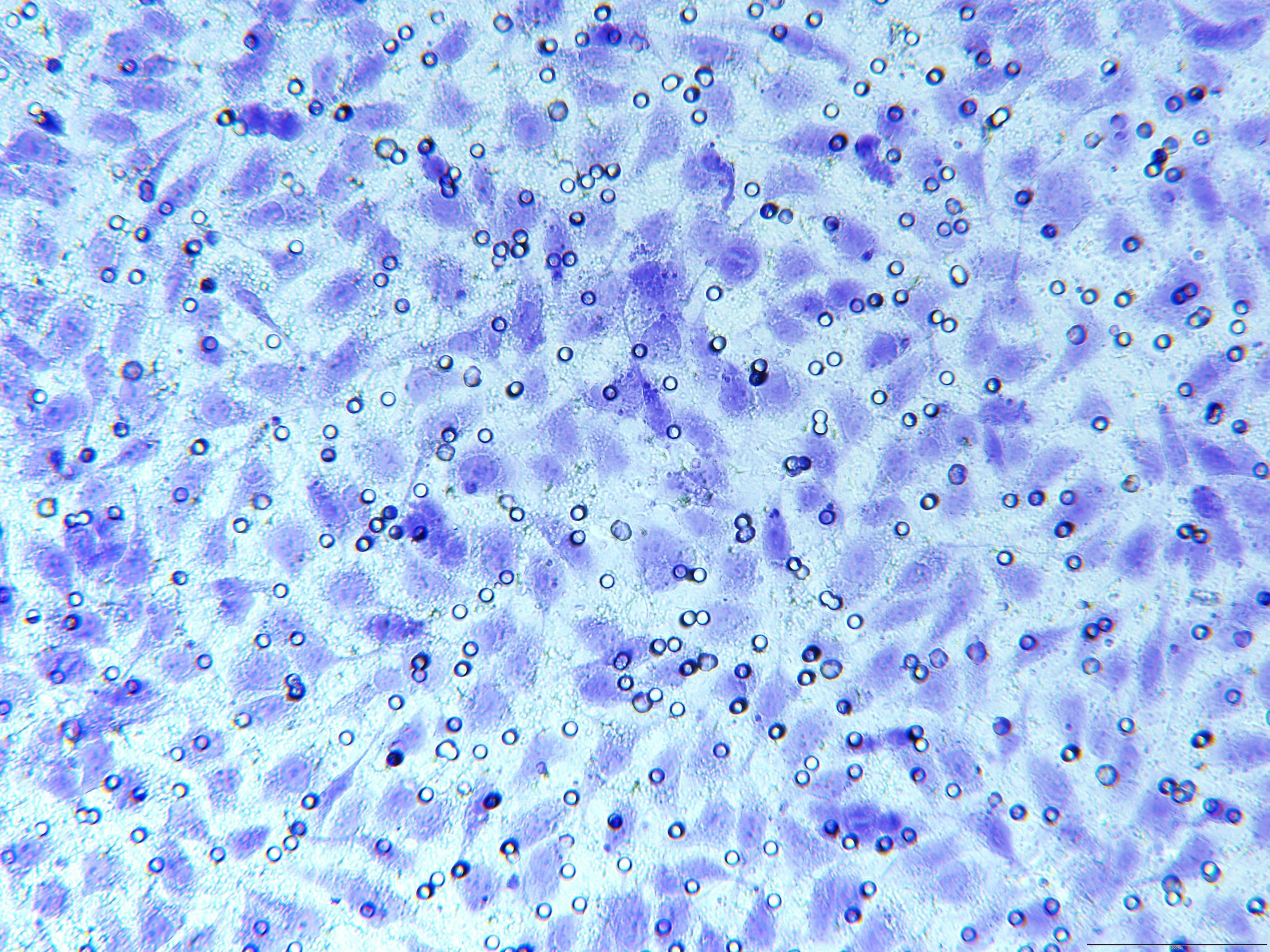

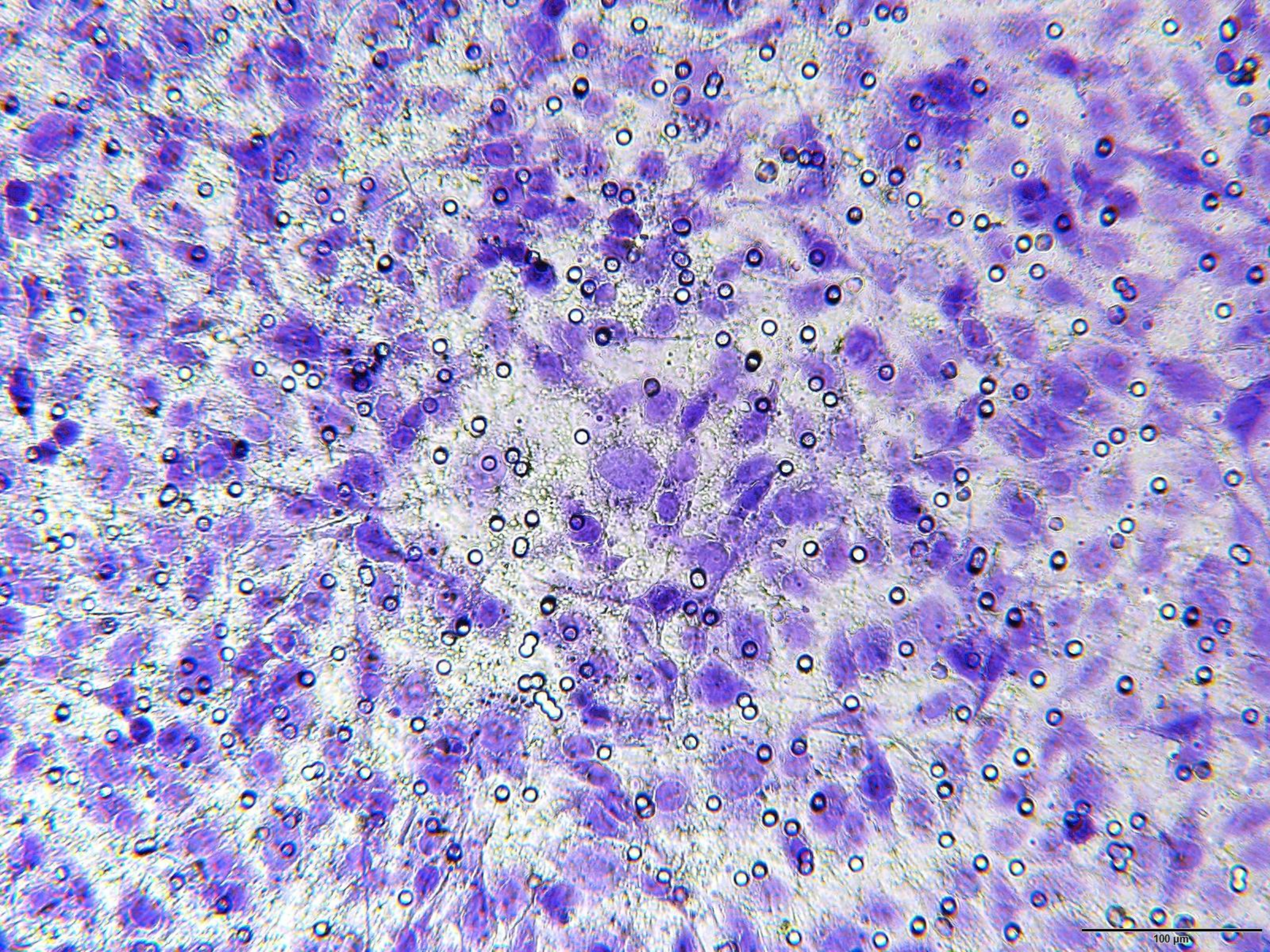
100 µm

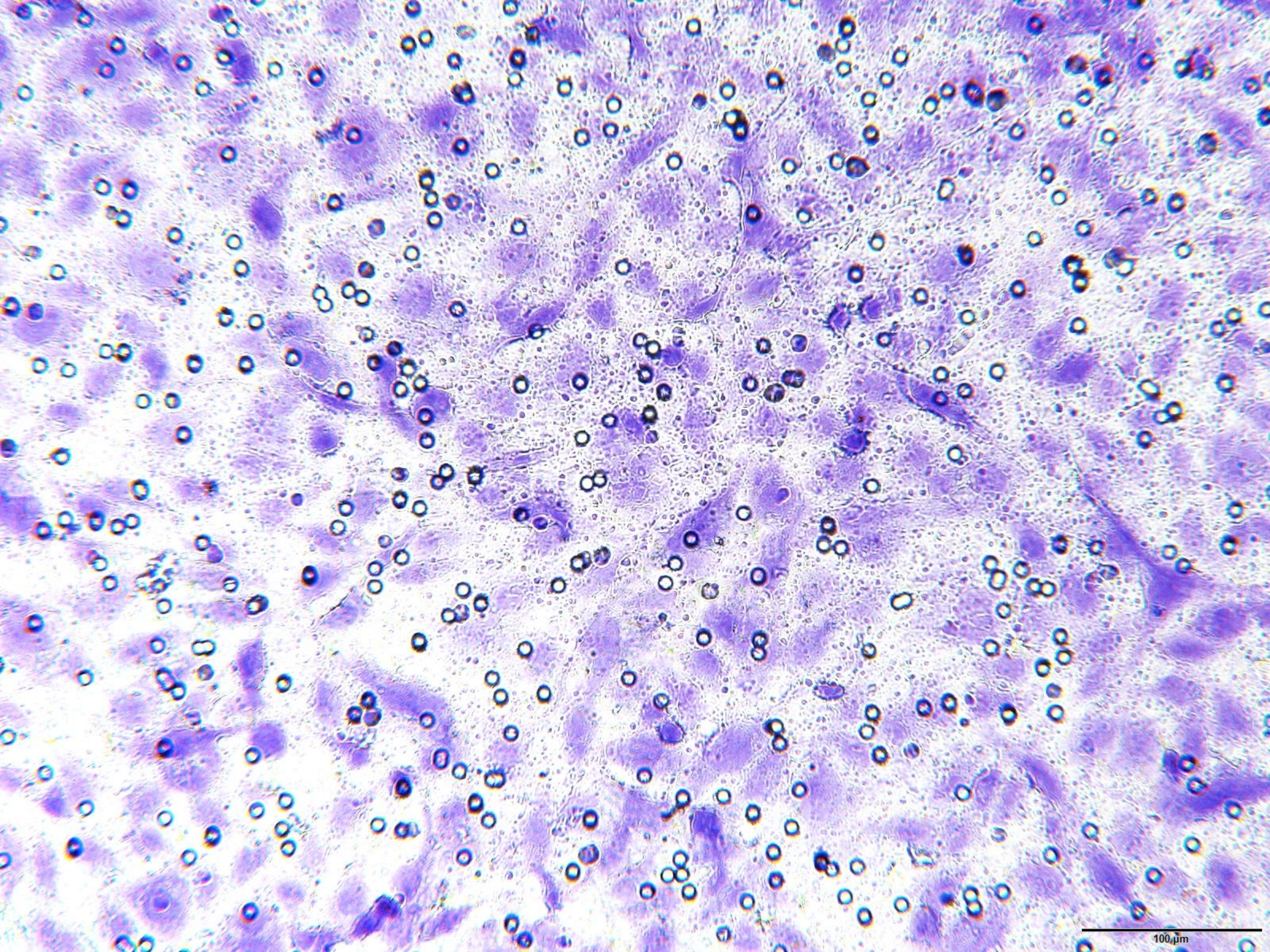
100 µm

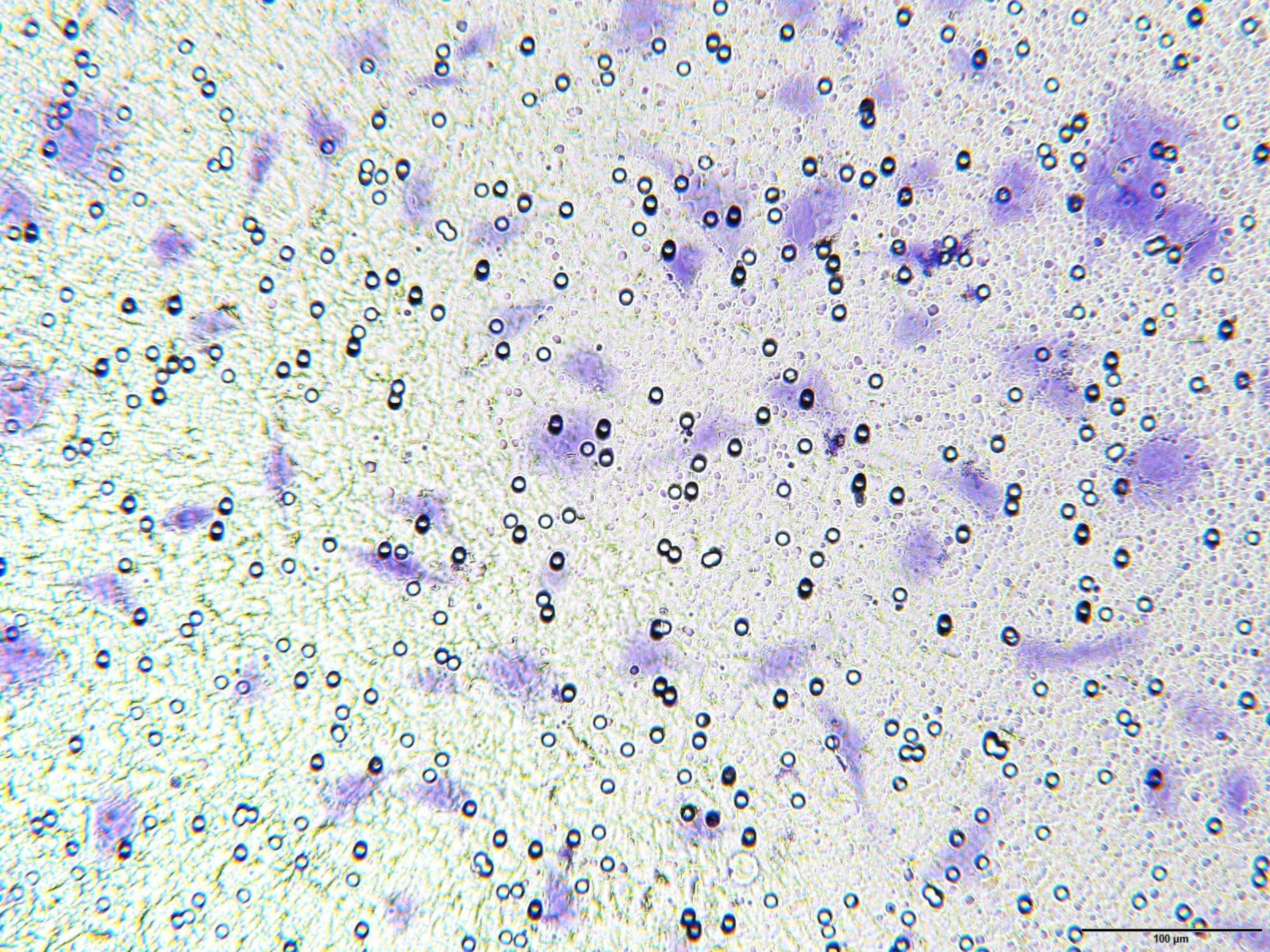
100 µm

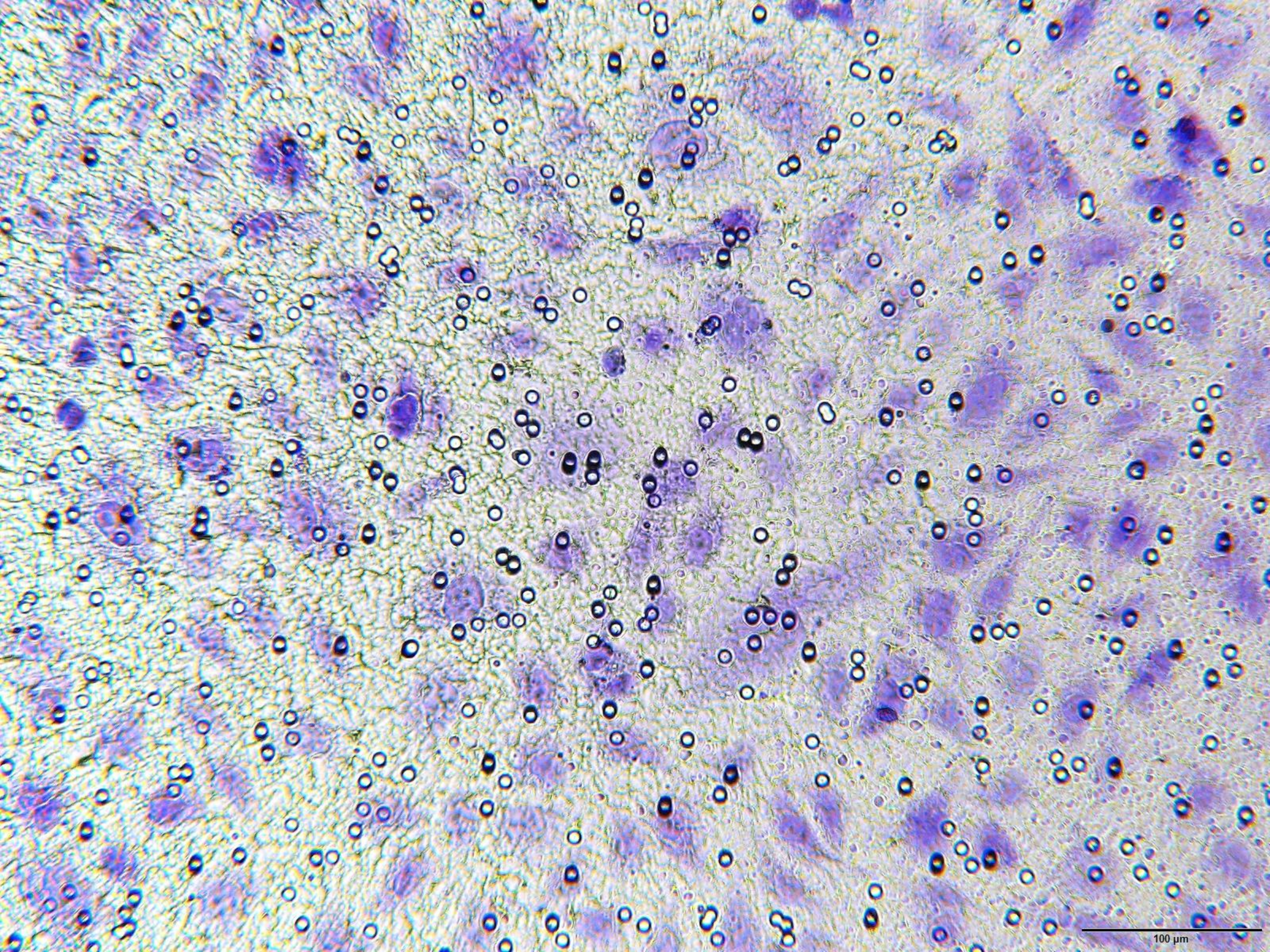
100 μm

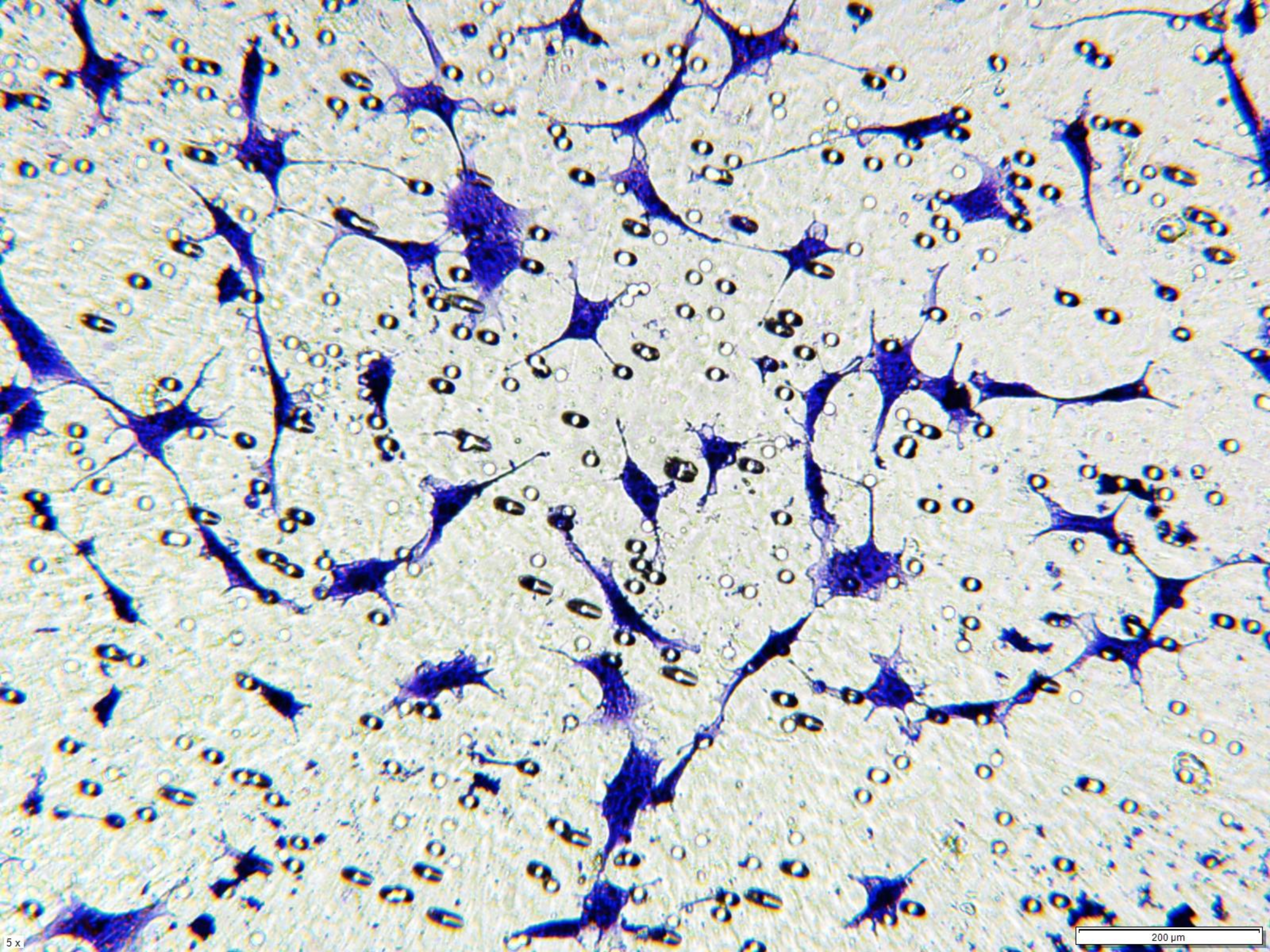
5 x
200 µm

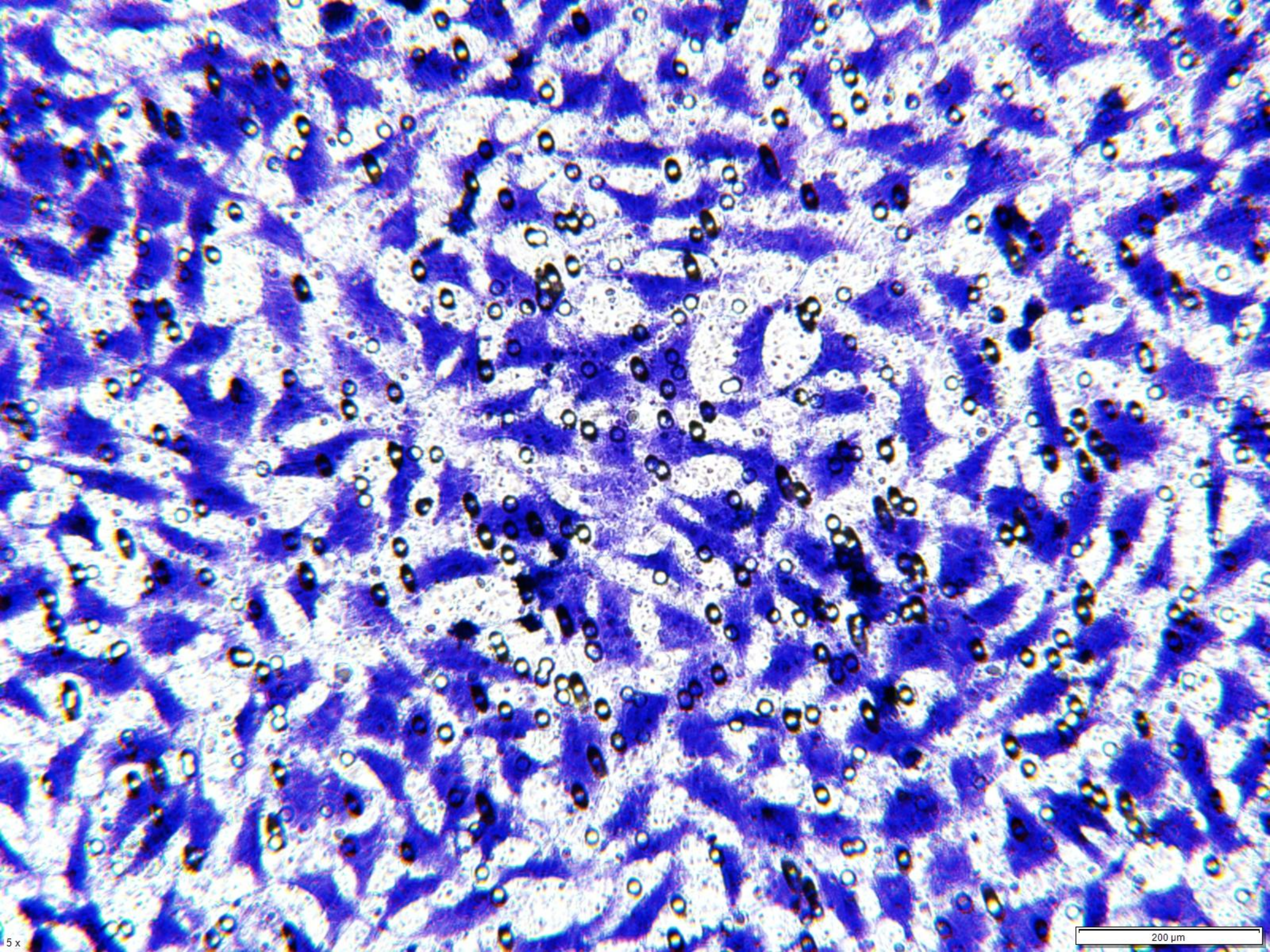
5 x
200 µm

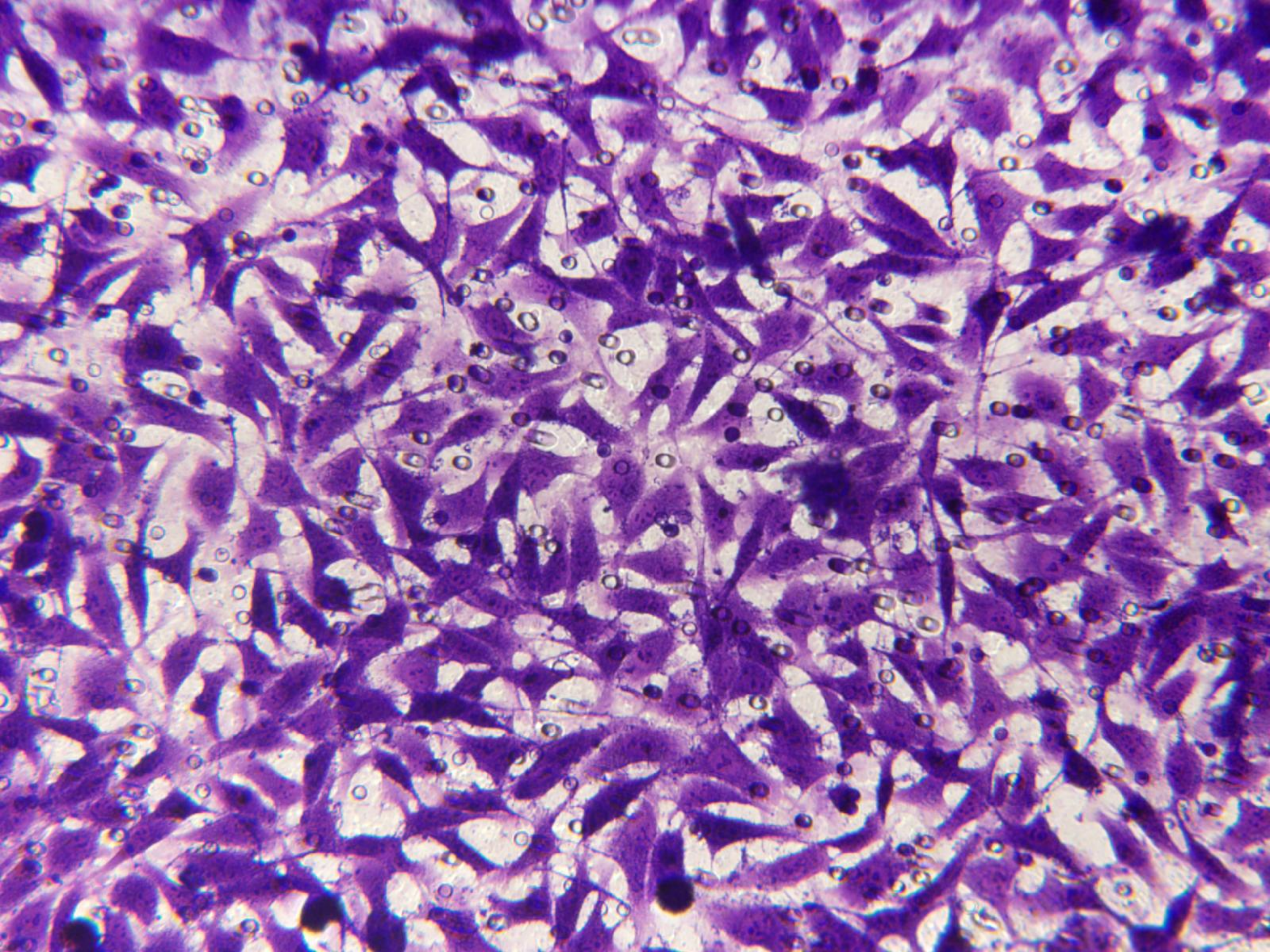

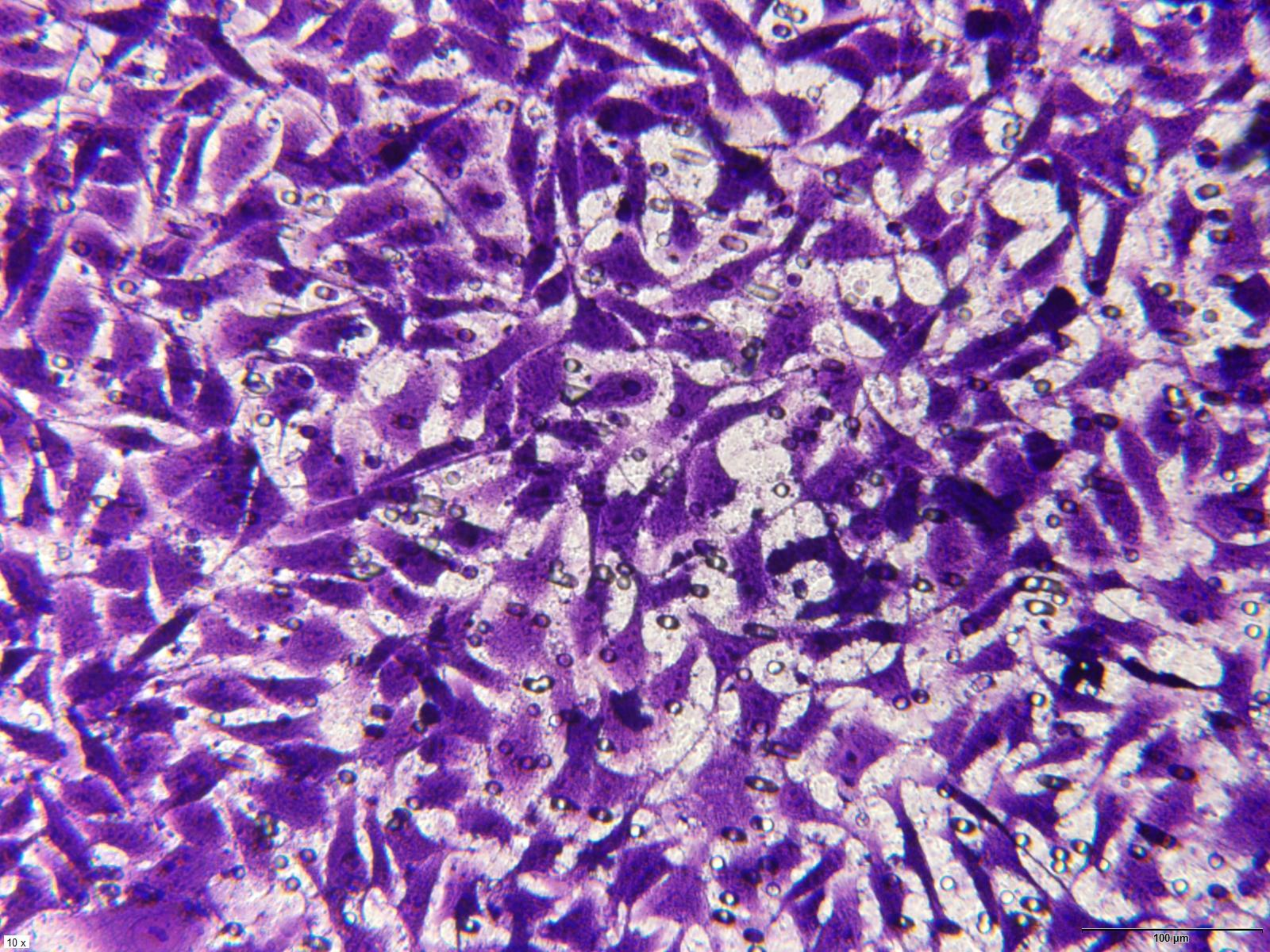
10 x
100 µm

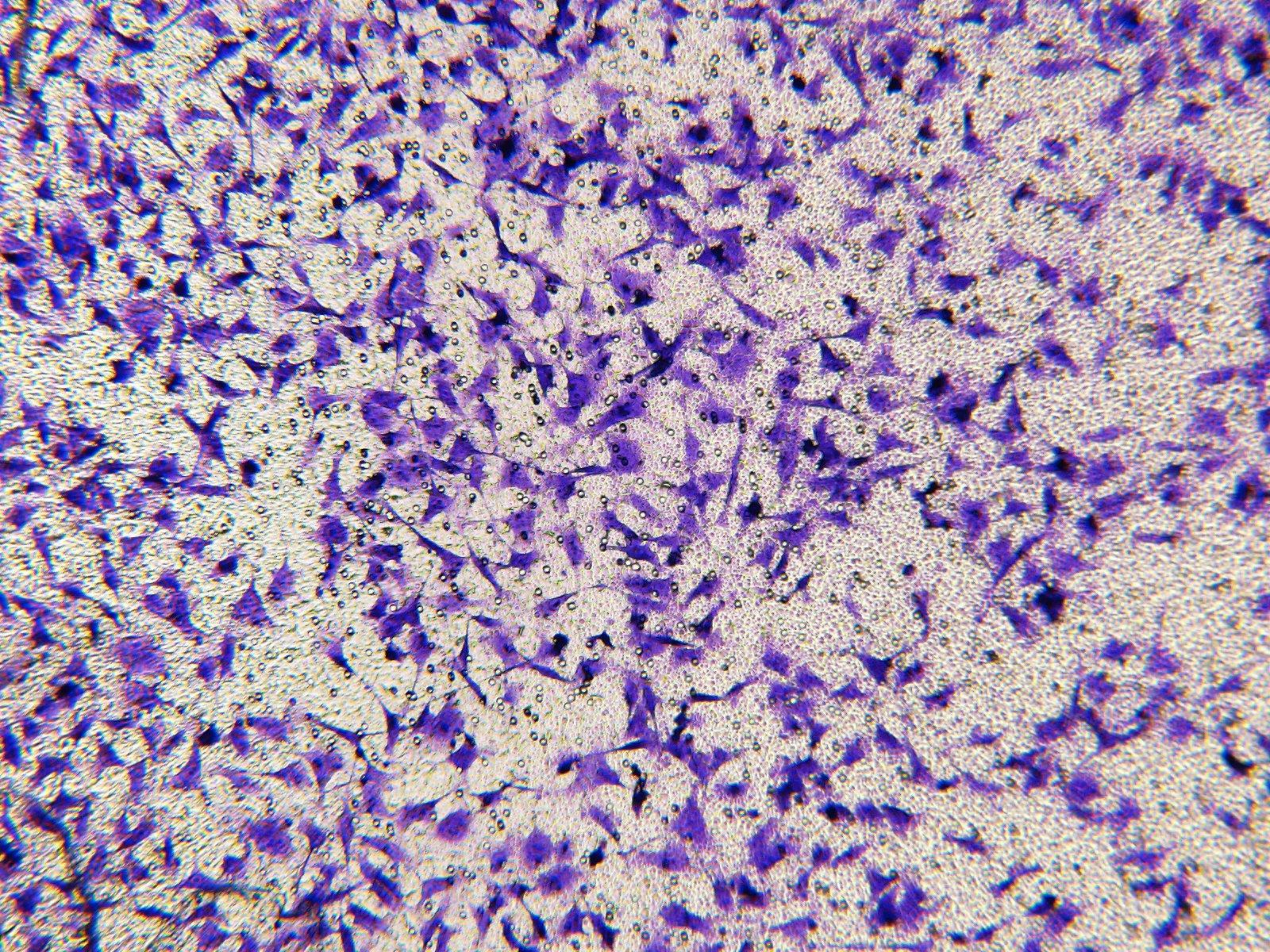

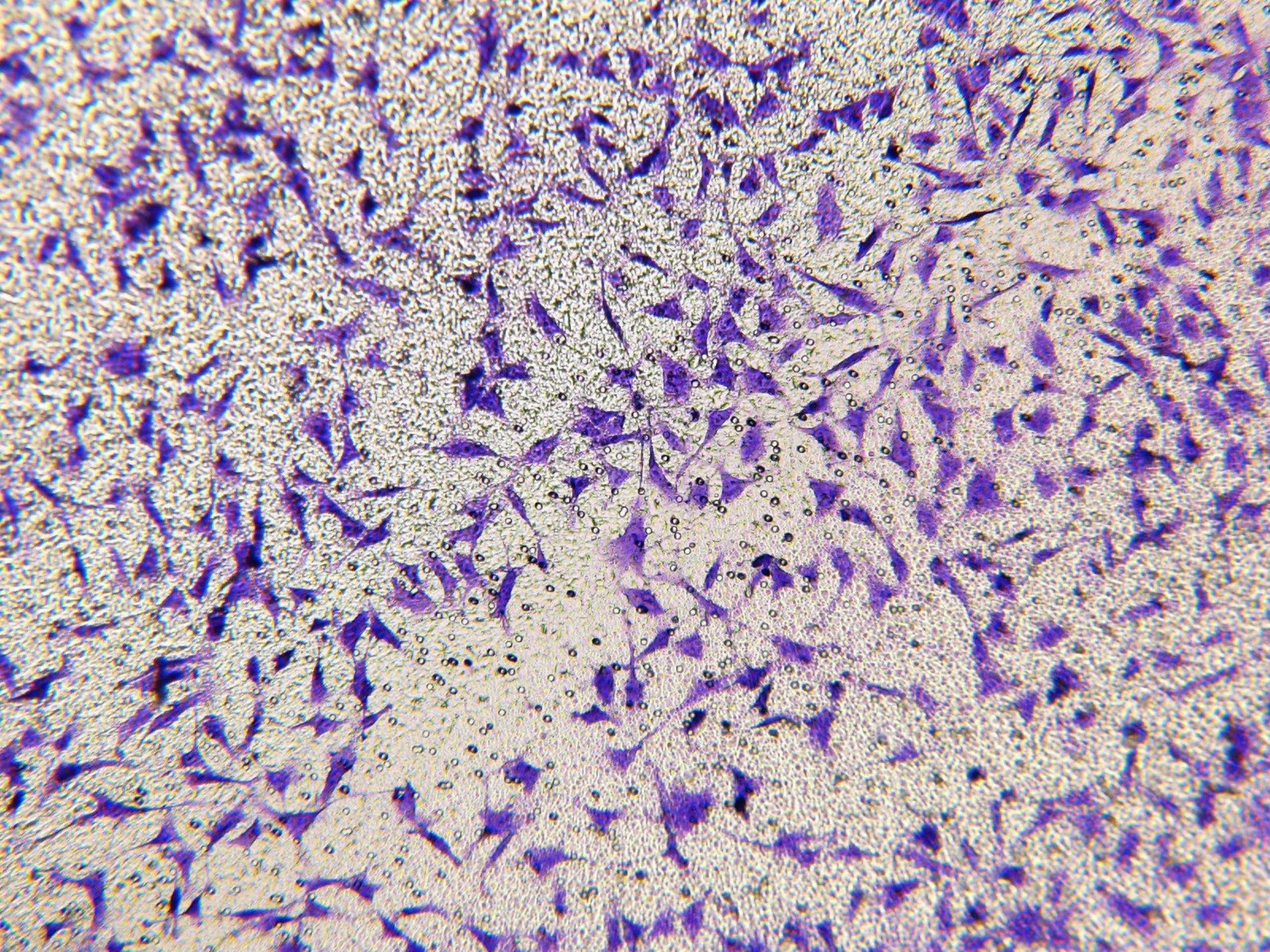

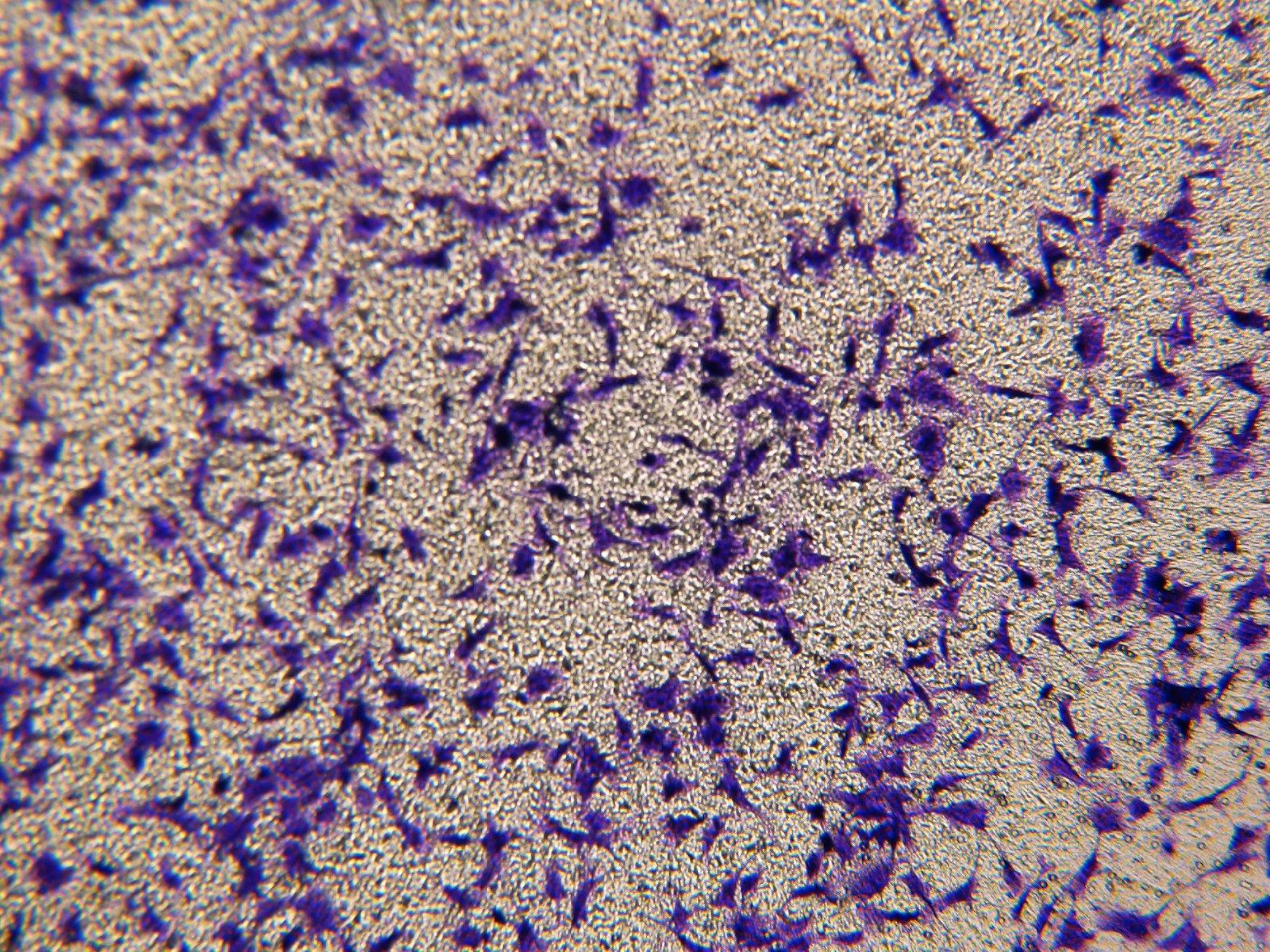

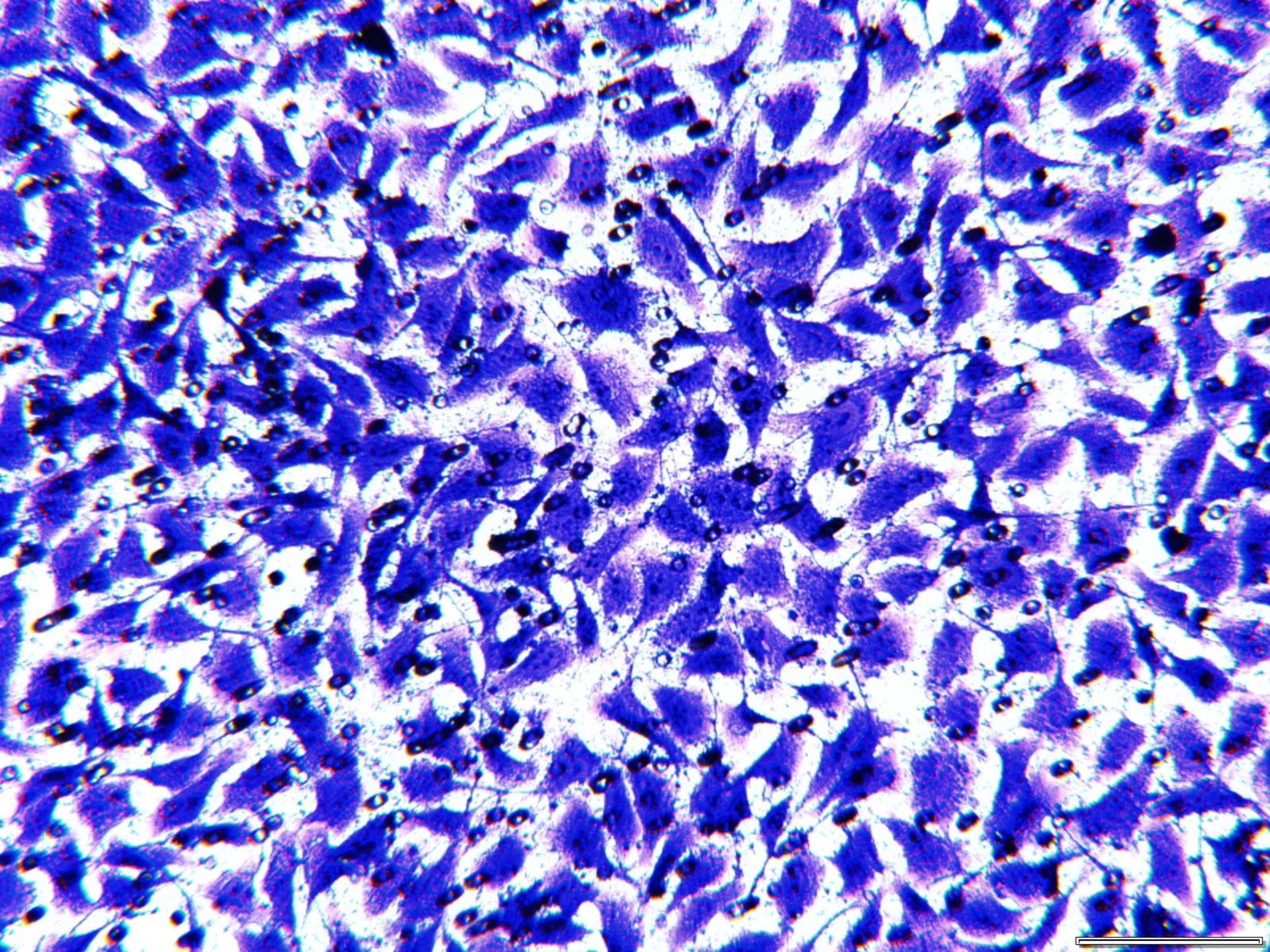

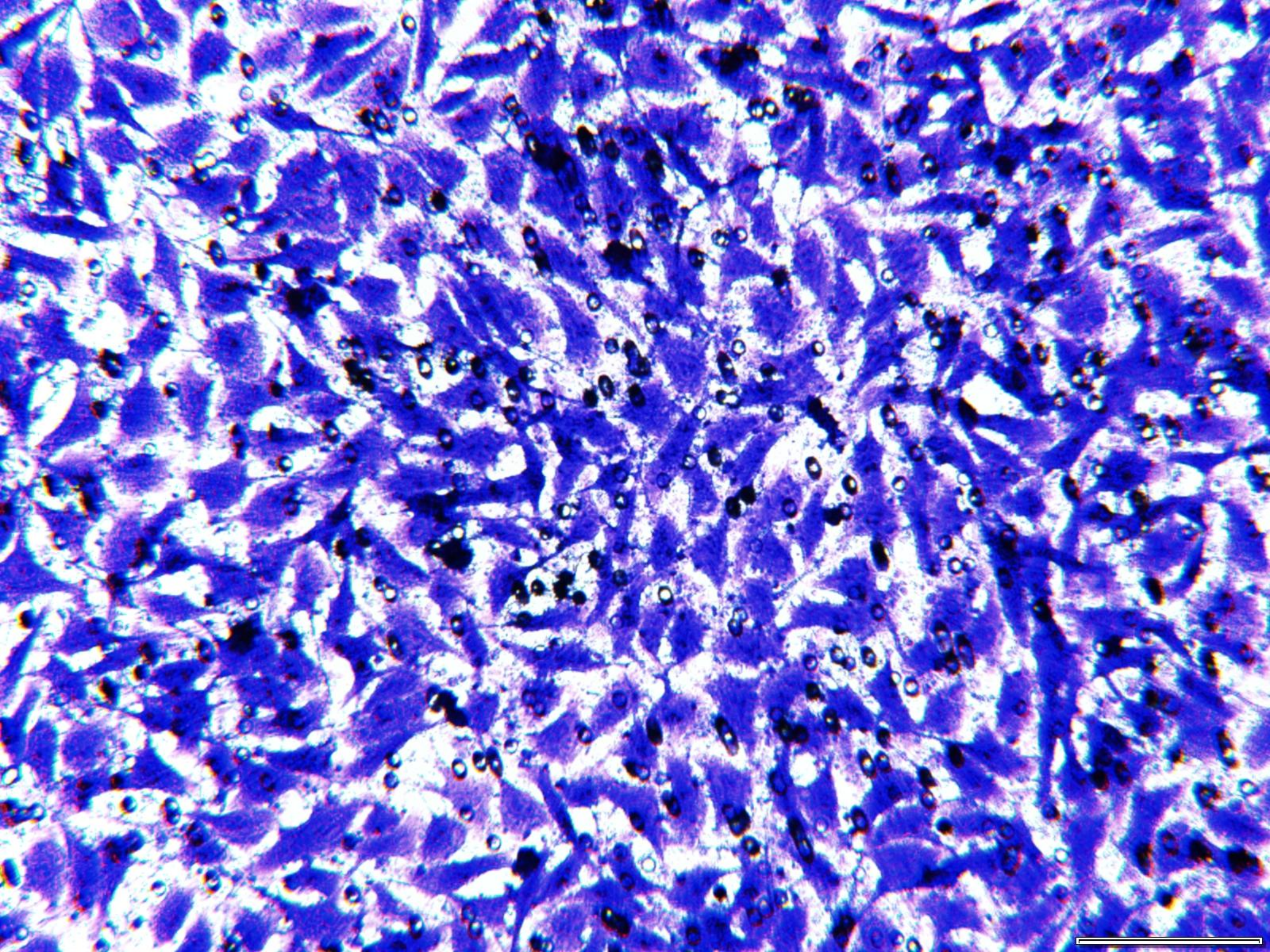

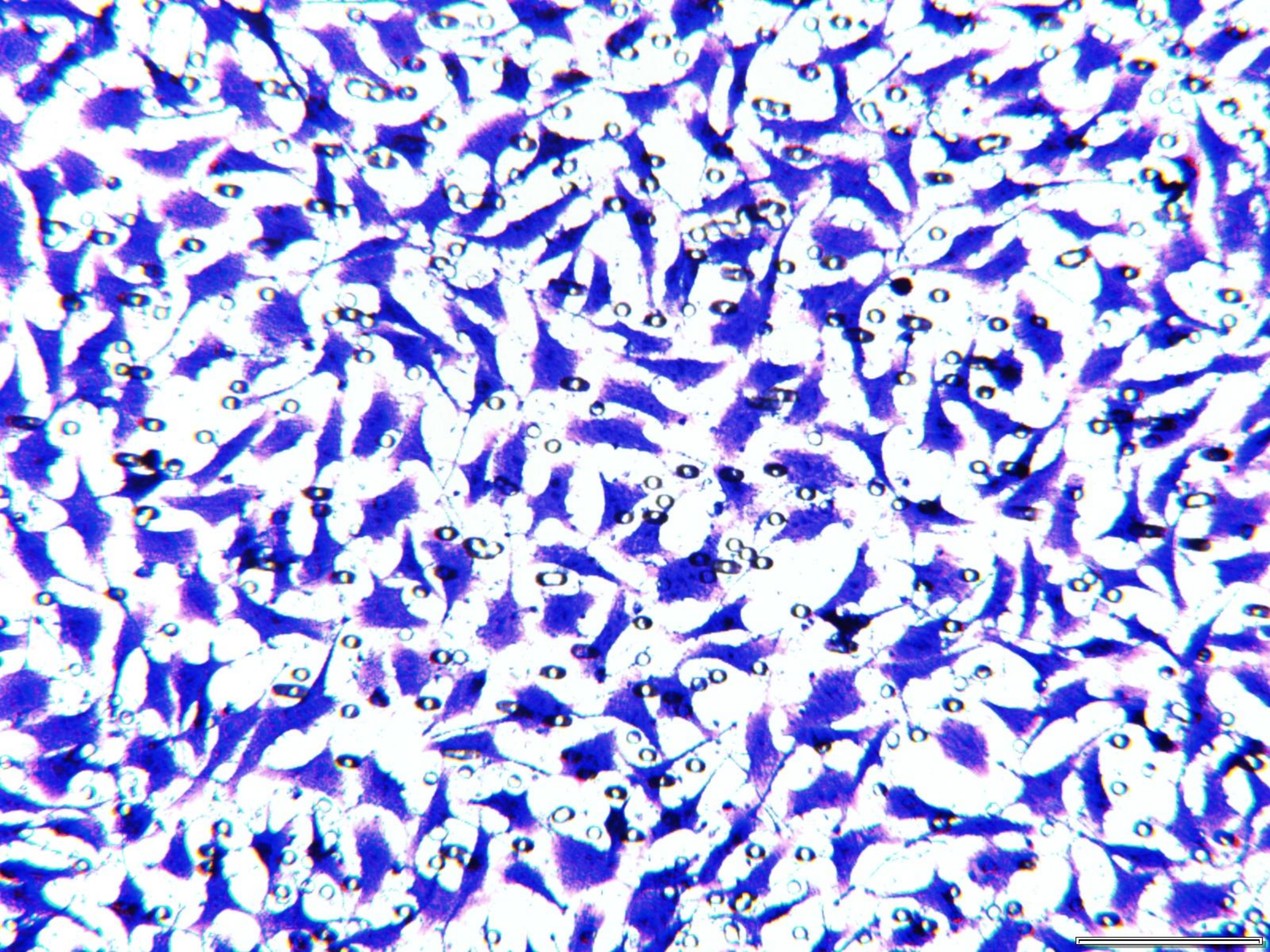

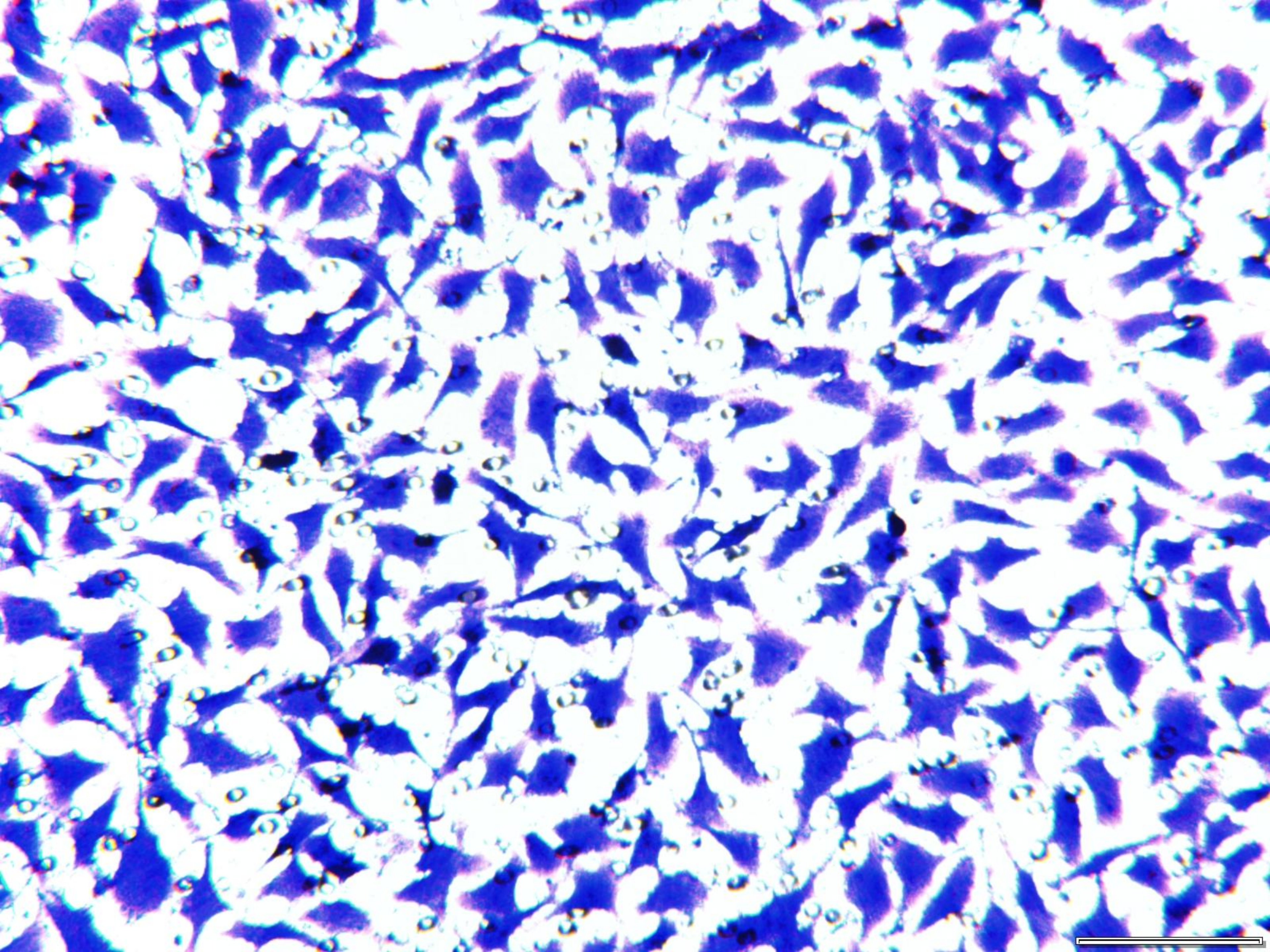

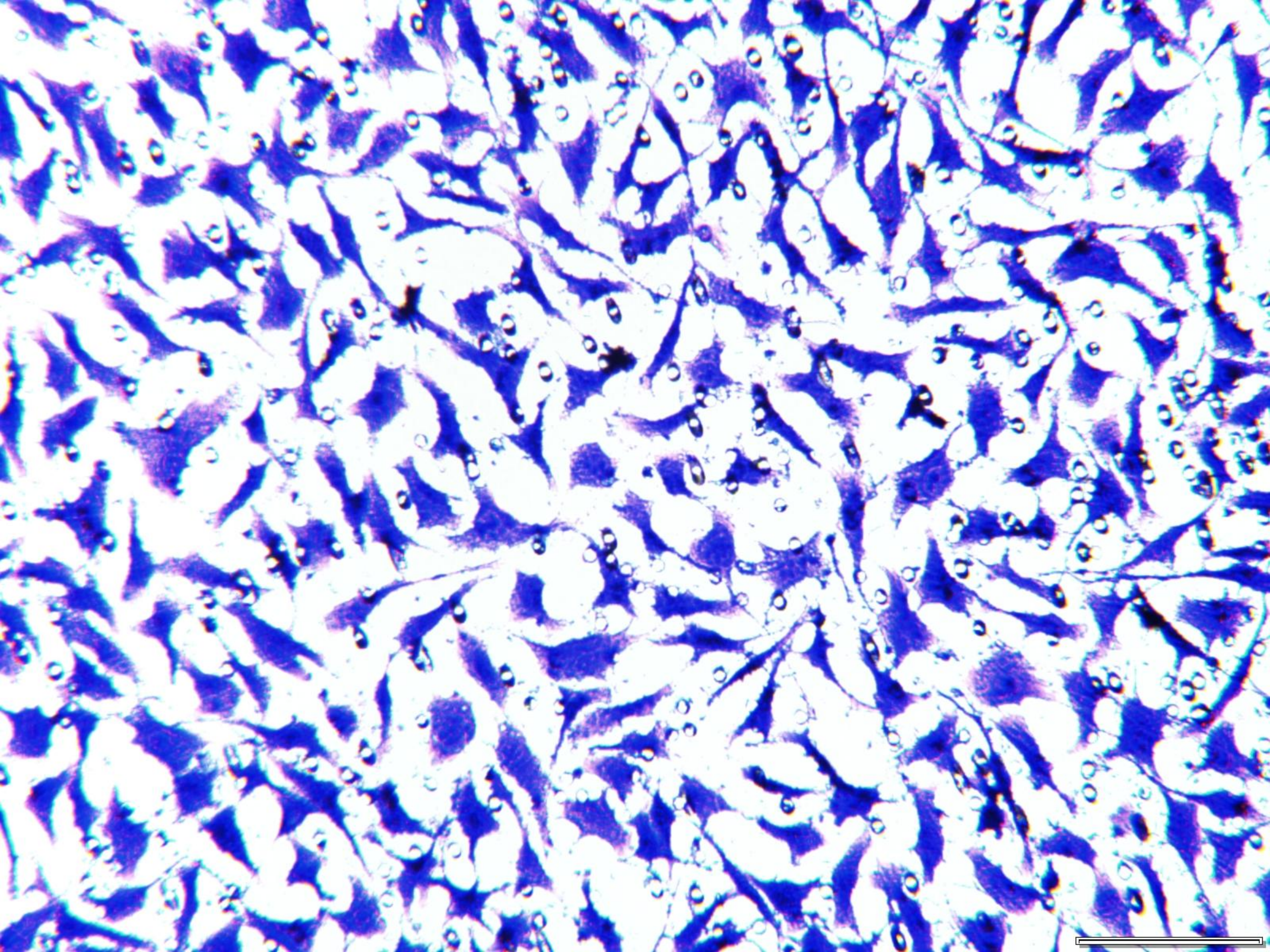

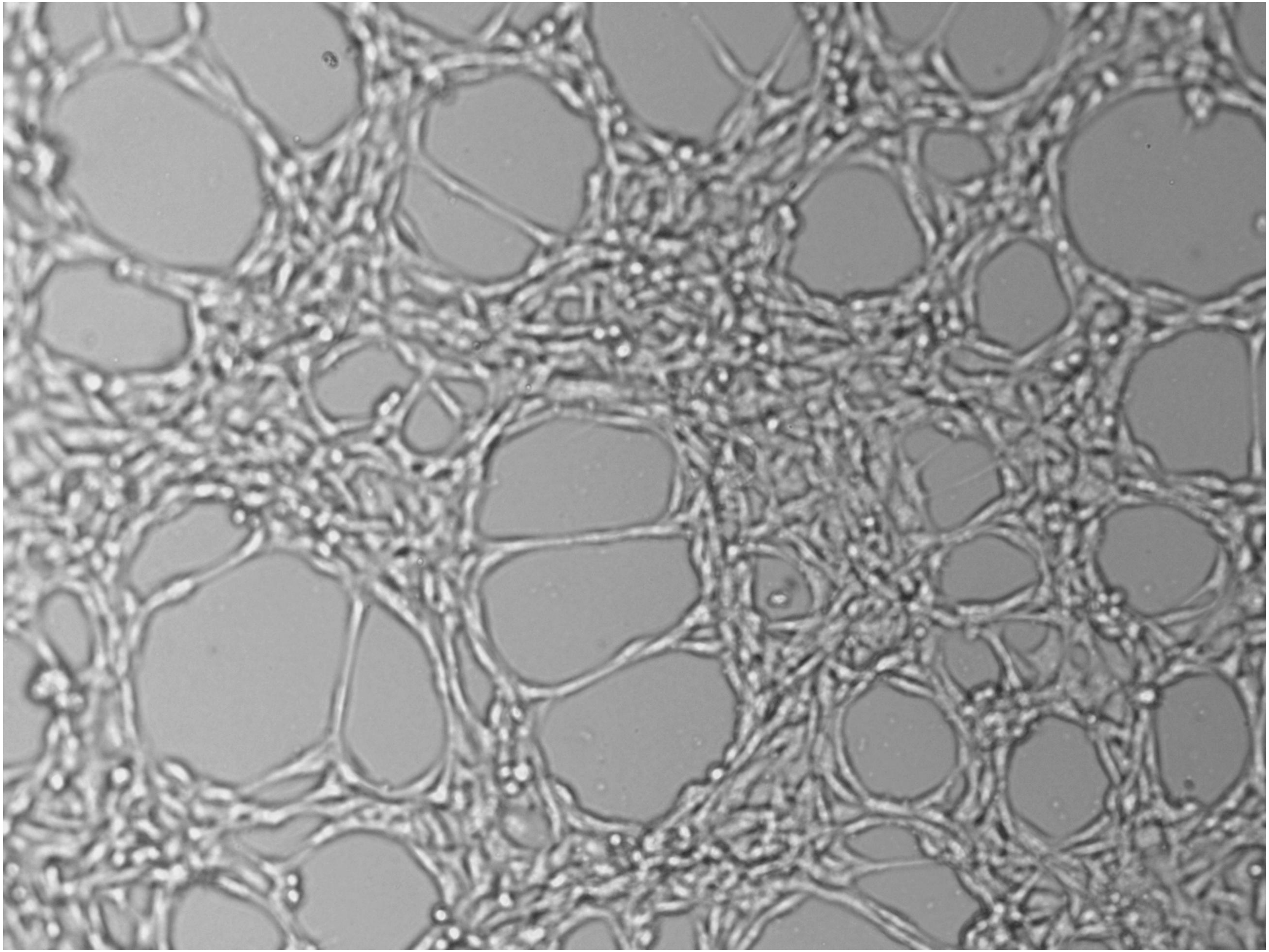

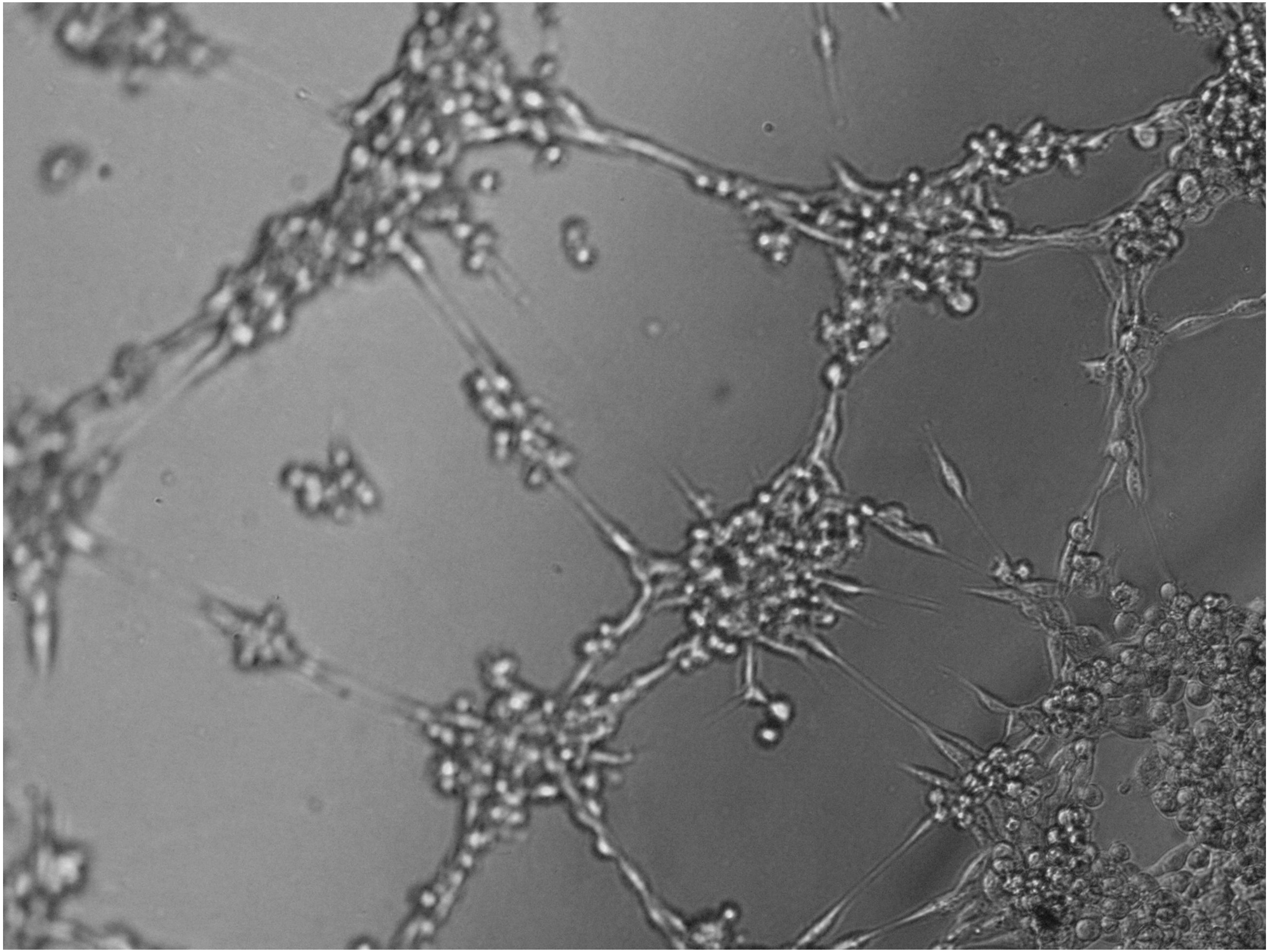

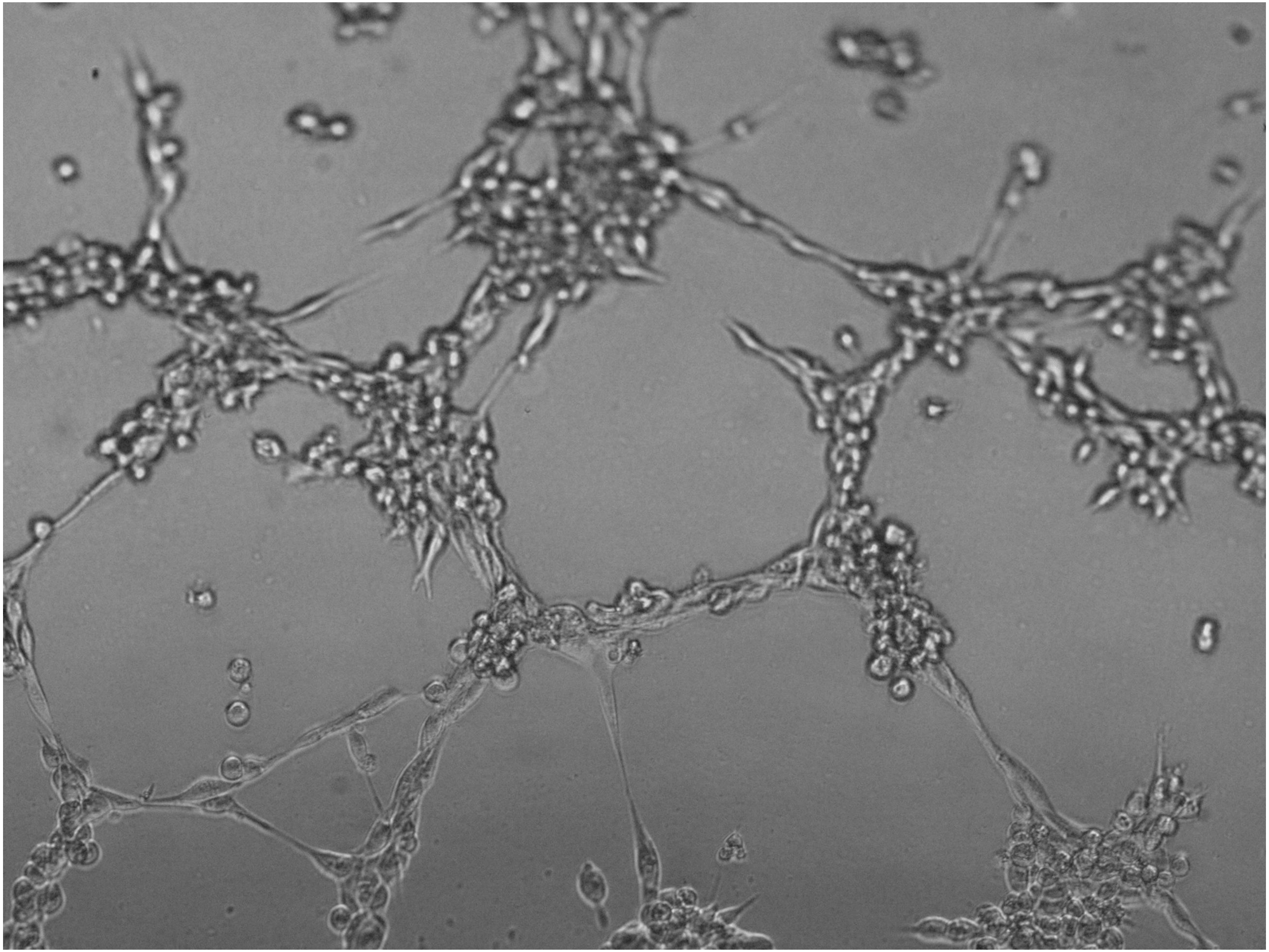

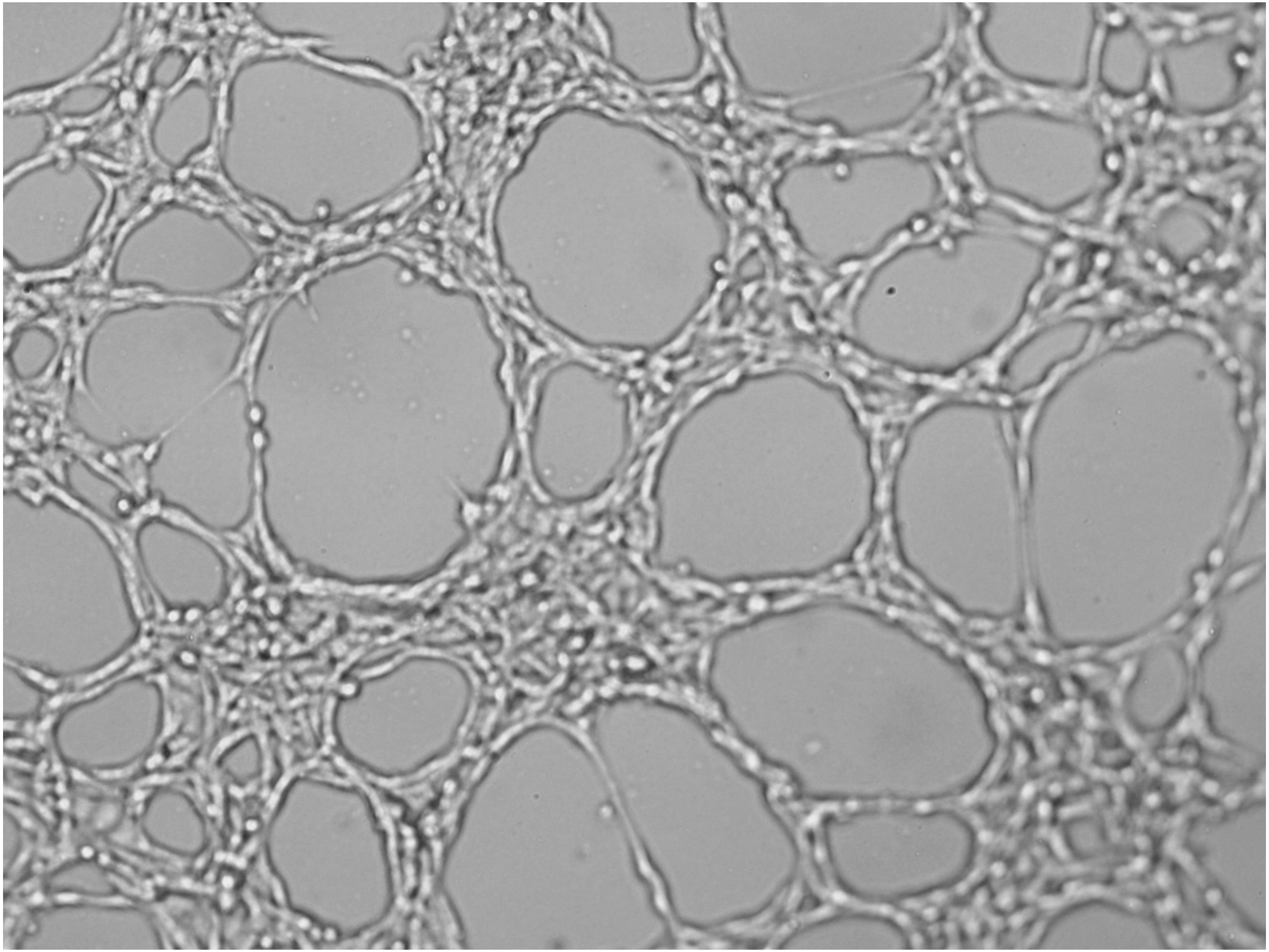

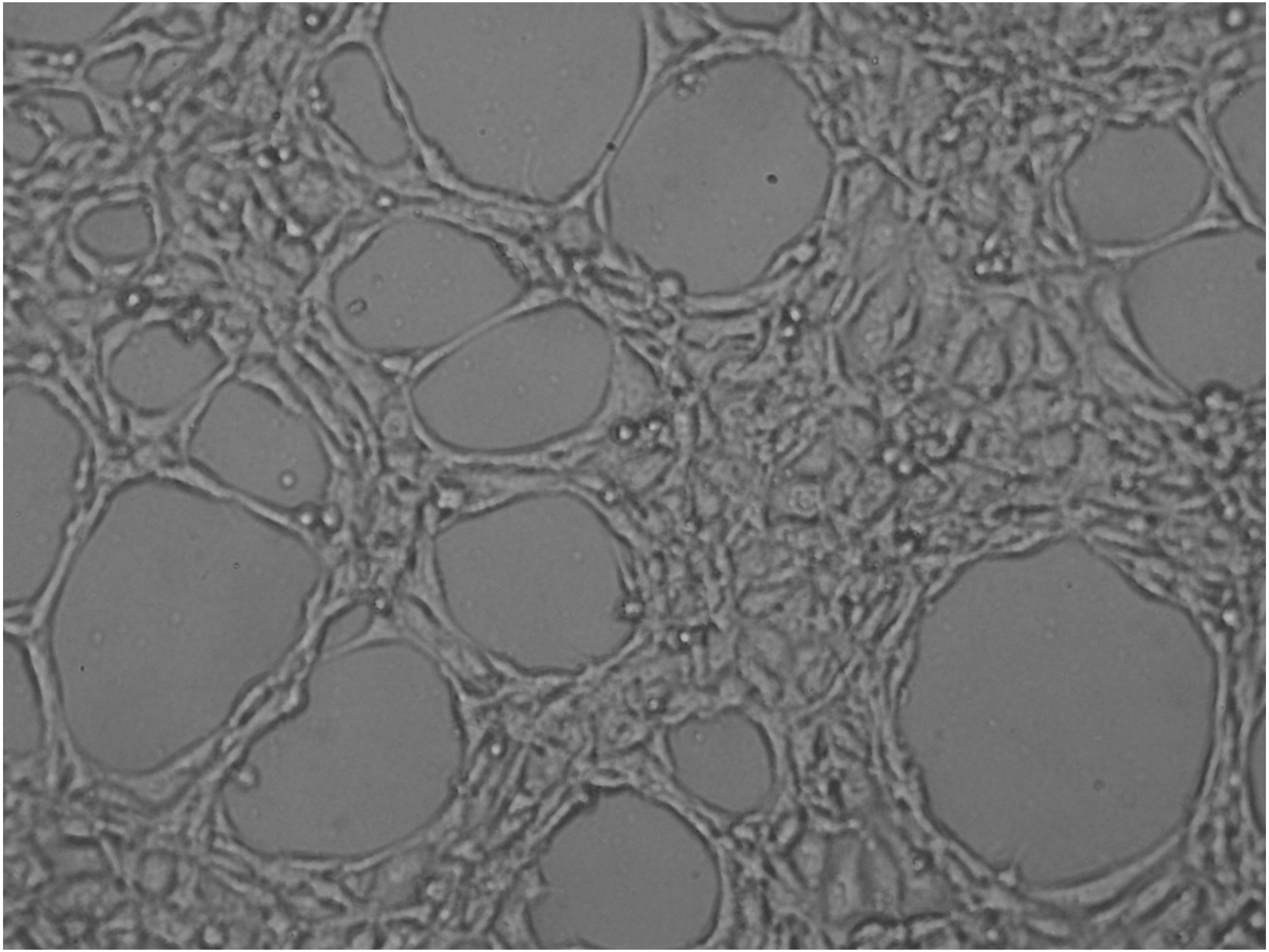

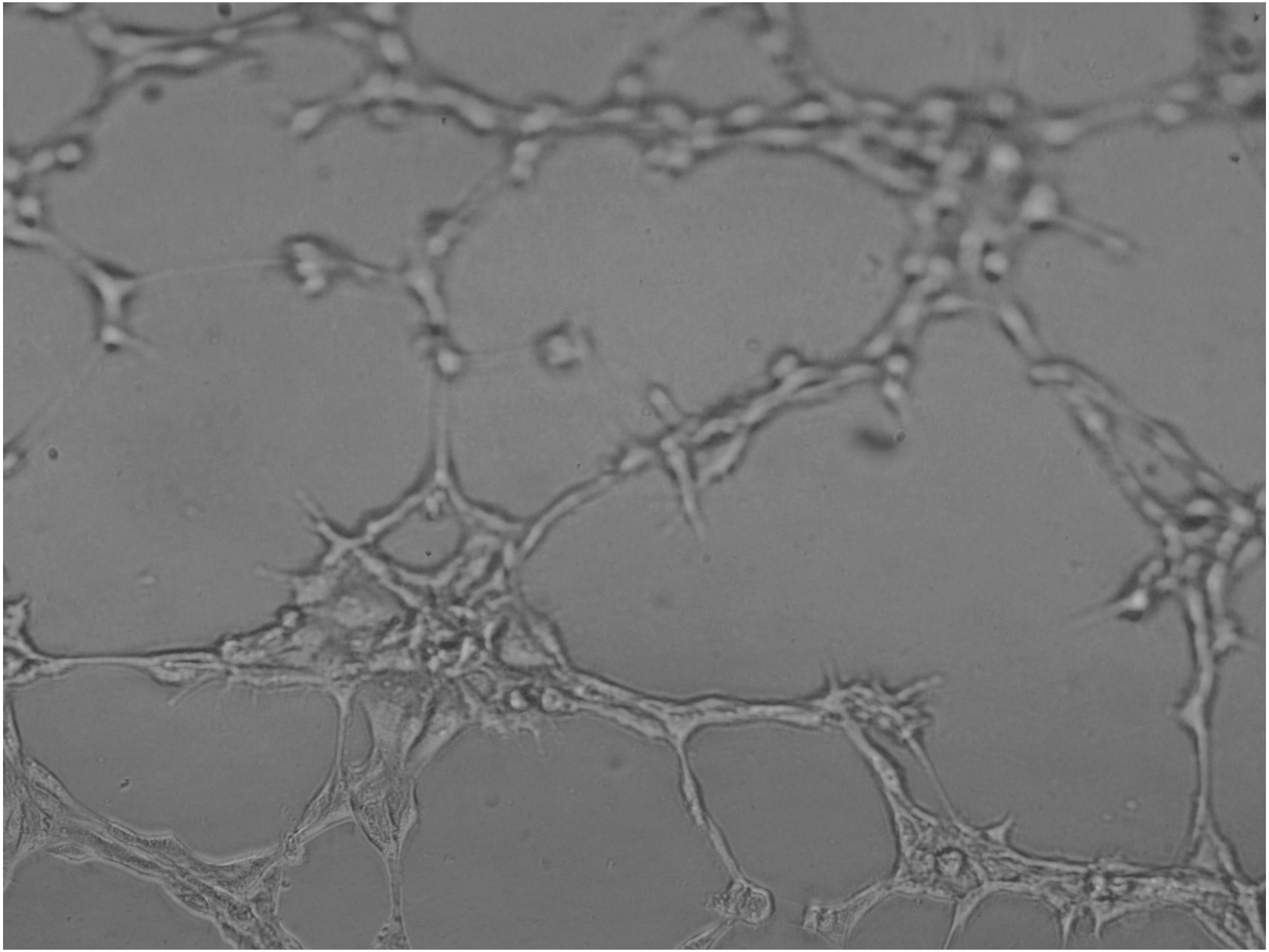

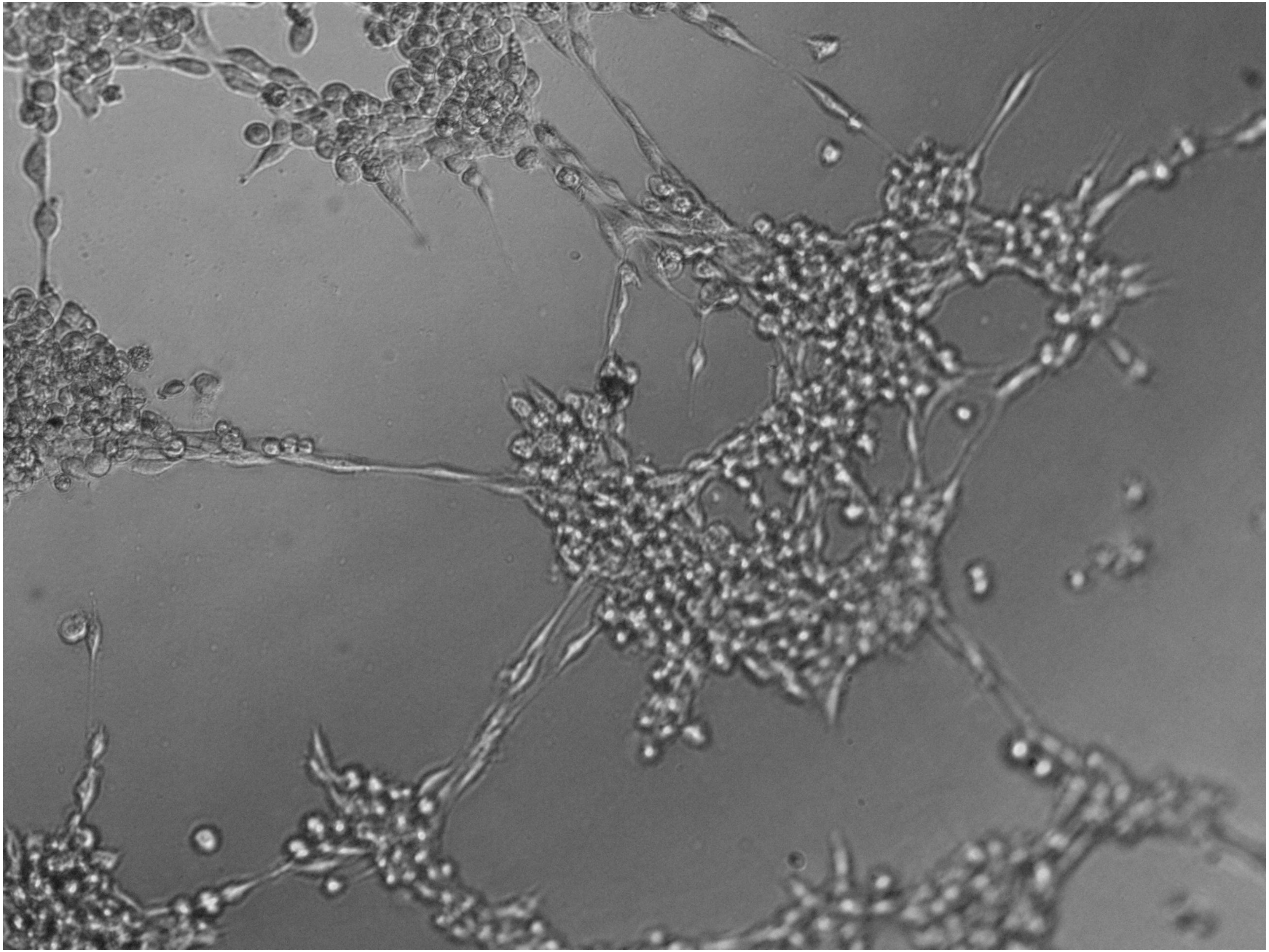

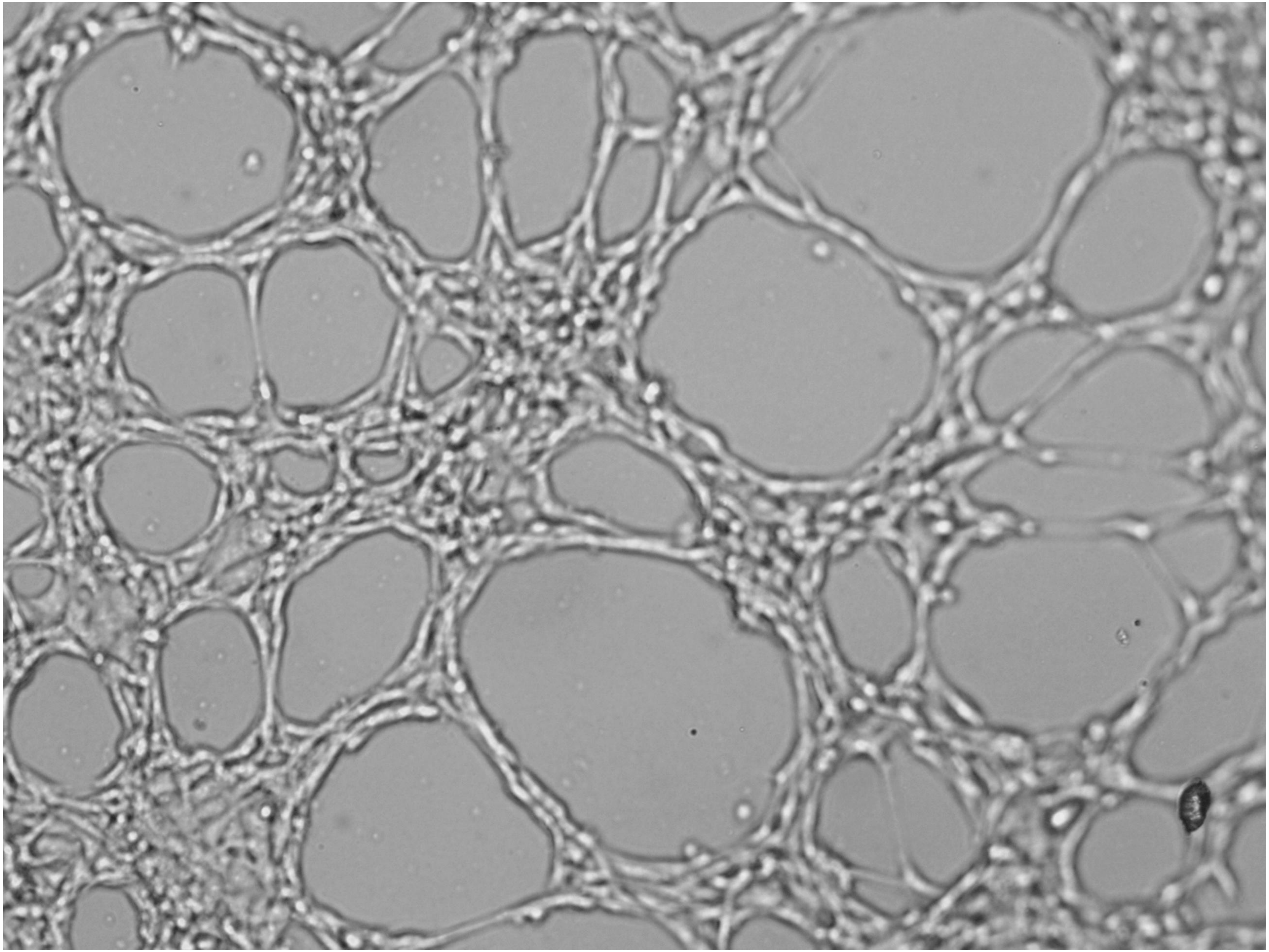

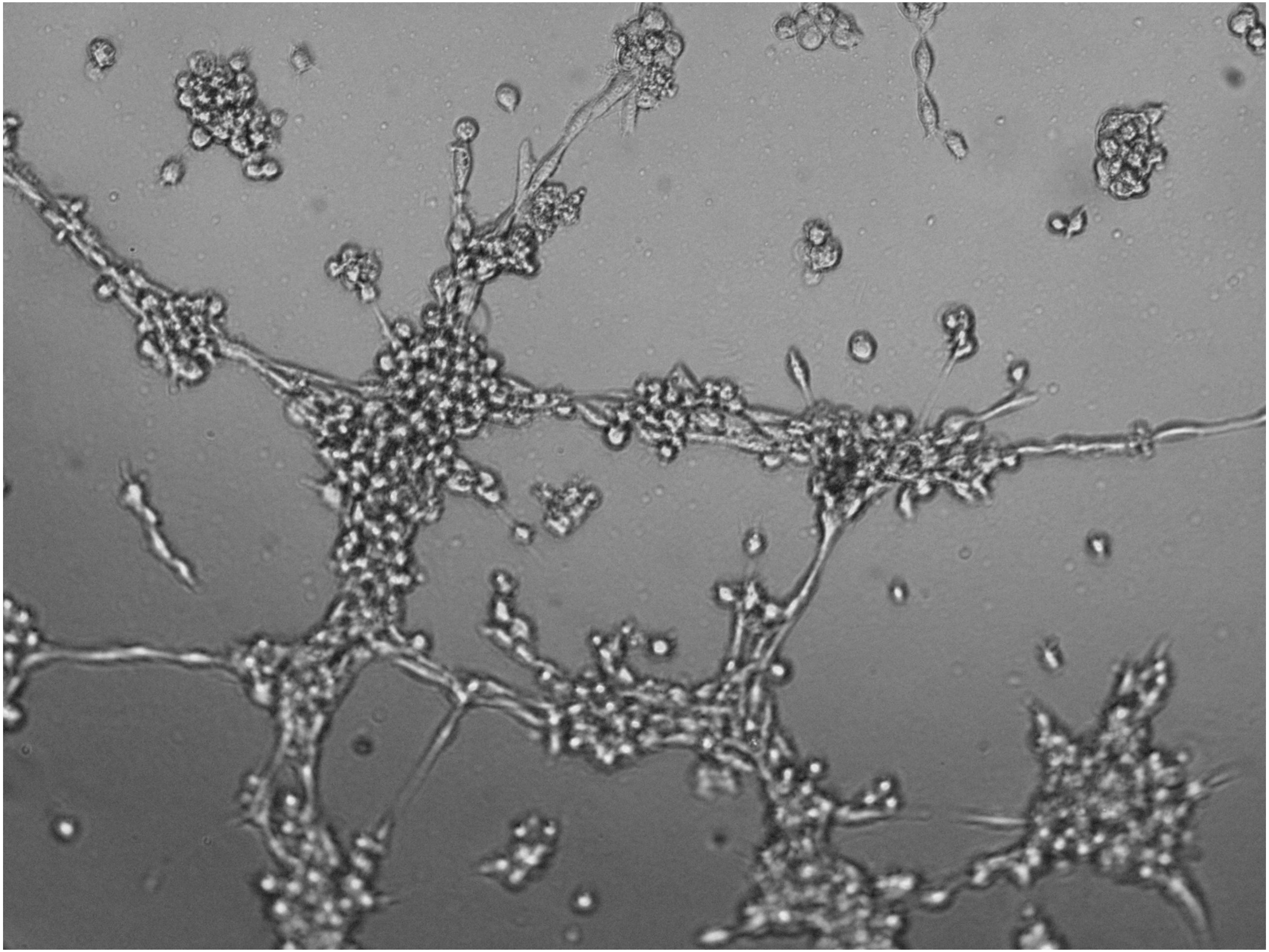

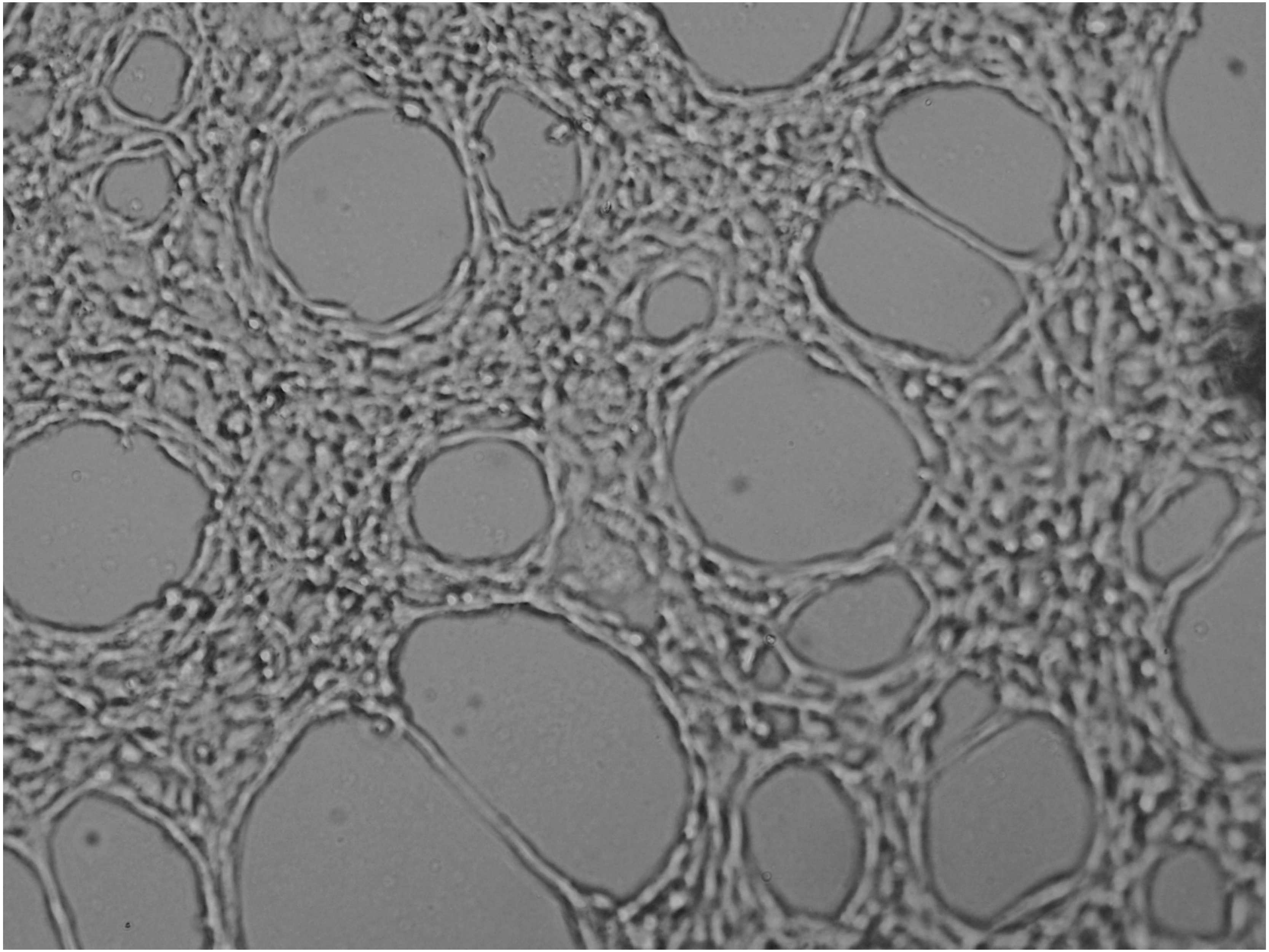

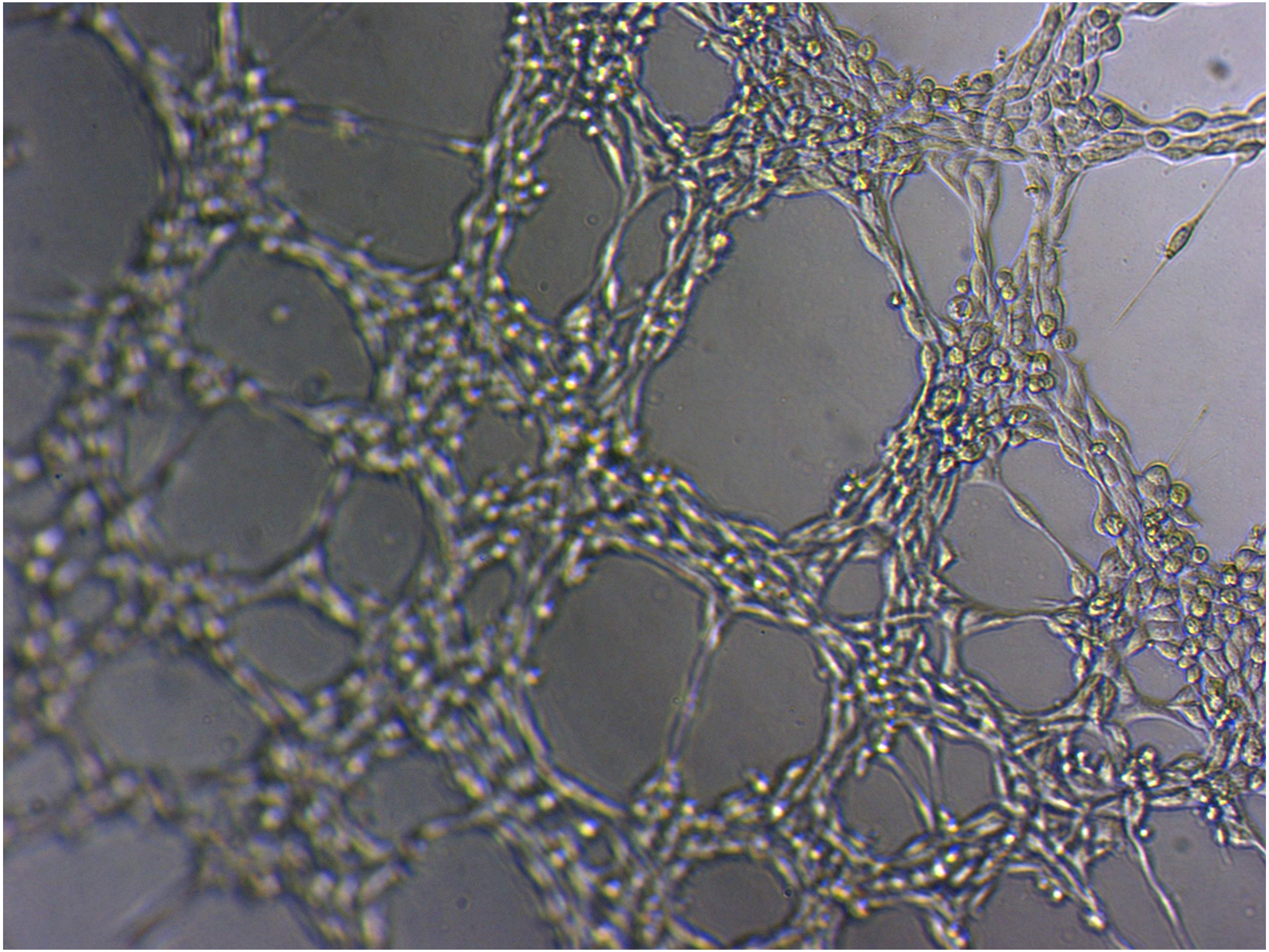

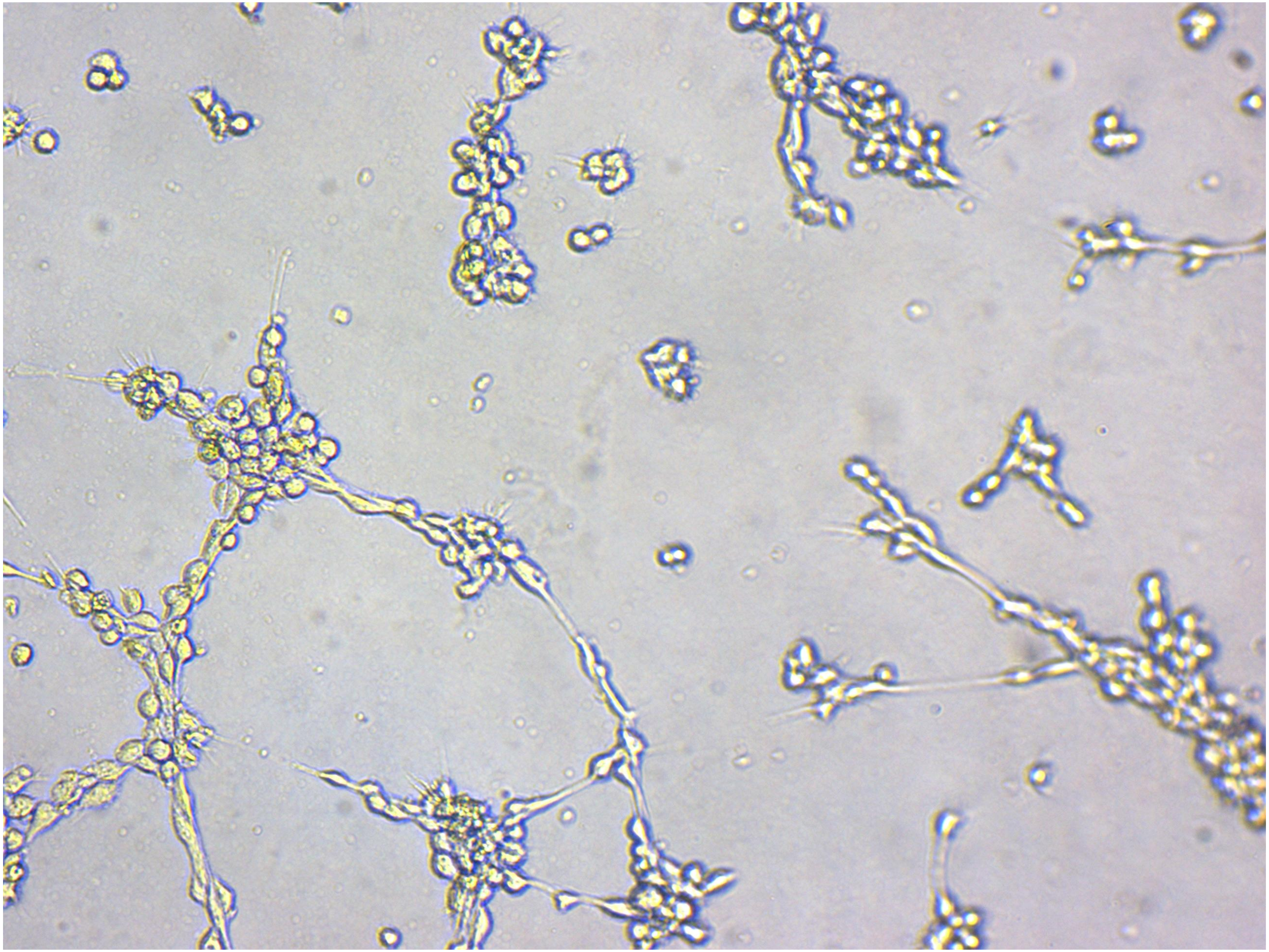

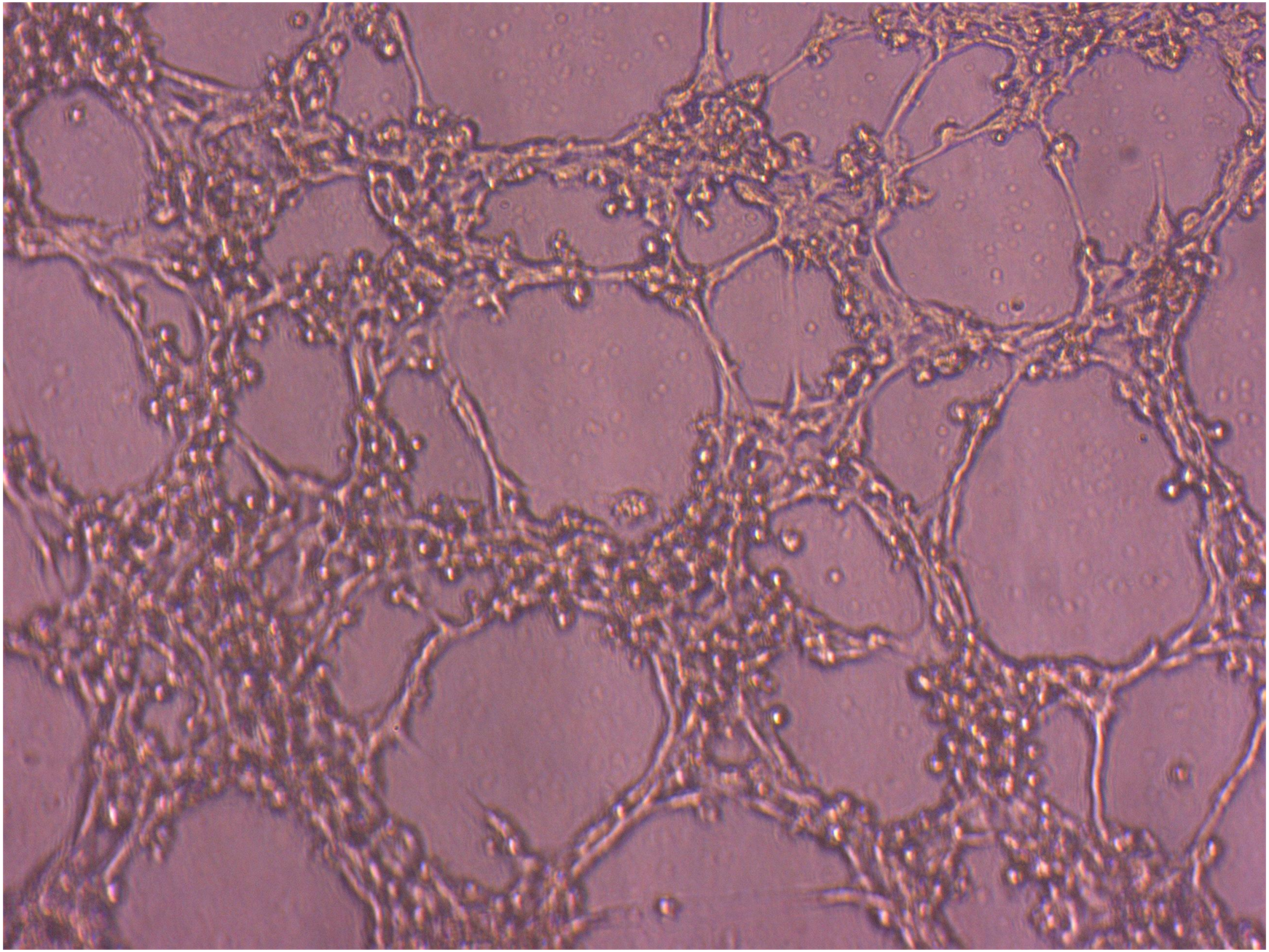

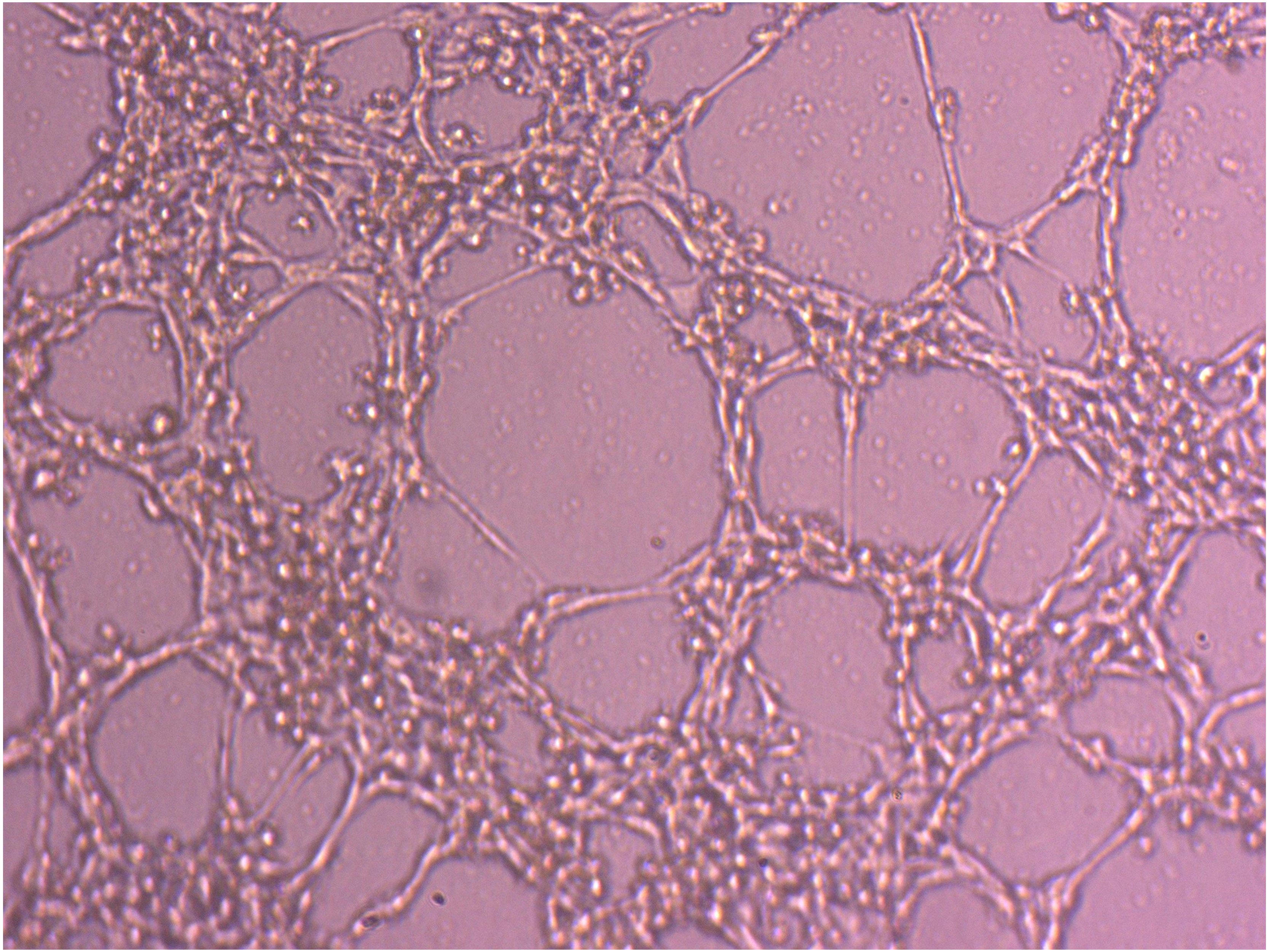

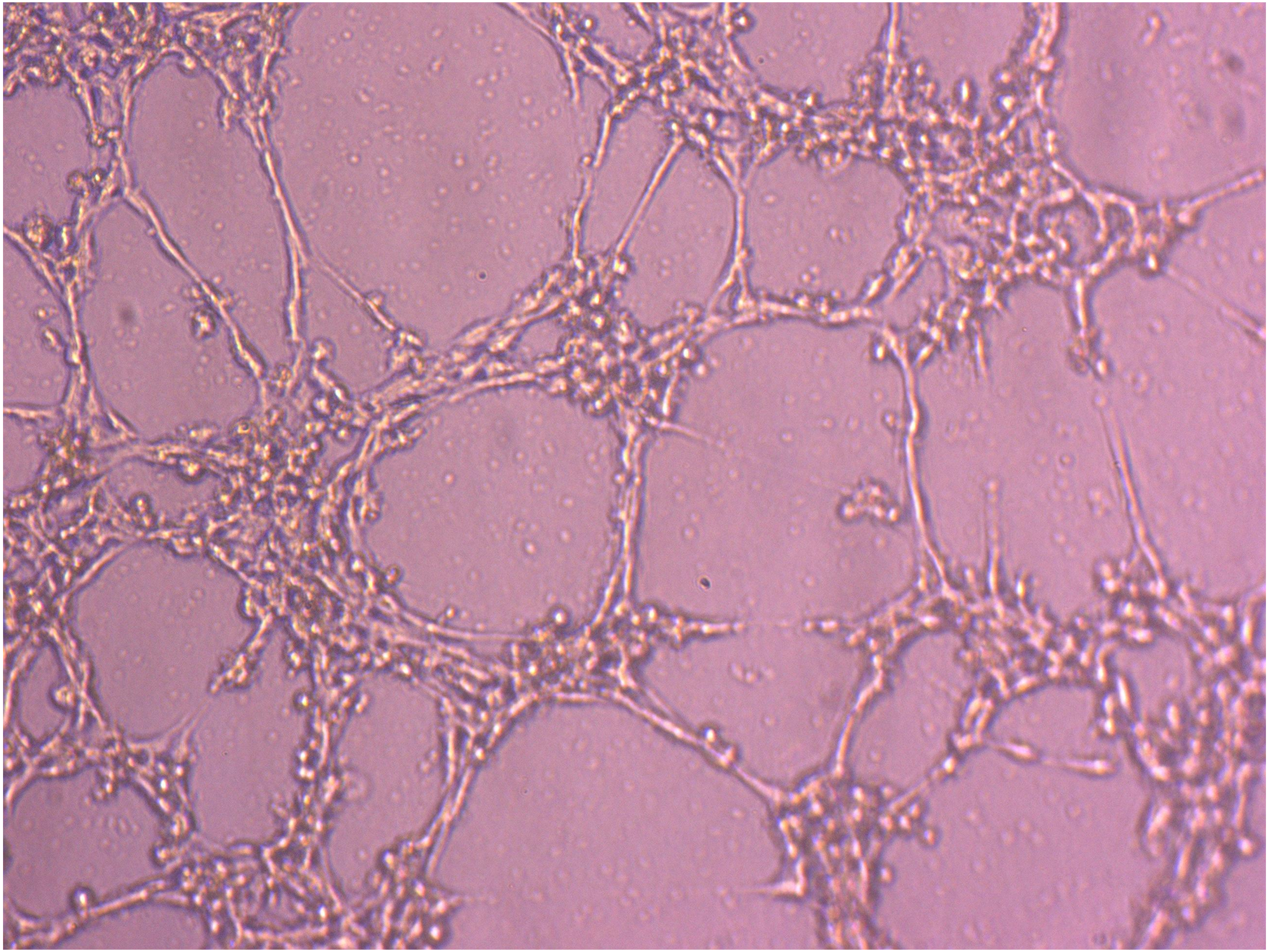

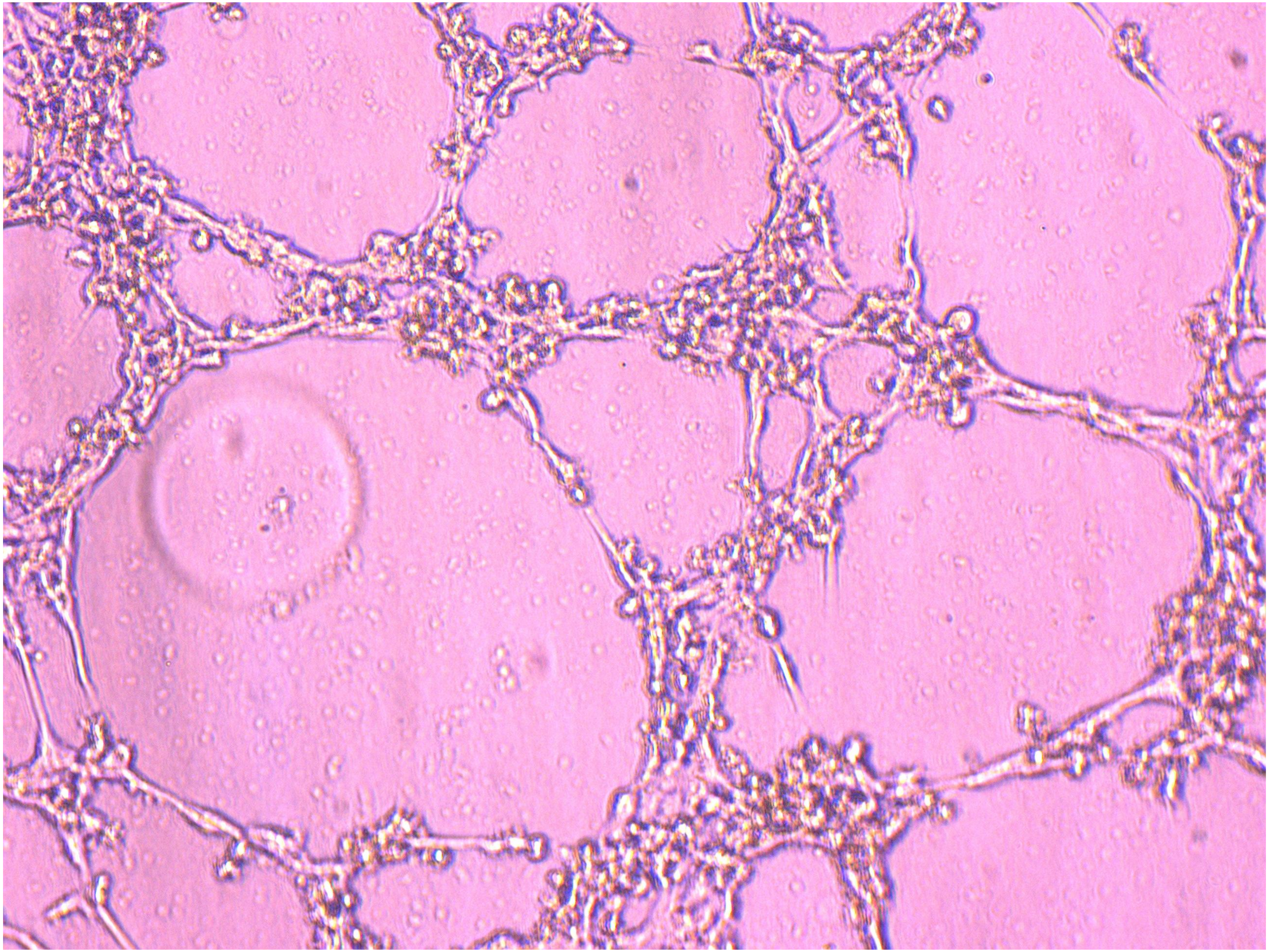

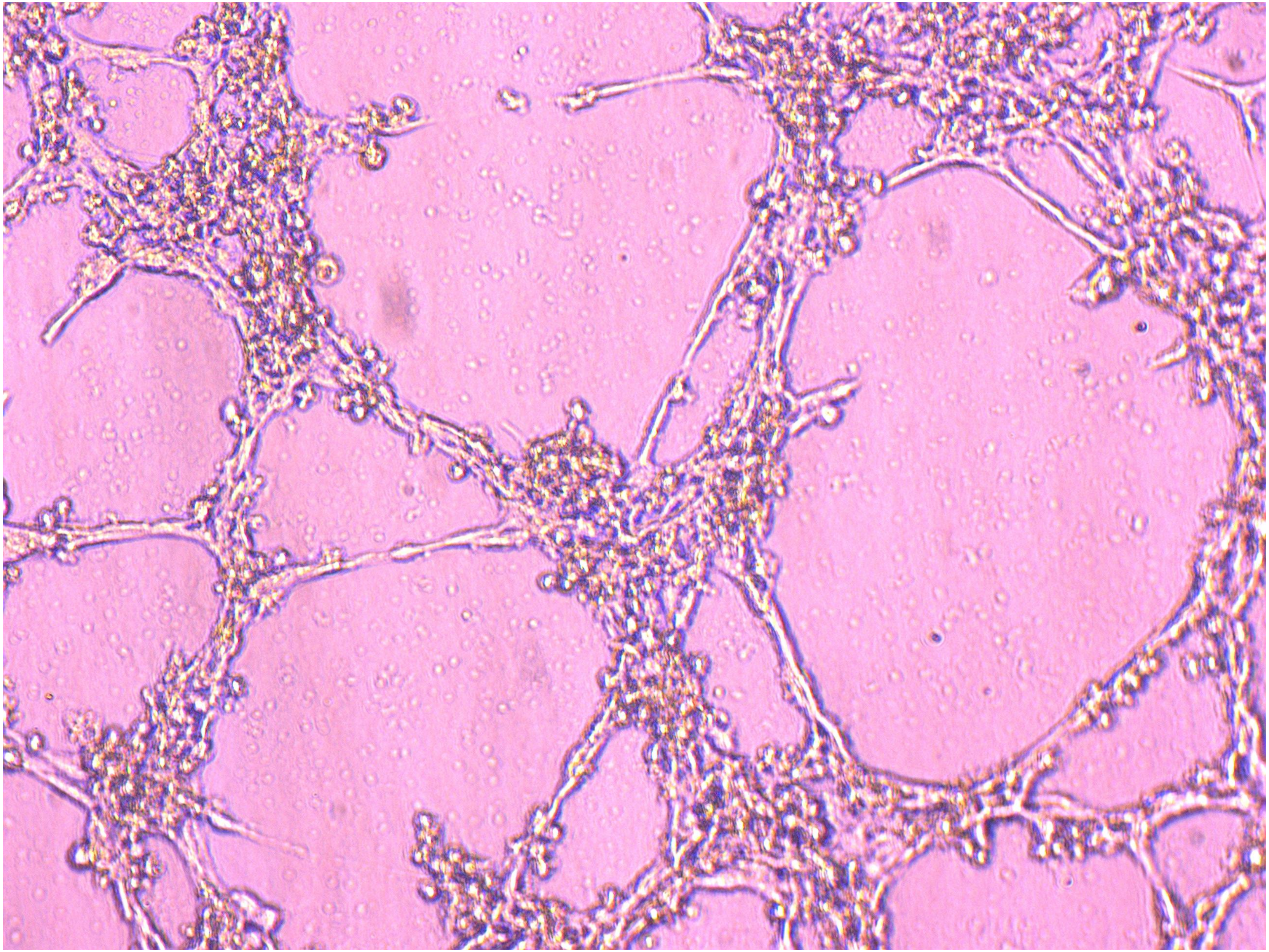

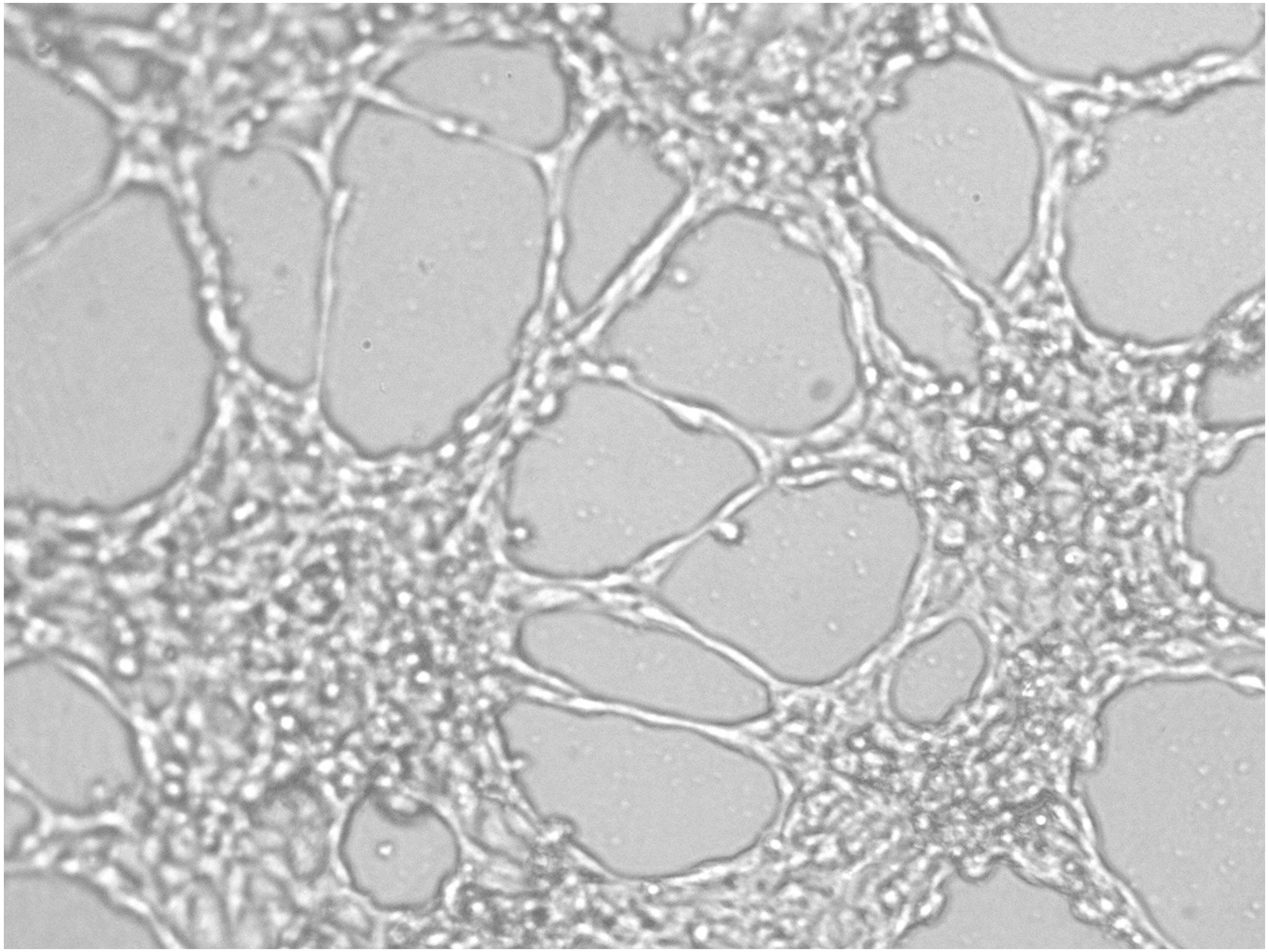

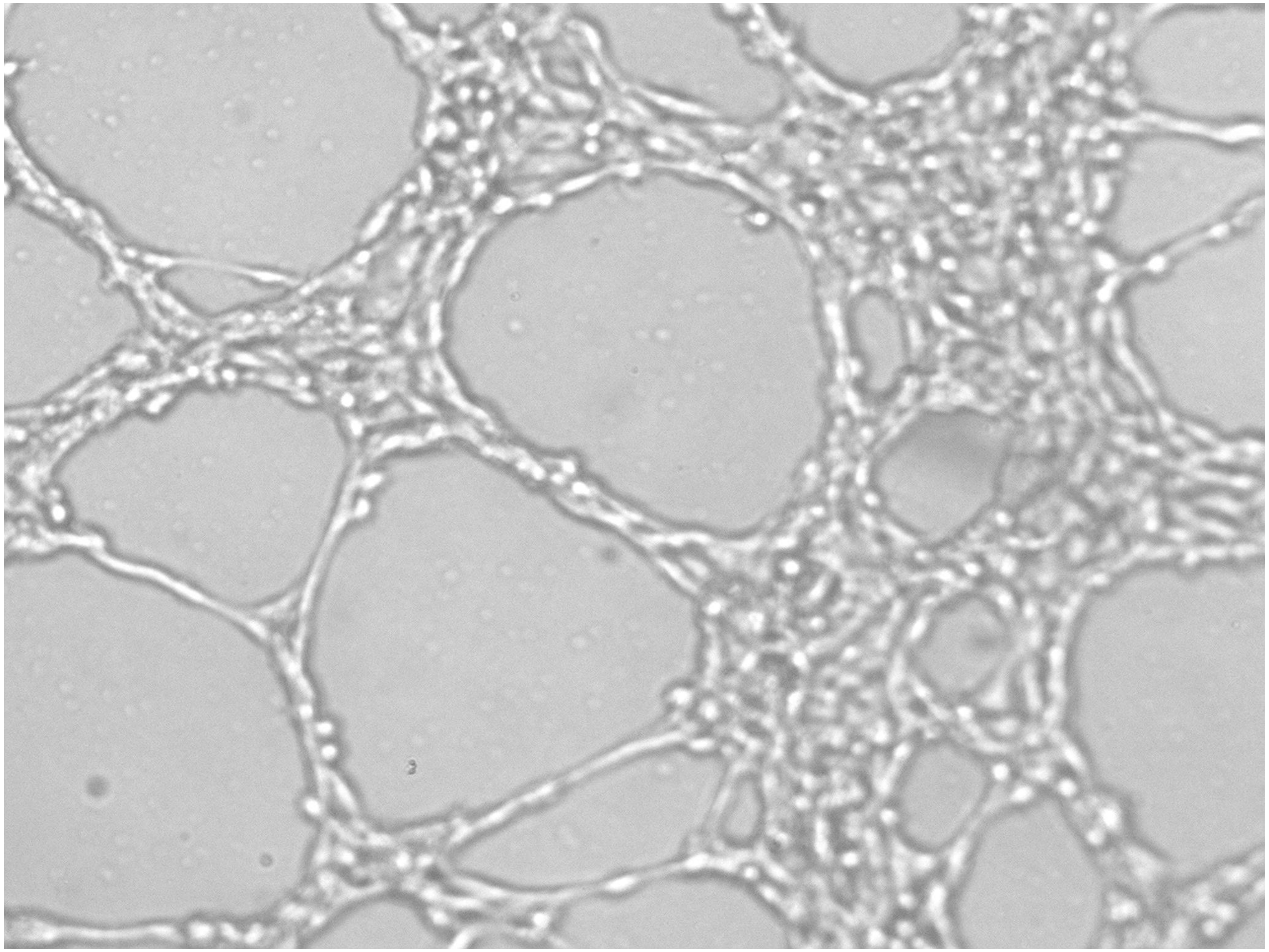

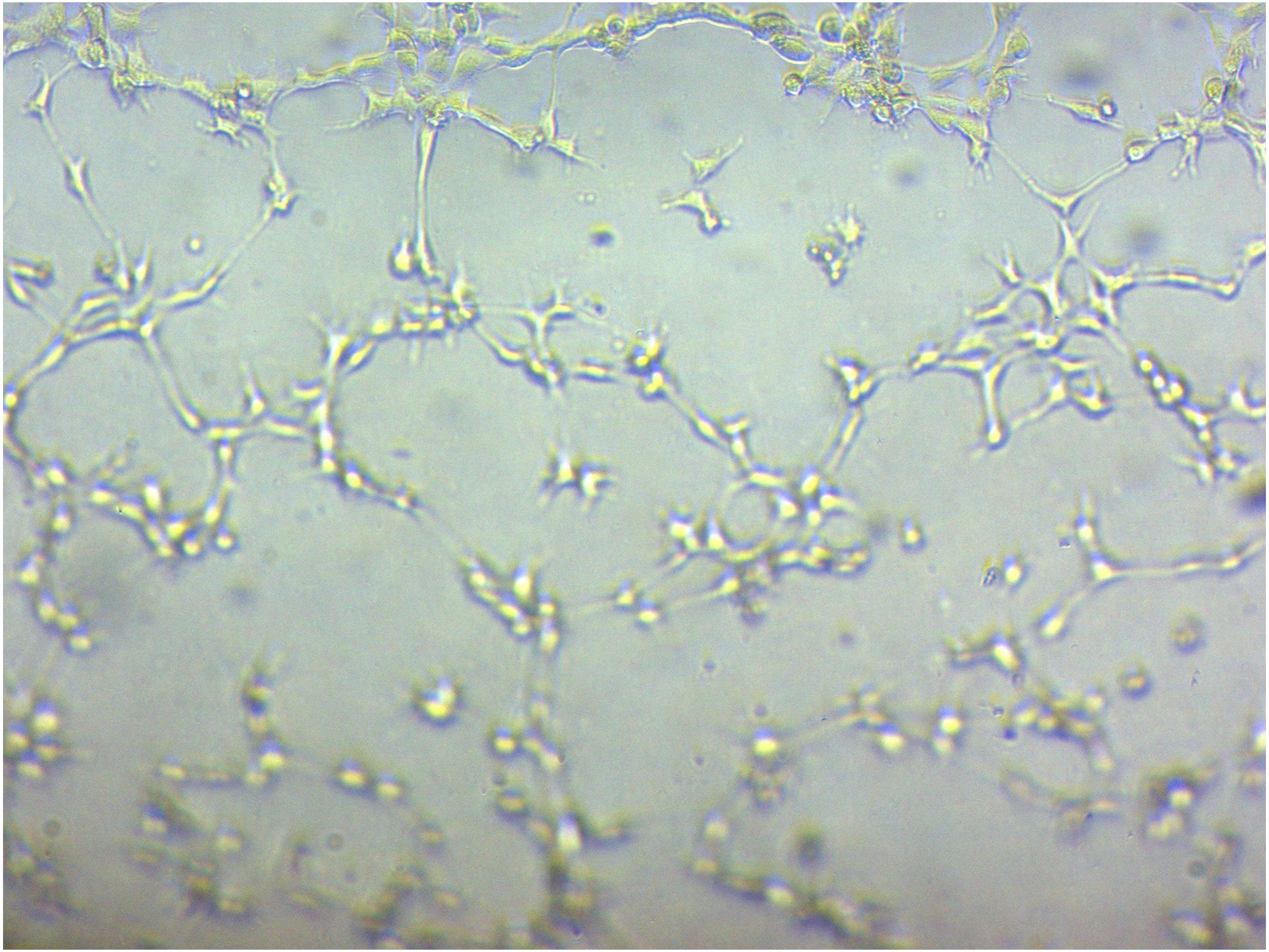

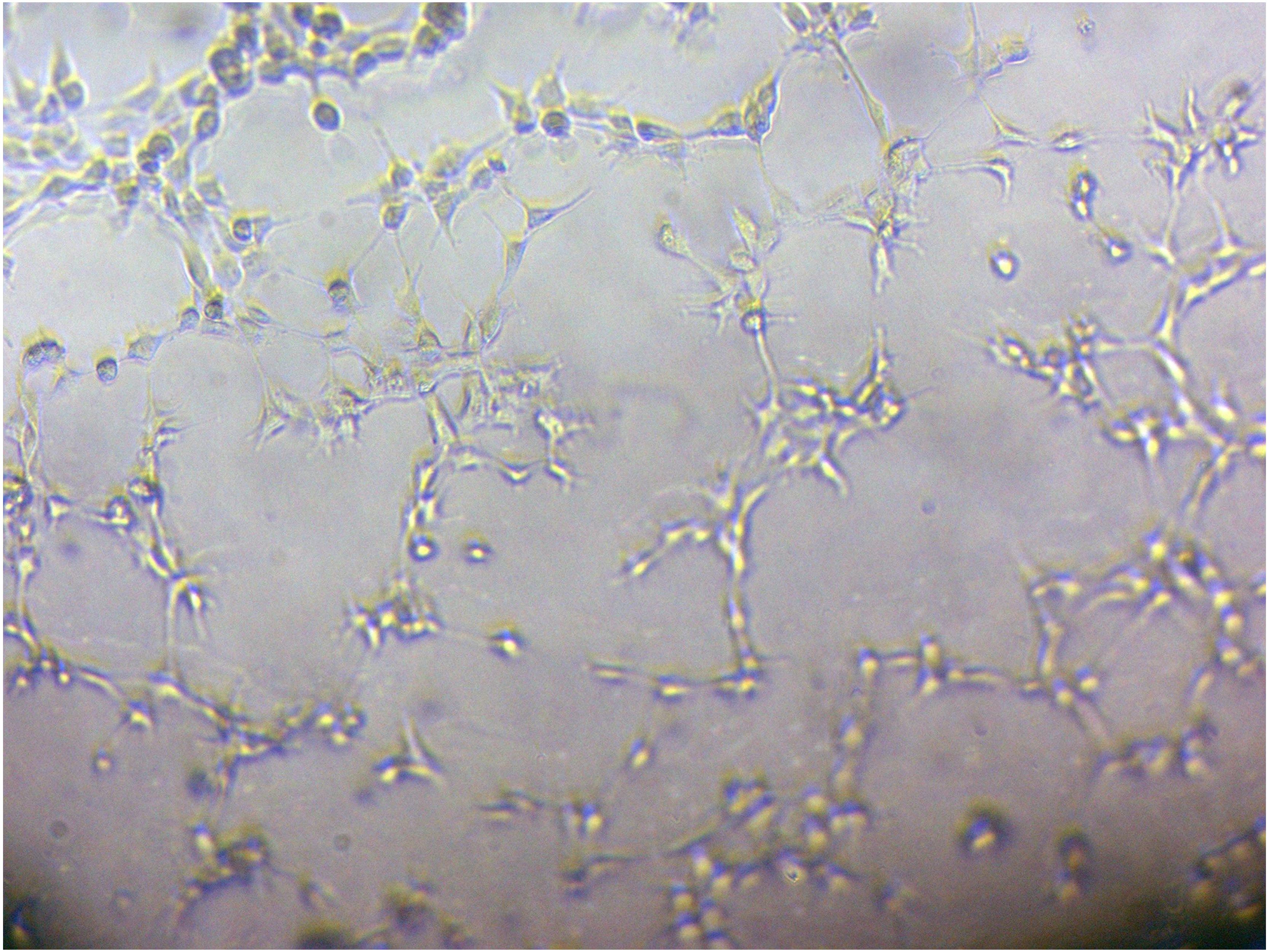

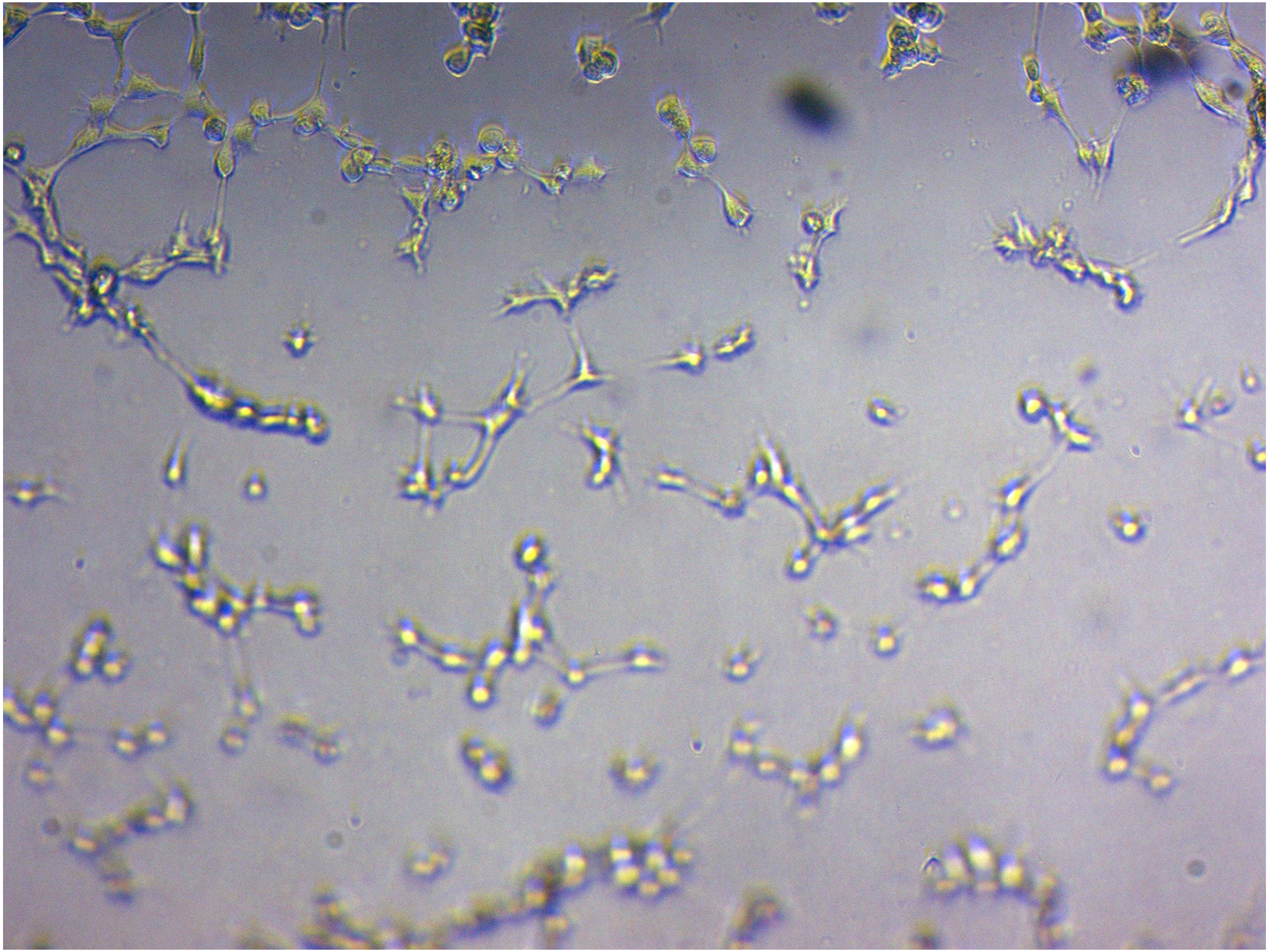

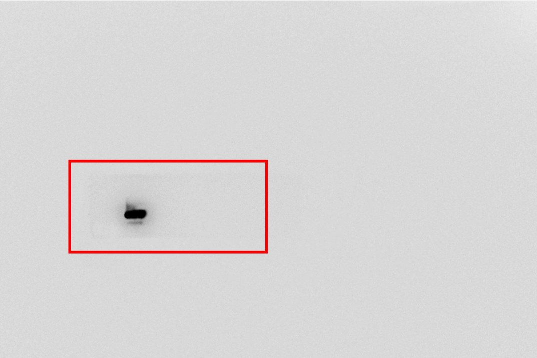
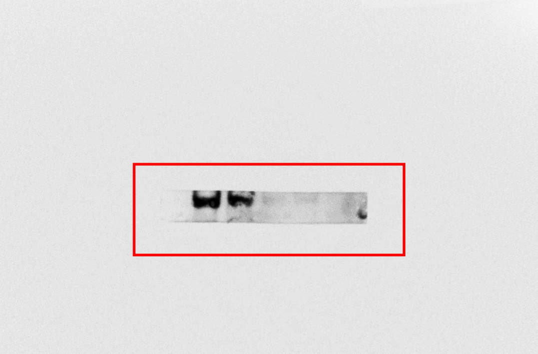
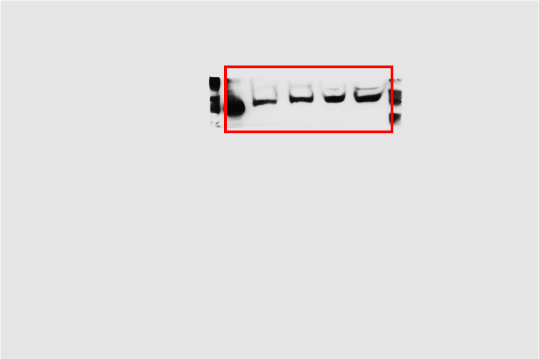
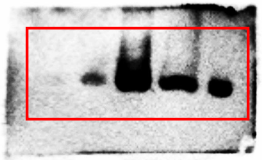
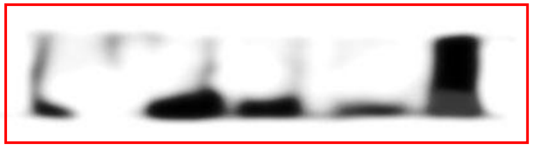
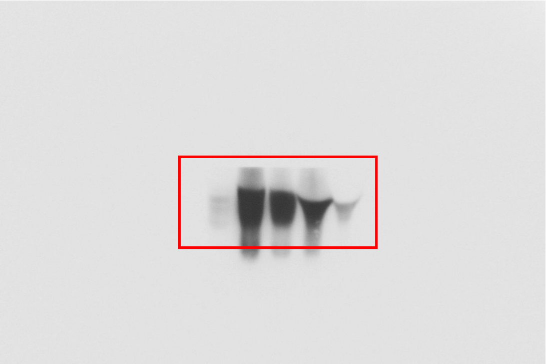
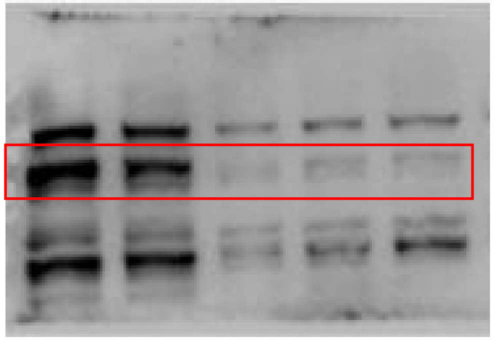
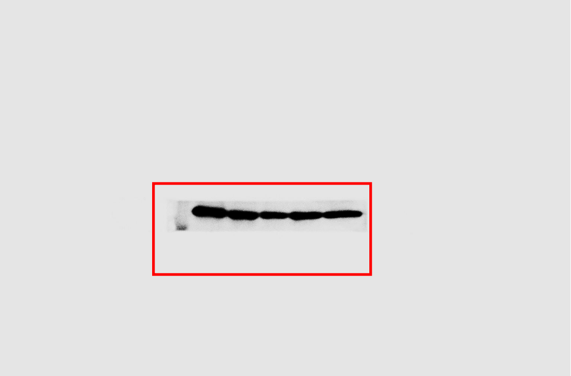
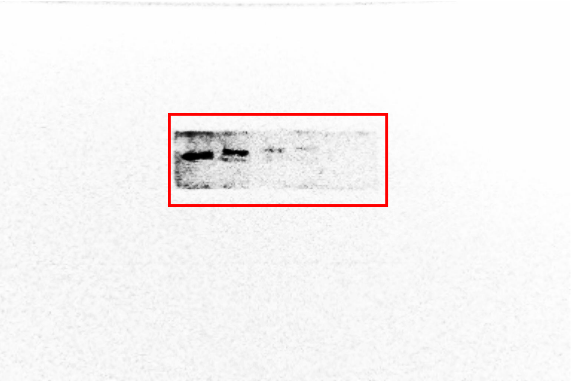
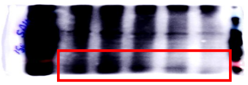

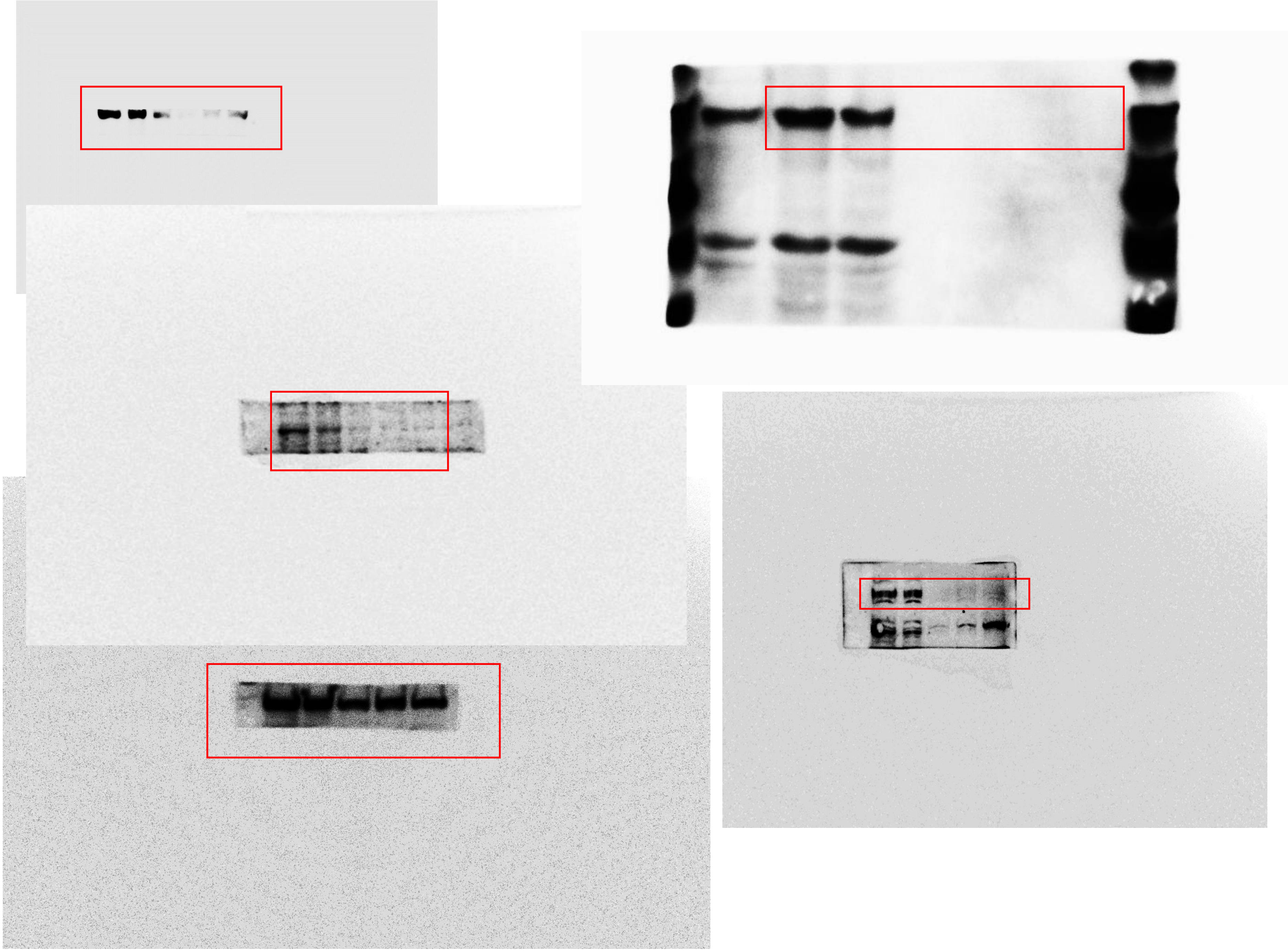

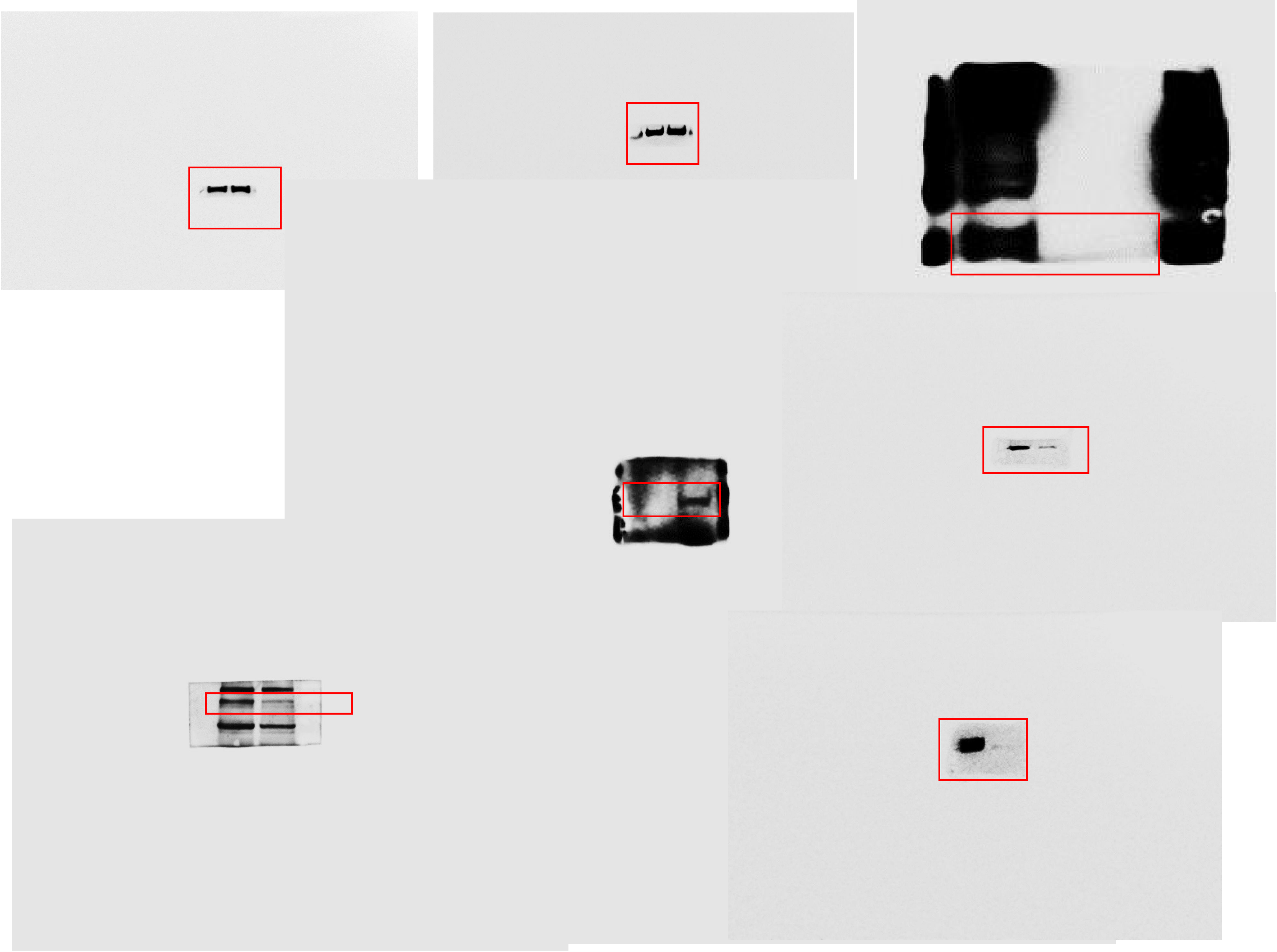

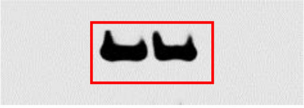
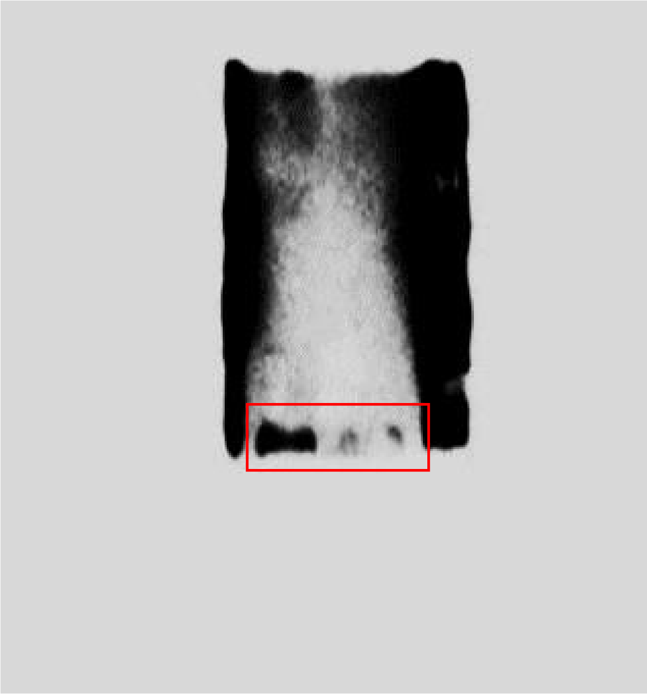
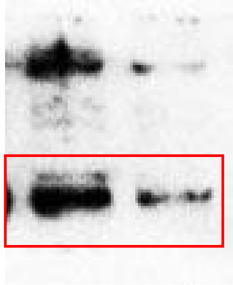
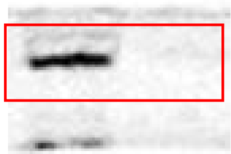
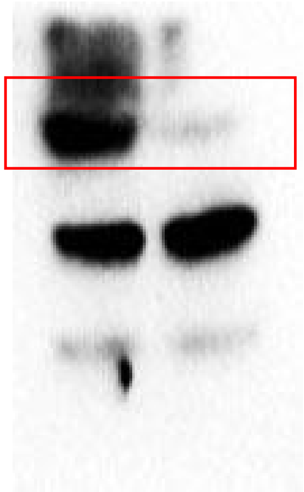

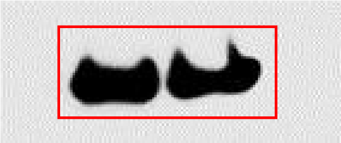

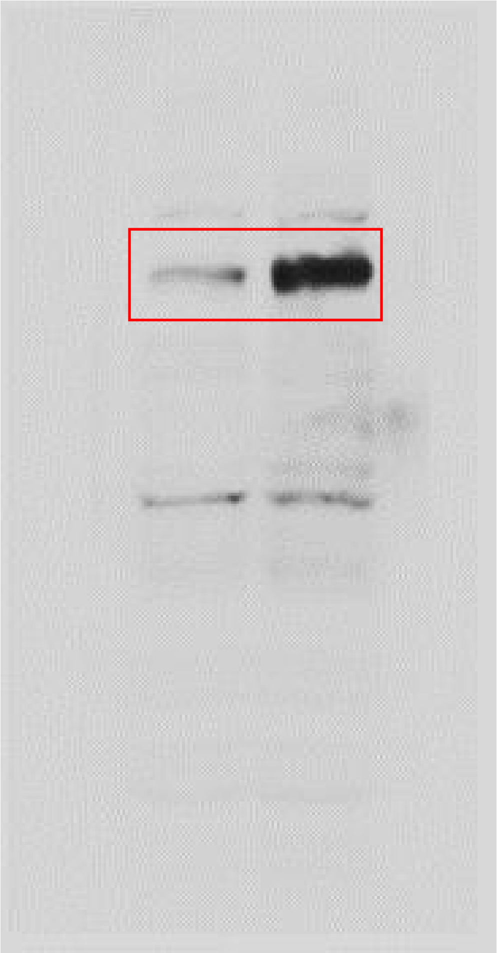

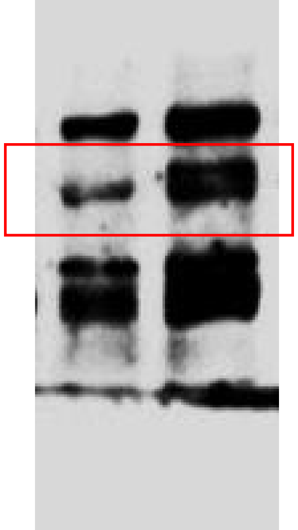

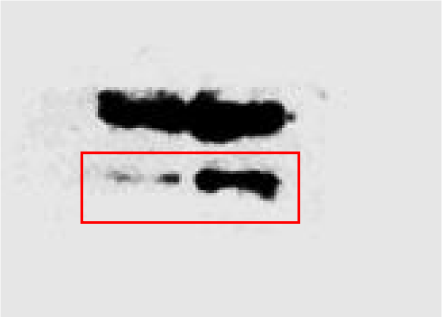

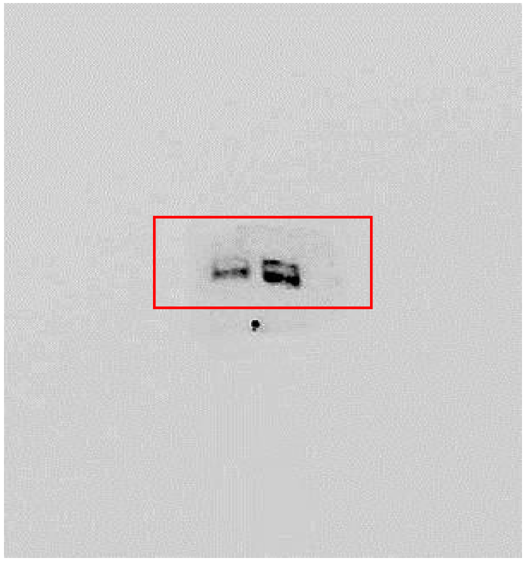

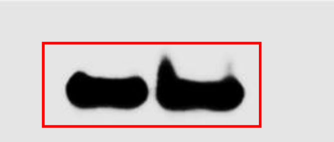

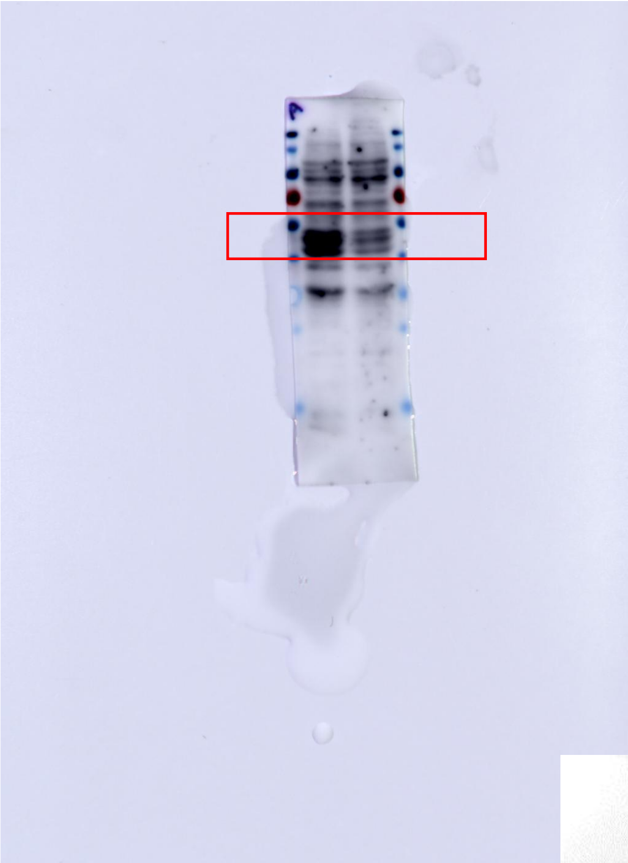

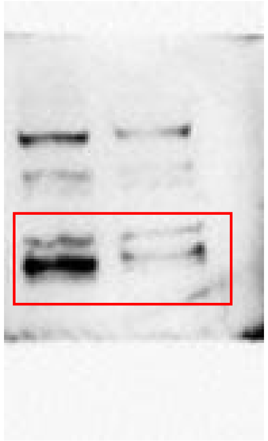

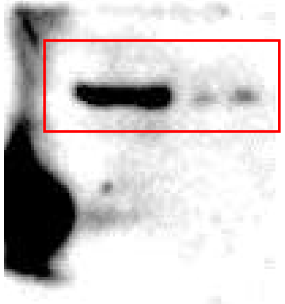

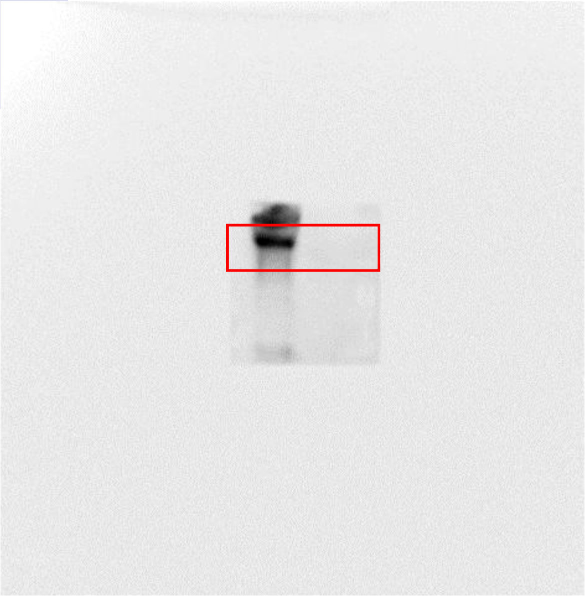

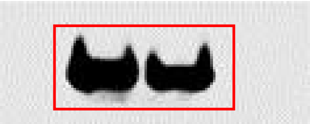

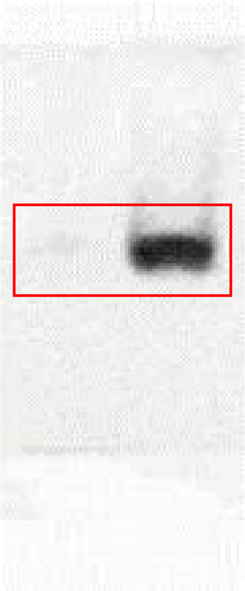

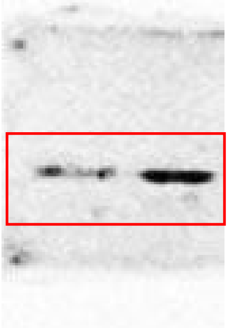

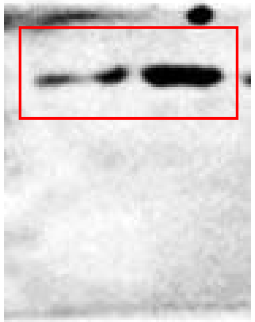

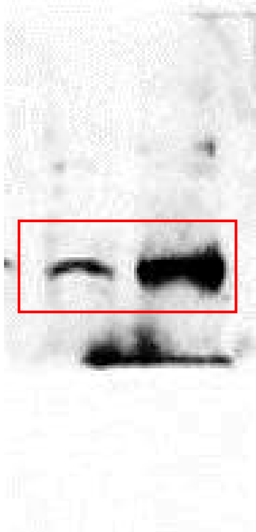

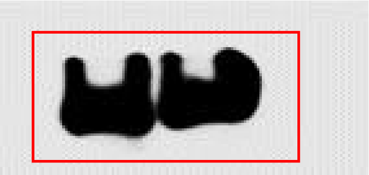
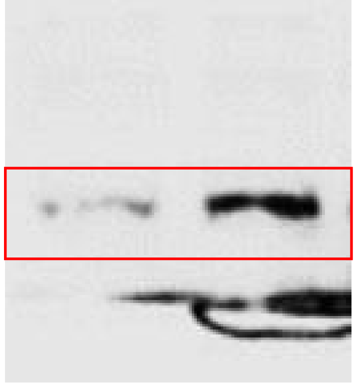
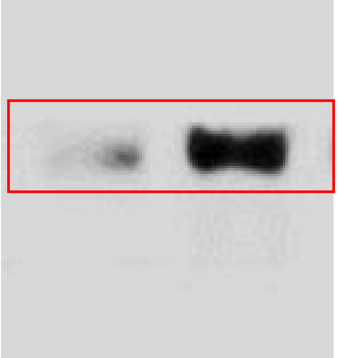
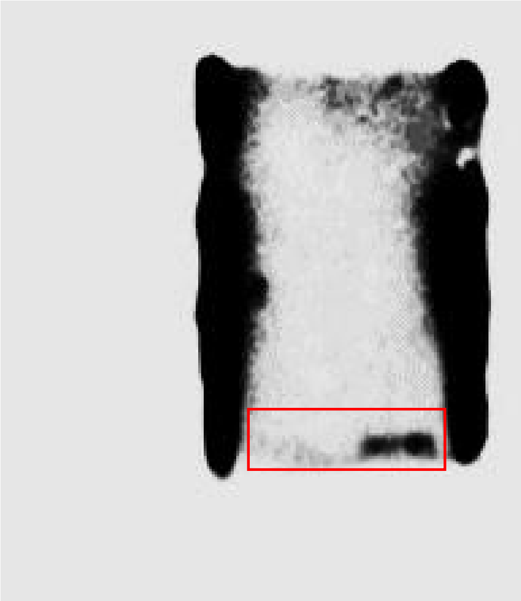
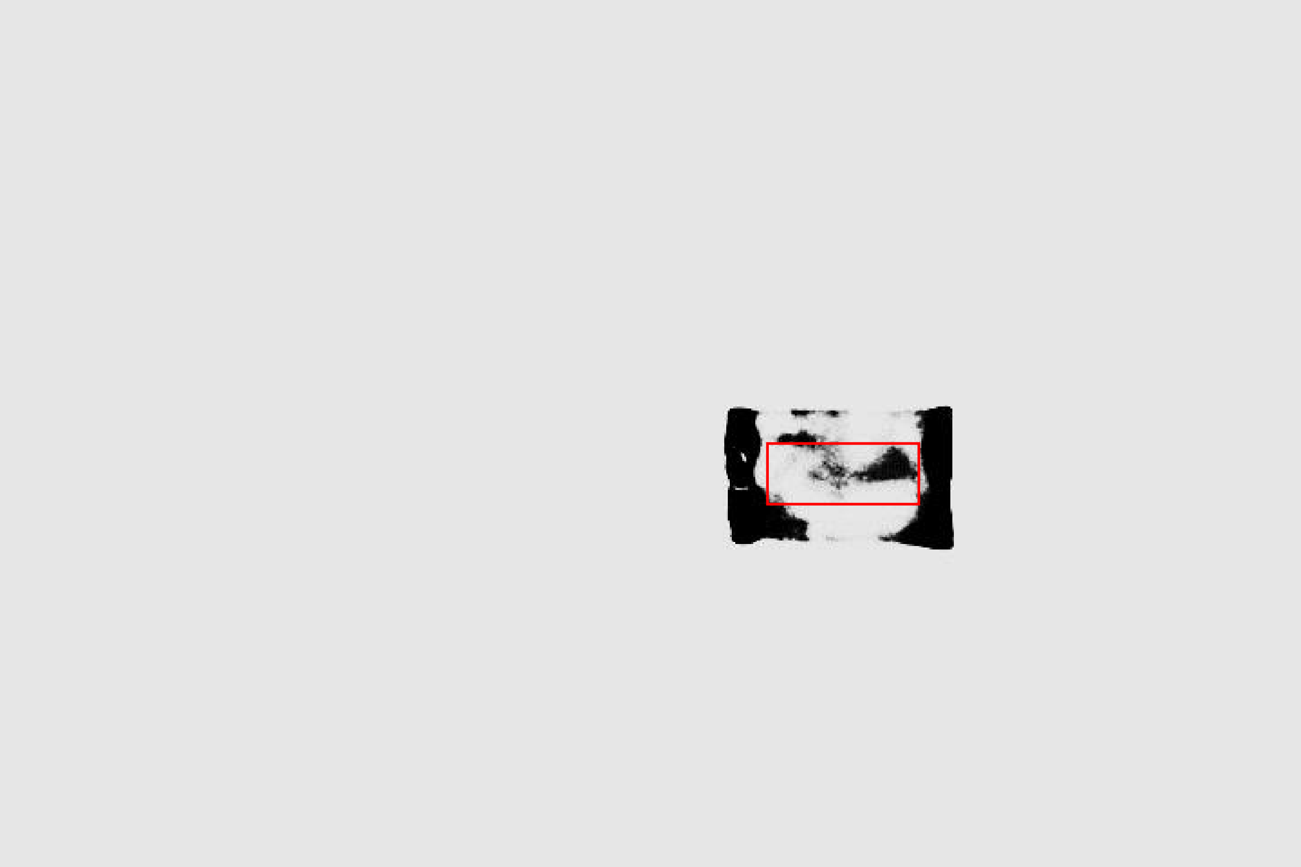

Supplement: Supplementary file 2 — Additional file 2: Sequences of the putative SALL4-binding site. [file 12935_2023_2985_MOESM2_ESM.pdf]
